# DNMT1 modulation of RASSF1A methylation enhances breast cancer brain metastasis

**DOI:** 10.1038/s41419-025-08167-x

**Published:** 2025-12-11

**Authors:** Qianqian Cao, Gongwen Xu, Guanghui Huang, Sheng Li, Hao Li, Xiaonan Zhang, Xiaomei Li

**Affiliations:** 1 Cancer Center, The Second Hospital, Cheeloo College of Medicine, Shandong University, Jinan, PR China; 2 School of business, Shandong Jianzhu University, Jinan, PR China; 3 Department of Oncology, Shandong Provincial Hospital Affiliated to Shandong First Medical University, Jinan, PR China; 4 Shandong First Medical University &Shandong Academy of Medical Sciences, Jinan, PR China; 5 Shandong Institute of Industrial Technology, Jinan, PR China; 6 Shandong Provincial Hospital Affiliated to Shandong First Medical University, Jinan, PR China; 7 Tumor Research and Therapy Center, Shandong Provincial Hospital Affiliated to Shandong First Medical University, Jinan, PR China; 8 JiNan Key Laboratory of Basic and Clinical Translational Research in Radiobiology, Jinan, PR China

**Keywords:** Cancer, Diseases

## Abstract

Epigenetic modifications, particularly DNA methylation, play a critical role in the metastatic progression of breast cancer. This study investigates the role of DNA methyltransferase 1 (DNMT1) in regulating the methylation of RASSF1A and its contribution to breast cancer brain metastasis. Using single-cell transcriptome sequencing of CTCs derived from in situ breast cancer models, DNMT1 was identified as a key promoter of tumor cell viability, migration, and invasion. RASSF1A was further validated as a direct target of DNMT1-mediated methylation. This regulatory interaction was confirmed through DNA methylation sequencing, methylation-specific PCR, and chromatin immunoprecipitation assays. These findings highlight the pivotal role of DNMT1-driven RASSF1A methylation in facilitating breast cancer metastasis to the brain, and suggest that targeting DNMT1 may represent a promising therapeutic approach for managing metastatic breast cancer.

## Background

Breast cancer remains one of the most common malignant tumors affecting women worldwide, with brain metastases significantly contributing to poor prognosis [1, 2]. As the leading cause of cancer-related morbidity and mortality among women [3], advances in early diagnosis and treatment have improved overall survival (OS). However, brain metastasis continues to pose a major therapeutic challenge, being closely associated with disease recurrence, treatment resistance, and poor clinical outcomes [4]. Therefore, elucidating the molecular mechanisms underlying breast cancer brain metastasis is critical for predicting metastatic potential and identifying therapeutic targets. In recent years, increasing attention has been directed toward understanding the early events of breast cancer brain metastasis. Despite growing interest, the underlying molecular mechanisms remain poorly defined [5–7].

Circulating tumor cells (CTCs), which detach from primary tumors or metastatic lesions and enter the bloodstream, have emerged as key players in metastatic progression [8]. These cells are considered essential precursors to distant metastases, including those in the brain [9]. Studying CTCs may therefore offer valuable insights into the mechanisms driving metastasis, yet the specific relationship between CTCs and brain metastases in breast cancer remains insufficiently understood.

DNA methylation is a fundamental epigenetic mechanism implicated in tumor initiation, progression, and metastasis. Altered methylation patterns can modulate gene expression, thereby influencing cancer development. As such, DNA methylation has gained recognition as a potential biomarker for cancer prevention, diagnosis, and therapy [10–13]. In breast cancer, methylation signatures have shown potential in early detection, treatment monitoring, prognosis, and therapeutic response prediction [14, 15].

DNA methyltransferase 1 (DNMT1) is a key enzyme responsible for maintaining DNA methylation patterns and has been shown to play a pivotal role in cancer epigenetics [16]. Elevated DNMT1 expression in breast cancer has been linked to increased tumor aggressiveness and metastatic capacity [17, 18]. Notably, recent studies have demonstrated that DNMT1 promotes breast cancer progression, influencing tumorigenesis and stemness [19]. RASSF1A, a well-characterized tumor suppressor gene, frequently shows reduced or absent expression in various cancers due to promoter hypermethylation. Methylation of RASSF1A in circulating free DNA has been associated with poor OS [20, 21]. In breast cancer, silencing of RASSF1A is commonly attributed to DNA methylation, yet whether this process is directly regulated by DNMT1—and how this regulation contributes to brain metastasis—remains to be elucidated [22]. Furthermore, key controversies and unresolved mechanisms in this context require further investigation.

This study seeks to address these knowledge gaps by exploring the regulatory role of DNMT1 in RASSF1A methylation and its impact on breast cancer brain metastasis. To achieve this, we leverage microfluidic technology for efficient CTC isolation and apply single-cell transcriptome sequencing to uncover gene expression heterogeneity at the single-cell level [23–25]. By integrating microfluidic sorting with single-cell sequencing, we aimed to delineate the role of DNMT1 in modulating RASSF1A methylation and to uncover potential molecular pathways involved in early breast cancer brain metastasis.

Ultimately, this research aimed to provide insights that may inform precision therapy and targeted drug development, thereby improving survival outcomes and quality of life for patients with metastatic breast cancer.

## Materials and Methods

### Ethics statement

All animal experiments were conducted in accordance with the Guide for the Care and Use of Laboratory Animals issued by the U.S. National Institutes of Health. The study was approved by the Animal Ethics Committee of Shandong Provincial Hospital Affiliated to Shandong First Medical University (Approval No. 2023-166).

### 4T1 cell culture

4T1 cells (Catalog No: CL-0007, Wuhan Punaise Life Science Co., Ltd., Wuhan, China), derived from BALB/c mice and known to closely mimic the growth and metastatic behavior of human breast cancer, were cultured in RPMI-1640 medium (Catalog No: PM150110, Wuhan Punaise Life Science Co., Ltd.) supplemented with 10% fetal bovine serum (FBS; Catalog No: C0235, Bi Yun Tian, Shanghai, China). Cells were maintained at 37 °C in a humidified atmosphere containing 5% CO_2_. The medium was replaced twice weekly, and cells from passages 4 to 8 were used for subsequent experiments [26].

### Establishment of in-situ breast cancer mouse model

To generate luciferase-expressing 4T1 cells, the pGL6-CMV-Luc plasmid (Catalog No: D2091, Beyotime, Shanghai, China) was transfected into 4T1 cells using Lipo8000^TM^ reagent (Catalog No: C0533, Beyotime) according to the manufacturer’s protocol. Transfected cells were selected with 10 μg/mL neomycin (Sigma, USA; CAS: 1405-10-3) for 48 h and cultured for at least one week to establish stable expression (LV-Luc).

For tumor inoculation, 5 × 10^4^ 4T1 cells suspended in 50 μL PBS were injected into the right fourth inguinal mammary fat pad of 5–6-week-old female NSG mice (NOD-Prkdc^scid^Il2rg^em1^/Smoc). Brain-metastatic cells (4T1-BM) were obtained by isolating tumor cells from brain lesions of NSG mice, followed by in vitro expansion and stable passaging. These cells were then injected into the same anatomical site of female BALB/c nude mice (BALB/c, nu/nu; Beijing Vital River Laboratory Animal Technology Co., Ltd., Beijing, China). Prior to tumor cell injection, mice were anesthetized via intraperitoneal injection of 3% isoflurane (Sigma; CAS: 26675-46-7). Tumor growth was monitored biweekly using caliper measurements, and tumor volume was calculated using the formula: Volume = π/6(length × width^2^). Immunodeficient nude mice were housed under specific pathogen-free (SPF) conditions at 25 °C with 50% relative humidity and free access to food and water. Thirty-five mice were designated for single-cell sequencing, and another thirty-five for CTC isolation and culture [27].

### Isolation of CTCs using microfluidic chip technology

CTCs were isolated using the ClearCell® FX1 label-free enrichment system (Shanghai Wisor Biotechnology Co., Ltd.). Blood samples were collected via cardiac puncture from euthanized tumor-bearing mice and processed following the manufacturer’s protocol. Sorted CTCs were seeded onto 5 × 5 mm glass coverslips in 6-well plates, fixed with 4% paraformaldehyde in PBS for 20 min, permeabilized with 0.1% Triton X-100 for 30 min, and blocked with 5% BSA in PBS for 1 h at room temperature. Cells were incubated overnight at 4 °C with primary antibodies against mouse Cytokeratin 8/18 (MA5-12104, 1:1000, ThermoFisher, USA) and rabbit anti-CD45 (70257, 1:100, Cell Signaling Technology, USA), followed by 1 h incubation at room temperature with secondary antibodies: Alexa Fluor 488-conjugated goat anti-rabbit IgG (ab150077, 1:1000, Abcam, UK) and Alexa Fluor 594-conjugated goat anti-mouse IgG (ab150116, 1:1000, Abcam, UK). Nuclei were counterstained with DAPI (D9542, Sigma, USA) for 10 min. Immunofluorescence imaging was performed using an inverted fluorescence microscope. The sorting efficiency, as determined by staining, was 90.5% [28, 29].

### Single-cell sequencing and data analysis

RNA libraries were prepared following the standard Illumina protocol [30]. Raw sequencing reads were filtered and demultiplexed using PISA (https://github.com/shiquan/PISA). The filtered reads were aligned to the human reference genome (hg38), and a cell-by-gene count matrix was generated using PISA. Subsequent data analysis was performed using the Seurat R package (version 4.1.1). Quality control filtering criteria included: nFeature_RNA > 500, nCount_RNA > 1,000,000, nCount_RNA < 4,000,000, and mitochondrial gene content (percent.mt)<5%. Normalization was performed using the LogNormalize function. Principal component analysis (PCA) was conducted to reduce dimensionality, and significant principal components were selected for Uniform Manifold Approximation and Projection (UMAP) clustering. Cell annotation was performed by identifying lineage-specific marker genes and referencing the CellMarker online database for cluster annotation [31, 32].

### CTC culture

CTC-derived cell lines were cultured in ultra-low attachment plates using RPMI 1640 medium (Catalog No: PM150110, Wuhan Punuosi Life Science & Technology Co., Ltd., Wuhan, China), supplemented with EGF (20 ng/mL; 92708ES80, Yisheng Biotechnology Co., Ltd., Shanghai, China), bFGF (20 ng/mL; 91330ES76, Yisheng Biotechnology Co., Ltd.), B27 supplement (60703ES10, Yisheng Biotechnology Co., Ltd.), and antibiotics/antimycotics. Cells were maintained under hypoxic conditions (4% O_2_, 5% CO_2_). Due to the slow proliferation rate of CTCs, cultures were maintained for 8 to 12 months [9].

### Construction of CTC-derived brain metastasis mouse model

CTCs expressing HER2^+^/EGFR^+^/HPSE^+^/Notch1^+^ were identified as brain metastasis-associated markers [8]. Microfluidic chip-based sorting was employed to isolate this subpopulation. For antibody labeling, HPSE (MA5-16130, 1:50, ThermoFisher, USA) was conjugated with a Cy5-labeled reagent kit (ab188288, Abcam, UK) according to the manufacturer’s instructions. CTCs were sequentially stained with HER2 (FITC; BMS120FI, 1:50, ThermoFisher, USA) and EGFR (PE; MA5-28544, 1:50, ThermoFisher, USA), followed by fluorescence-activated cell sorting (FACS) to isolate HER2⁺/EGFR⁺ cells. This population was further labeled with HPSE and Notch1 (APC; 17-9889-42, 1:50, ThermoFisher, USA) antibodies to identify and isolate double-positive HPSE⁺/Notch1⁺ cells. The resulting HER2^+^/EGFR^+^/HPSE^+^/Notch1^+^ CTC population was confirmed by immunostaining (Fig. S1A). To establish the brain metastasis model, 1 × 10^5^ luciferase-labeled HER2^+^/EGFR/HPSE^+^/Notch1^+^ CTCs were injected intracardially into 5–6-week-old female BALB/c nude mice. Mice were administered D-luciferin (150 mg/kg), and brain metastasis formation was monitored weekly using the IVIS Lumina LT imaging system, successfully confirming model establishment (Fig. S1B). B27 supplement (50×) was added to the tumorsphere culture medium. HER2⁺/EGFR⁺/HPSE⁺/Notch1⁺ CTCs were resuspended in 5 mL of 1× PBS and gently mixed. A 20 μL aliquot was combined with 20 μL trypan blue (1:1), and 10 μL of the mixture was loaded onto a hemocytometer for counting. The required number of cells was adjusted with tumorsphere medium to a final concentration of 1 cell/μL, kept on ice, and mixed well before plating. Each well of a 96-well plate was seeded with 200 μL of the suspension (200 cells per well). The plate was sealed with laboratory tape to prevent evaporation and incubated at 37 °C with 5% CO₂ for one week [33]. HER2⁺/EGFR⁺/HPSE⁺/Notch1⁺ CTCs successfully formed tumorspheres (Fig. S1C).

### Collection of primary breast tumor and brain metastasis samples

Mice were anesthetized with 3% isoflurane (R012876, Shanghai Yien Chemical Technology Co., Ltd., Shanghai, China). For primary tumor samples, mammary tumors were surgically excised. For brain metastasis samples, entire brains were carefully dissected and immediately snap-frozen in liquid nitrogen for preservation [34, 35].

### Acquisition of high-throughput transcriptome sequencing data from breast cancer in situ and brain metastasis samples

Immediately after collection, breast cancer in situ and brain metastasis samples were transported to the laboratory for processing and RNA extraction. Total RNA was extracted from each sample using TRIzol reagent (16096020, Thermo Fisher, USA) according to the manufacturer’s instructions. RNA concentration, purity, and integrity were assessed using the Qubit® 2.0 Fluorometer® (Q33216, Life Technologies, USA) with the Qubit® RNA Analysis Kit (HKR2106-01, Shanghai Bogoo Biotechnology Co., Ltd.), the NanoPhotometer (IMPLEN, USA), and the RNA Nano 6000 Kit for the Agilent Bioanalyzer 2100 system (5067-1511, Agilent). A total of 3 μg RNA per sample was used as input for RNA library preparation. Complementary DNA (cDNA) libraries were generated using the NEBNext® Ultra^TM^ RNA Library Prep Kit for Illumina® (E7435L, NEB, Beijing), and their quality was assessed with the Agilent Bioanalyzer 2100 system. Following the manufacturer’s protocol, index-coded libraries were clustered using the TruSeq PE Cluster Kit v3-cBot-HS (PE-401-3001, Illumina) on the cBot Cluster Generation System. Sequencing was then performed on the Illumina HiSeq 550 platform [36].

### Quality control of high-throughput sequencing data

The quality of the raw paired-end reads was evaluated using FastQC software (v0.11.8). Adapter sequences and poly(A) tails were trimmed using Cutadapt (v1.18). Reads containing more than 5% ambiguous bases (N content) were removed using a custom Perl script. High-quality reads, defined as those with ≥70% bases having a Phred score ≥20, were retained using the FASTX Toolkit (v0.0.13). BBMap software was employed to correct sequencing errors in paired-end reads. The filtered, high-quality reads were aligned to the human reference genome using HISAT2 (v0.7.12) [37, 38].

### Bioinformatics analysis

Differentially expressed genes (DEGs) between breast cancer in situ and brain metastasis samples were identified using the “limma” package in R. Thresholds were set at |log2 fold change | > 2 and *P*-value < 0.05. Volcano plots were generated using the ggplot2 R package, while heatmaps were constructed using the pheatmap package. Venn diagram analyses were performed using the Xiantao Academic online tool [39].

### Acquisition of high-throughput whole-genome methylation sequencing data from breast cancer in situ and brain metastasis samples

Genomic DNA was extracted using a genomic DNA extraction kit (YDP304, Tiangen Biotech, Beijing) according to the manufacturer’s instructions. DNA quality and concentration were assessed using the NanoDrop 8000 spectrophotometer (ND-8000-GL, Thermo Fisher, USA). DNA was then randomly fragmented into 200–300 bp fragments using the Bioruptor Pico sonicator (B01060010, Diagenode). Bisulfite conversion was performed using the EpiTect Fast DNA Bisulfite Kit (59104, Qiagen). Libraries were constructed with the TruSeq DNA Methylation Kit (15066014, Illumina) following the manufacturer’s protocol. Library quality was evaluated using the Agilent 2100 Bioanalyzer to ensure appropriate insert size and concentration. Paired-end sequencing (150 bp) was conducted on the Illumina NovaSeq 6000 platform. Initial quality control was carried out using FastQC, and subsequent alignment, methylation level estimation, and site-specific methylation detection were performed using Bismark [40].

### Genome-wide methylation sequencing data analysis

Differentially methylated regions (DMRs) were identified from genome-wide methylation data to detect genes with significant methylation differences between breast cancer in situ and brain metastasis samples. These differentially methylated genes were integrated with transcriptome data, and the MethylMix package in the R environment was used to perform a Beta mixture model analysis, enabling the identification of methylation-driven genes that were negatively correlated with gene expression [41, 42].

### Viral vector infection

The lentiviral overexpression vector pCDH-CMV-MCS-EF1α-copGFP (oe-; CD511B-1, System Biosciences, USA) and interference vectors pSIH1-H1-copGFP-puro (sh-; SI501A-1, System Biosciences, USA) and pSIH1-H1-copGFP-kana (sh-; a modified version of SI501A-1) were used to construct lentivirus-mediated overexpression or silencing vectors targeting DNMT1, as well as a silencing vector for RASSF1A (Table S1). Lentiviral particles were produced in HEK-293T cells (CRL-3216, ATCC, USA) using a lentiviral packaging kit (A35684CN, Invitrogen, USA). Viral supernatants were collected 48 h post-transfection, yielding lentivirus with a titer of 1 × 10^8^ TU/mL [43, 44].

### Cell transfection and grouping

4T1 cells were cultured to approximately 50% confluence and subsequently infected with the prepared lentivirus. After 48 h, cells were selected with 10 μg/mL puromycin (540411, Sigma, USA) for at least one week to establish stable transfectants. The cells were assigned to the following groups: sh-NC, oe-NC, sh-DNMT1, oe-DNMT1, oe-DNMT1 + 5-Aza-CdR, sh-NC + sh-NC, sh-DNMT1 + sh-NC, and sh-DNMT1 + sh-RASSF1A. For co-transfections involving two shRNA vectors, constructs with puromycin and kanamycin resistance markers were used accordingly. Following transfection, cells in the designated groups were treated with the DNA methylation inhibitor 5-Aza-CdR (D9010, Beijing Soleibao Technology Co., Ltd., China) at a concentration of 5 μM for 24 h [45, 46].

### CTCs-brian metastases mouse model grouping

To establish the CTCs-brain metastasis mouse model, the pGL6-CMV-Luc plasmid was stably transfected into CTCs. These cells were then infected with the relevant lentiviral constructs (DNMT1 overexpression or knockdown, and RASSF1A knockdown vectors), resulting in the following CTC groups: sh-NC, oe-NC, sh-DNMT1, oe-DNMT1, sh-NC + sh-NC, sh-DNMT1 + sh-NC, and sh-DNMT1 + sh-RASSF1A, with six mice per group. After infection, stable CTC lines were established through puromycin selection (10 μg/mL) for at least one week.

Luciferase-labeled, stably transfected HER2^+^/EGFR^+^/HPSE^+^/Notch1^+^ CTCs (1 × 10^5^ cells) were injected into the left ventricle of 5–6-week-old female BALB/c nude mice. For in vivo bioluminescence imaging, 150 mg/kg D-luciferin was administered, and brain metastasis development was monitored weekly using the IVIS Lumina LT imaging system.

### RT-qPCR detection of target gene relative expression levels

Total RNA was extracted from tissues or cells using TRIzol reagent (15596026, Thermo Fisher, USA). RNA concentration and purity were measured at 260/280 nm using a NanoDrop LITE spectrophotometer (ND-LITE-PR, Thermo Fisher, USA). The extracted RNA was reverse transcribed into cDNA using the PrimeScript RT Reagent Kit with gDNA Eraser (RR047Q, TaKaRa, Japan), according to the manufacturer’s instructions. Quantitative real-time PCR (RT-qPCR) was conducted using the 7500 Fast Real-Time PCR System (4351106, Thermo Fisher, USA). The PCR cycling conditions were as follows: initial denaturation at 95 °C for 10 min, followed by 40 cycles of denaturation at 95 °C for 10 s, annealing at 60 °C for 20 s, and extension at 72 °C for 34 s. Gene-specific primers were synthesized by TaKaRa (Table S2), with GAPDH used as the internal reference gene. Relative gene expression levels were calculated using the 2^-ΔΔCt^ method, where ΔΔCt = (average Ct value of target gene in the experimental group - average Ct value of reference gene in the experimental group) - (average Ct value of target gene in the control group - average Ct value of reference gene in the control group) [47–49]. All RT-qPCR analyses were performed in triplicate.

### Western blot

Total protein was extracted from tissues using RIPA lysis buffer supplemented with PMSF (P0013C, Beyotime, Shanghai, China). Samples were incubated on ice for 30 min and then centrifuged at 8000 g for 10 min at 4 °C. The supernatant was collected, and protein concentration was determined using a BCA Protein Assay Kit (23227, Thermo Fisher, USA). A total of 50 μg of protein per sample was mixed with 2× SDS loading buffer, boiled at 100 °C for 5 min, separated by SDS-PAGE, and transferred to a PVDF membrane. Membranes were blocked with 5% non-fat milk at room temperature for 1 h, followed by overnight incubation at 4 °C with primary antibodies (listed in Table S3). After three washes with TBST (10 min each), membranes were incubated for 1 h at room temperature with HRP-conjugated secondary antibodies: goat anti-rabbit IgG H&L (HRP) (ab97051, 1:2000, Abcam, UK) and rabbit anti-mouse IgG H&L (HRP) (ab6728, 1:2000, Abcam, UK). After further washing with TBST, chemiluminescence detection was performed by applying a mixture of solutions A and B from the ECL kit (abs920, Elabscience Biotechnology Co., Ltd., Shanghai, China) in a dark room. The signal was visualized using the Bio-Rad imaging system (BIO-RAD, USA). Band intensities were quantified using Quantity One software (v4.6.2), and the relative protein levels were normalized to α-Tubulin. All experiments were conducted in triplicate, and average values were reported [50].

### CCK-8

Cell proliferation was assessed using the CCK-8 assay kit (CA1210, Beijing Soleibao Technology Co., Ltd., Beijing, China). After transfection and selection, logarithmically growing cells were seeded into 96-well plates at a density of 1 × 10^4^ cells per well. CCK-8 reagent (10 μL) was added to each well every 12 h, followed by a 3-hour incubation at 37 °C. Absorbance at 450 nm was measured using a microplate reader at 72 h [51].

### Clonogenic assay

Cells from each group were washed twice with PBS (P2272, Sigma, USA) and digested with trypsin (T2600000, Sigma, USA) to obtain a single-cell suspension. After cell counting, 2 mL of cell suspension at 500 cells/mL was seeded into six-well plates and gently agitated to ensure even distribution. Cells were incubated at 37 °C in a humidified 5% CO_2_ atmosphere for 7 days until visible colonies formed. The cells were then washed twice with PBS, fixed with 4% paraformaldehyde (158127, Sigma, USA), and stained with 0.5% crystal violet (V5265, Sigma, USA) for 15 min. Colonies containing more than 50 cells were counted under a stereomicroscope [52].

### Scratch wound healing experiment

Cells in good growth condition were prepared as single-cell suspensions. After counting, 2 mL of cell suspension at 8 × 10^5^ cells/mL was seeded into six-well plates and cultured until reaching 90%–100% confluence. Two parallel straight scratches were made across the monolayer using a 200 μL pipette tip. Detached cells were removed by PBS washing, and serum-free RPMI 1640 medium was added. After 24 h of incubation, images were taken, and wound closure was quantified [53].

### Transwell assay for cell invasion

Log-phase cells were harvested, washed once with PBS and serum-free medium, and resuspended in serum-free medium at a concentration of 2 × 10^5^ cells/mL. For the invasion assay, Matrigel (356237, Corning, USA) was thawed overnight at 4 °C and diluted to 300 μL/mL with serum-free medium. A total of 100 μL of diluted Matrigel was evenly coated onto the upper surface of the PET membrane in the insert of a 24-well Transwell plate and incubated at 37 °C for approximately 3 h. The coated inserts were then dried overnight in a laminar flow hood. Subsequently, 800 μL of complete medium with 10% serum was added to the lower chamber, and 150 μL of the cell suspension was added to the upper chamber. After 24 h of incubation, the remaining medium in both chambers was discarded. Non-invading cells and Matrigel on the upper membrane surface were gently removed using a cotton swab. The membranes were fixed with 0.5 mL of 4% paraformaldehyde for 30 min, followed by staining with 0.1% crystal violet (0.5 mL) for 20 min. Membranes were then rinsed three times with PBS. Invading cells on the lower membrane surface were observed under a microscope, and five random fields (central and peripheral) were selected for cell counting, with the average number calculated [54].

### Cell cycle analysis by flow cytometry

Cell cycle distribution was assessed by flow cytometry. Briefly, cells were washed twice with cold PBS and collected by centrifugation. The cell pellets were then fixed in 70% ethanol prepared in PBS at 4 °C. After fixation, cells were washed with PBS and resuspended in cold propidium iodide (PI) staining solution containing RNase A (0.1 mg/mL) (EN0531, Thermo Fisher, USA) at a final PI concentration of 50 μg/mL. For each sample, a minimum of 1×10^4^ cells was analyzed using a FACS Calibur flow cytometer (BD Biosciences, San Jose, CA, USA). The distribution of cells in different phases of the cell cycle was calculated using CellQuest software (BD Biosciences, San Jose, CA, USA) [55].

### Hematoxylin and Eosin (H&E) staining

Brain tissue sections were deparaffinized, rehydrated through graded alcohols to water, and stained using an H&E staining kit (PT001, Shanghai Bogu Biotechnology Co., Ltd., Shanghai, China) according to the manufacturer’s protocol. Hematoxylin staining was performed at room temperature for 10 min, followed by rinsing under running water for 30–60 s. Sections were then differentiated in 1% hydrochloric acid alcohol for 30 s, rinsed again in running water, and immersed for 5 min. Eosin staining was performed for 1 min at room temperature. Dehydration was achieved through a graded ethanol series (70%, 80%, 90%, 95%, 100%), with each step lasting 1 min. Sections were cleared in xylene for 1 min and then further cleared in xylene I and xylene II, each for 1 min. Finally, slides were mounted with neutral resin in a fume hood. Tissue morphology was observed and imaged under an optical microscope (BX50, Olympus Corp., Tokyo, Japan) [56, 57].

### Immunohistochemical staining

Paraffin-embedded tissue sections were initially baked at 60 °C for 20 min. Slides were then sequentially immersed in xylene (15 min ×2), followed by absolute ethanol for 5 min twice. The sections were rehydrated through graded ethanol (95% and 70%, 10 min each). Endogenous peroxidase activity was blocked by incubation with 3% H_2_O_2_ at room temperature for 10 min. Antigen retrieval was performed by microwaving the slides in citrate buffer for 3 min, followed by cooling at room temperature for 10 min. Slides were then washed three times with PBS. Sections were blocked with normal goat serum (E510009, Shanghai Sangon Biotech Co., Ltd.) for 20 min at room temperature and incubated overnight at 4 °C with the following primary antibodies: rabbit anti-DNMT1 (ab188453, 1:100, Abcam, UK), mouse anti-RASSF1A (ab23950, 1:100, Abcam, UK), and mouse anti-PCNA (ab29, 1:10,000, Abcam, UK). After washing three times with PBS, the slides were incubated for 30 min with appropriate secondary antibodies: goat anti-rabbit IgG (ab6721, 1:1000, Abcam, UK) and goat anti-mouse IgG (ab97240, 1:200, Abcam, UK). Following PBS washes, sections were developed using DAB chromogen solution (P0203, Beyotime, Shanghai, China) for 6 min and counterstained with hematoxylin for 30 s. Dehydration was performed through an ethanol gradient (70%, 80%, 90%, 95%, and 100%, each for 2 min), followed by two rounds of xylene clearing for 5 min each. Slides were mounted with neutral resin and visualized under a brightfield microscope (BX63, Olympus, Japan). Staining intensity was quantified using ImageJ software. For each group, six mice were analyzed, with three tissue sections per mouse and five random fields per section. The average staining intensity per mouse was used as the representative value [58, 59].

### Methylation analysis

Genomic DNA was extracted using a genomic DNA extraction kit (YDP304, Tian Gen Biochemical Technology, Beijing, China) following the manufacturer’s instructions. Bisulfite conversion of genomic DNA was performed using a bisulfite conversion kit (DP215-02, Tian Gen Biochemical Technology, Beijing, China). The methylation status of the CpG island within the RASSF1A gene promoter was analyzed by methylation-specific PCR (MSP) using the EM101 kit (Tian Gen Biochemical Technology, Beijing, China). Primer sequences for methylated and unmethylated alleles are listed in Table S4. The PCR protocol included an initial denaturation at 95 °C for 5 min, followed by 35 cycles of denaturation at 94 °C for 20 s, annealing at 60 °C for 30 s, extension at 72 °C for 20 s, and a final extension at 72 °C for 5 min. MSP products were electrophoresed on a 2% agarose gel prepared in 1× TBE buffer. Band intensity was assessed using the E-Box CY5 gel imaging system (Vilber, France). A higher band intensity of methylation-specific PCR products indicated a higher methylation level of RASSF1A [60, 61].

### Chromatin immunoprecipitation (ChIP) assay

A ChIP assay was performed using the ChIP Kit (KT101-02, Saicheng Biotechnology Co., Ltd., Guangzhou, China) to evaluate the enrichment of DNMT1 at the promoter region of the RASSF1A gene. Briefly, cells at 70–80% confluence were fixed with 1% formaldehyde for 10 min at room temperature to crosslink DNA and associated proteins, stabilizing protein-DNA interactions. Chromatin was then sheared by sonication (15 cycles of 10 s on and 10 s off) to generate DNA fragments of appropriate size. After centrifugation at 13,000 rpm for 10 min at 4 °C, the supernatant was collected and divided equally into three tubes for immunoprecipitation: one with anti-RNA polymerase II antibody (positive control, ab238146, 1:100, Abcam), one with rabbit IgG (negative control, ab172730, 1:100, Abcam), and one with anti-DNMT1 antibody (target, ab188453, 1:100, Abcam). Samples were incubated overnight at 4°C with rotation. Immunocomplexes were captured using Protein Agarose/Sepharose beads, followed by brief centrifugation and removal of unbound components. After washing to eliminate nonspecific binding, DNA-protein crosslinks were reversed by overnight incubation at 65°C. Purified DNA was obtained via phenol/chloroform extraction and used as a template for qPCR to assess the enrichment of the *RASSF1A* promoter region. The primer sequences used for the *RASSF1A* promoter were: Forward: GATCACGGTCCAGCCTCTGC; Reverse: CTCGAGCCTTCACTTGGGGT [62–65].

### Statistical analysis

Statistical analyses were performed using R software (version 4.2.1) within the RStudio environment (version 2022.12.0-353). Data were visualized and processed using GraphPad Prism version 9.5. Continuous variables were expressed as mean ± standard deviation (Mean ± SD). Comparisons between two groups were conducted using the unpaired Student’s t-test, while comparisons among multiple groups were assessed using one-way analysis of variance (ANOVA). Homogeneity of variance was evaluated with Levene’s test. If variances were homogeneous, Dunnett’s *t* test or LSD-*t* test was applied for pairwise comparisons; if not, Dunnett’s T3 test was used. A *P*-value < 0.05 was considered statistically significant [66, 67].

## Results

### Microfluidic Isolation of CTCs

**Fig. 1:**
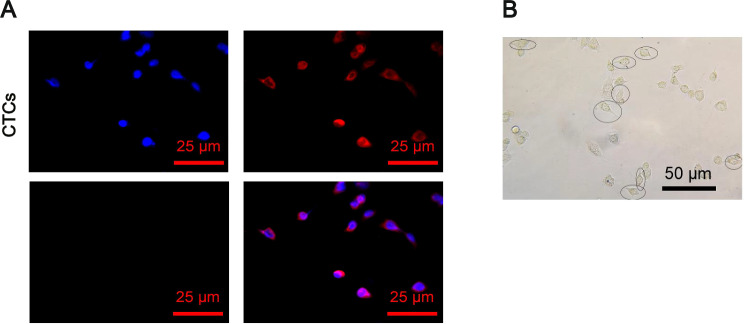
In situ selection and culture of CTCs from breast cancer mice. **A** Immunofluorescence staining to identify the purity of screened CTCs (scale bar: 25 μm). **B** Microscopic image of CTCs isolated after 48 h of culture (live CTCs indicated within white circles, scale bar: 50 μm).

Peripheral blood samples were collected from a mouse model of in situ breast cancer and processed using a microfluidic chip system for the isolation of CTCs. Following isolation, immunofluorescence staining was employed to assess CTC purity. Cells positive for epithelial markers and negative for the leukocyte marker CD45 were identified as CTCs, yielding a sorting efficiency of 90.5% (Fig. 1A). The isolated CTCs were subsequently cultured in vitro. Microscopic examination revealed characteristic features of CTCs, including increased cell and nuclear sizes and a markedly elevated nuclear-to-cytoplasmic ratio. Most CTCs appeared nearly round, though some displayed irregular, polygonal, or elongated morphologies. Additionally, multicellular clusters or aggregates were occasionally observed (Fig. 1B).

These results confirm the successful isolation and identification of CTCs using microfluidic chip technology combined with immunofluorescence staining, providing a robust experimental basis for further investigations into the molecular mechanisms underlying breast cancer brain metastases.

### Single-cell profiling of malignant CTCs and identification of marker genes

To explore the transcriptomic landscape of CTCs, single-cell RNA sequencing (scRNA-seq) was performed on the sorted cells. Data were processed and analyzed using the Seurat package. Quality control metrics included the number of detected genes per cell (nFeature_RNA), total transcript count (nCount_RNA), and the proportion of mitochondrial transcripts (percent.mt). Most cells exhibited nFeature_RNA > 500, nCount_RNA between 1,000,000 and 4,000,000, and percent.mt = 0%, indicating high data quality (Fig. S2A). Correlation analysis revealed no meaningful correlation between nCount_RNA and percent.mt (*r* = NA), while a moderate positive correlation (*r* = 0.44) was observed between nCount_RNA and nFeature_RNA (Fig. S2B), supporting the reliability of the filtered dataset for downstream analysis.

**Fig. 2:**
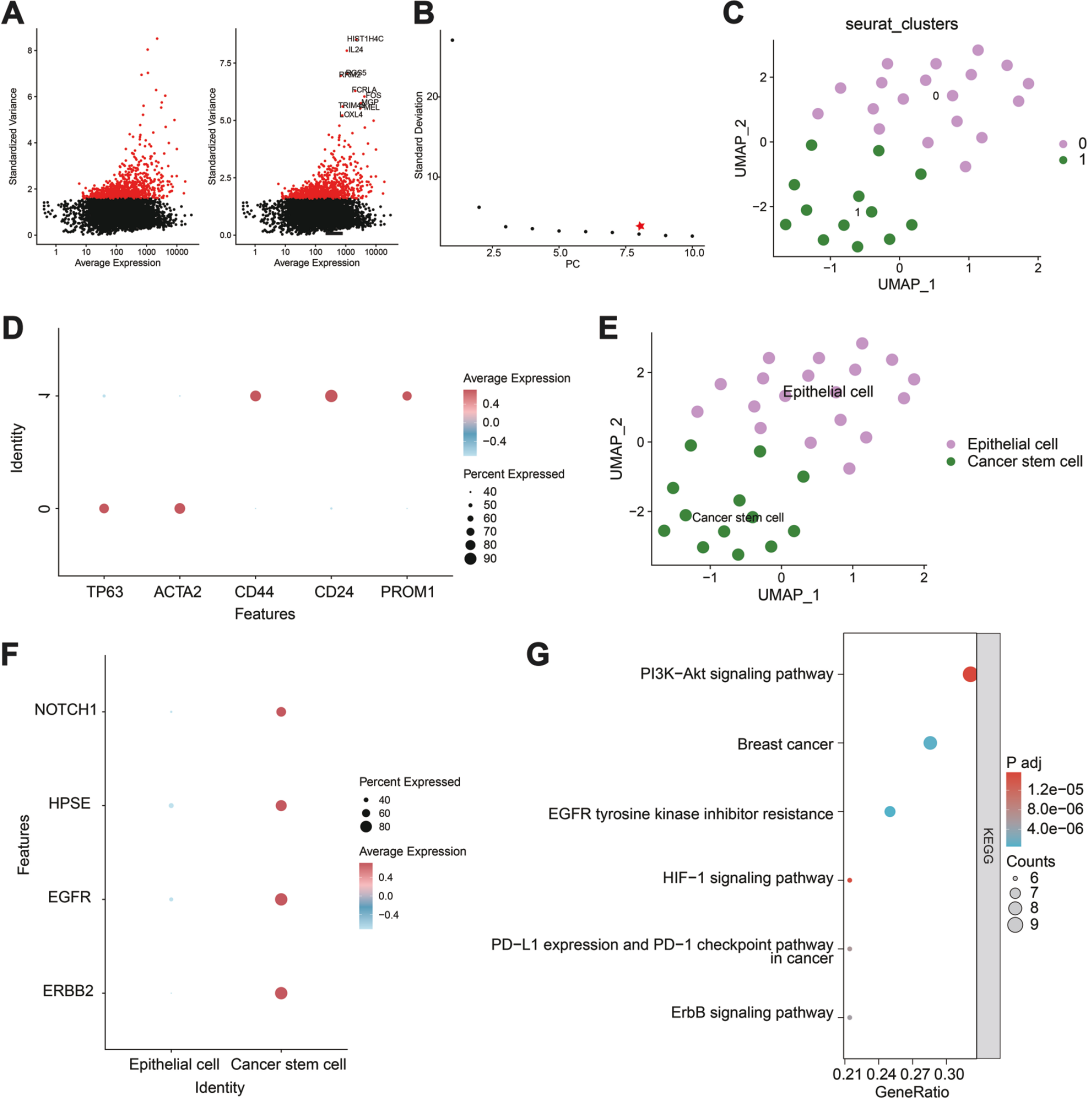
Single-cell RNA sequencing data cellular clustering and annotation. **A** Variance analysis to screen highly variable gene expressions, with red indicating the top 1000 highly variable genes and black representing genes with low variability, along with annotation of the top 10 highly variable genes. **B** Distribution of standard deviations for. PCs, where important PCs exhibit larger standard deviations. **C** Visualization of UMAP clustering results showing the aggregation and distribution of cells from different sources, with each color representing a cluster. **D** Expression patterns of known cell lineage-specific marker genes in different clusters, where deeper red indicates higher average expression levels and larger circles represent more cells expressing the gene. **E** Visualization of cell annotation results based on UMAP clustering, with each color representing a cell subgroup. **F** Expression patterns of brain metastasis selection markers in CTCs, where deeper red indicates higher average expression levels and larger circles represent more cells expressing the gene. **G** Enrichment analysis of cancer stem cell marker genes in the KEGG pathway (the x-axis represents GeneRatio, the y-axis represents KEGG functional terms, circle size in the graph indicates the number of enriched genes in that term, and color indicates the enrichment *p*-value).

Genes with the highest expression variability were selected, and the top 1000 variable genes were used for dimensionality reduction (Fig. 2A). PCA was applied, and feature loadings for the top principal components, along with heatmaps for the top six components, were generated (Fig. S2C, Fig. S2D). An ElbowPlot was used to assess the contribution of each principal component, with PC1-PC8 identified as informative and selected for further analysis (Fig. 2B).

Subsequently, UMAP based on the first eight principal components revealed two distinct clusters (Fig. 2C). Cell type annotation was performed using known marker genes referenced from the literature and the CellMarker database (Fig. 2D). Cluster 0 was annotated as cancer cells, while cluster 1 was identified as CSCs (Fig. 2E)—a subpopulation with self-renewal and differentiation capabilities, implicated in tumor initiation and progression [68].

Expression analysis of brain metastasis-selective markers (BMSM), including HER2^+^/EGFR^+^/HPSE^+^/Notch1^+^ [8], showed higher expression levels in CSCs compared to non-stem cancer cells (Fig. 2F). Furthermore, KEGG pathway enrichment analysis revealed that CSC marker genes were significantly enriched in pathways related to breast cancer, PI3K-Akt signaling, EGFR tyrosine kinase inhibitor resistance, HIF-1 signaling, PD-L1/PD-1 checkpoint regulation, and ErbB signaling (Fig. 2G).

Collectively, these findings underscore the critical role of cancer stem cells (CSCs) within CTCs. The enrichment of CSC marker genes in key breast cancer-related signaling pathways suggests their significant involvement in the metastatic process, particularly in the development of brain metastases.

### Transcriptomic analysis reveals the role of DNMT1

**Fig. 3:**
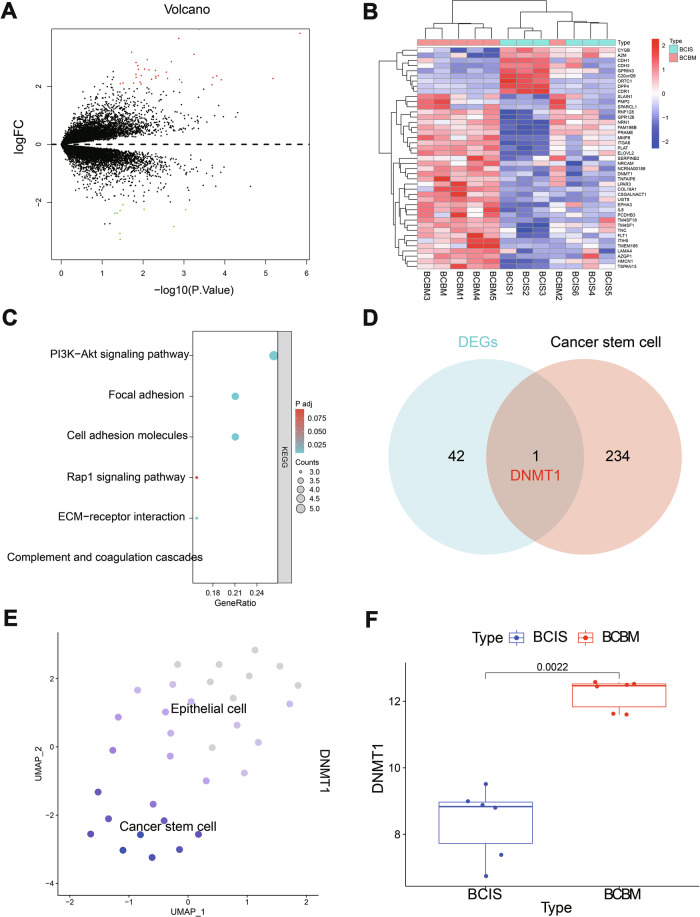
Identification of target genes involved in the occurrence of breast cancer brain metastasis (BCBM) through biological information analysis. **A**, **B** Volcano plot and heatmap of differentially expressed genes selected from transcriptome sequencing data (-log10 value on the x-axis and logFC on the y-axis; green dots represent downregulation, red dots represent upregulation, black dots indicate no significant difference; BCIS group, *n* = 6; BCBM group, *n* = 6). **C** KEGG pathway enrichment analysis of DEGs from transcriptome data (GeneRatio on the x-axis, KEGG functional terms on the y-axis, circle size in the graph represents the number of enriched genes in that term, color indicates the enrichment *p*-value). **D** Venn diagram showing the intersection of DEGs and cancer stem cell marker genes. **E** Expression pattern of DNMT1 in various cell subgroups, with darker blue indicating higher average expression levels. **F** Statistics of DNMT1 gene expression in transcriptome sequencing data (BCIS group, *n* = 6; BCBM group, *n* = 6).

To identify key genes involved in breast cancer brain metastasis, we established both in situ breast cancer and brain metastasis models and obtained tissue samples accordingly. High-throughput transcriptome sequencing was performed on both sample types. Differential expression analysis identified 43 DEGs, including 9 upregulated and 34 downregulated genes (Fig. 3A, B). KEGG pathway enrichment analysis revealed that these DEGs were predominantly enriched in the PI3K-Akt signaling pathway, Rap1 signaling pathway, extracellular matrix (ECM)-receptor interaction, focal adhesion, complement and coagulation cascades, and cell adhesion molecule-related pathways (Fig. 3C). By intersecting these DEGs with CSC marker genes identified from prior scRNA-seq data, DNMT1 was pinpointed as a core candidate gene (Fig. 3D). DNMT1 exhibited elevated expression in CSCs (Fig. 3E) and was significantly upregulated in brain metastasis samples compared to in situ breast cancer samples (Fig. 3F).

In conclusion, these findings suggest that DNMT1 may serve as a critical regulator in breast cancer brain metastasis.

### DNMT1 enhances the malignant phenotype of breast cancer cells in vitro

To explore the functional role of DNMT1 in breast cancer, the 4T1 cell line was selected for in vitro experiments. DNMT1 was either overexpressed or silenced using lentiviral transfection, and cells were grouped into oe-NC, oe-DNMT1, sh-NC, and sh-DNMT1. The transfection efficiency was verified by RT-qPCR and Western blot. Results confirmed that DNMT1 overexpression significantly increased both mRNA and protein levels, whereas silencing resulted in a marked decrease. The most effective silencing construct was selected for subsequent experiments (Fig. S3A–D).

**Fig. 4:**
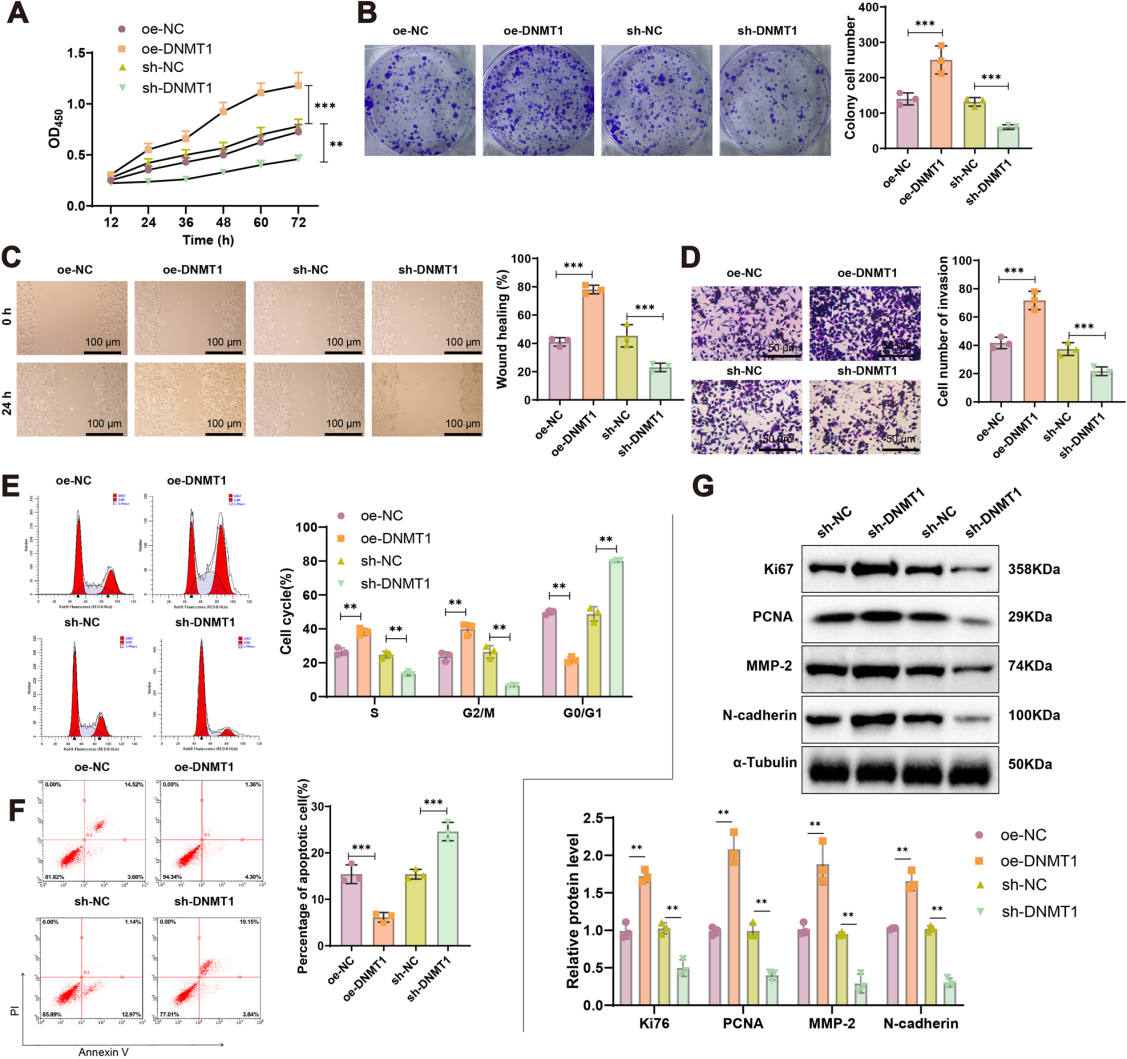
Influence of DNMT1 on the biological functions of breast cancer cells. **A** CCK-8 assay to measure the viability of 4T1 cells in each group. **B** Colony formation experiment to assess the growth and proliferation capacity of 4T1 cells in each group. **C** Wound healing assay to evaluate the migration ability of 4T1 cells in each group (scale bar: 100 μm). **D** Transwell assay to examine the invasion ability of 4T1 cells in each group (scale bar: 50 μm). **E** Flow cytometry analysis of cell cycle changes in 4T1 cells in each group. **F** Flow cytometry analysis of apoptosis in 4T1 cells in each group. **G** Western blot analysis to detect the expression of Ki67, PCNA, MMP-2, and N-cadherin proteins in cells from each group. All cell experiments were conducted in triplicate; ** at *P* < 0.01 and *** at *P* < 0.001 when comparing between groups.

Functional assays—including CCK-8, colony formation, wound healing, and Transwell invasion assays—demonstrated that DNMT1 overexpression significantly enhanced cell viability, proliferation, migration, and invasion compared to the oe-NC group. Conversely, DNMT1 knockdown in the sh-DNMT1 group significantly suppressed these cellular behaviors relative to the sh-NC group (Fig. 4A–D).

Cell cycle analysis using flow cytometry showed that the proportion of cells in the S and G2/M phases was significantly higher in the oe-DNMT1 group and lower in the sh-DNMT1 group, compared to their respective controls. Correspondingly, the G0/G1 phase cell population decreased in the oe-DNMT1 group and increased in the sh-DNMT1 group (Fig. 4E).

Apoptosis analysis revealed that compared to the oe-NC group, apoptosis was significantly reduced in the oe-DNMT1 group. In contrast, compared to the sh-NC group, apoptosis was significantly increased in the sh-DNMT1 group (Fig. 4F). Western blot analysis demonstrated that, compared to the oe-NC group, there was a significant increase in the expression of proliferative markers Ki67 and PCNA, as well as migration and invasion proteins MMP-2 and N-cadherin in the oe-DNMT1 group. Conversely, compared to the sh-NC group, the expression of proliferation, migration, and invasion-related proteins was significantly reduced in the sh-DNMT1 group (Fig. 4G). These findings further confirm that DNMT1 can enhance the proliferation, colony formation, migration, and invasion capabilities of breast cancer cells.

In summary, the outcomes indicate that DNMT1 can promote the proliferation, colony formation, migration, and invasion abilities of breast cancer cells.

### DNMT1 facilitates brain metastasis of breast cancer in vivo

**Fig. 5:**
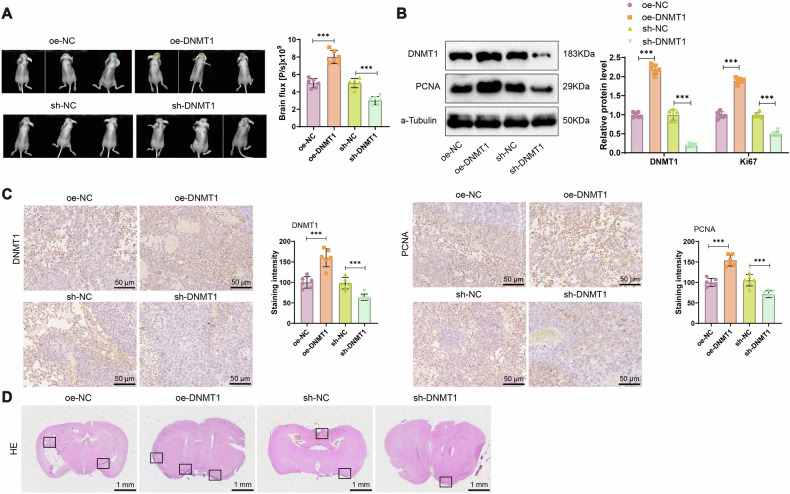
Impact of DNMT1 on brain metastases in breast cancer in mice. **A** Tumor growth measurement in mice groups using the IVIS Lumina II imaging system. **B** Detection of DNMT1 and PCNA expression in mouse brain tissues by Western blotting. **C** Immunohistochemical analysis of positive expression of DNMT1 and PCNA in mouse brain tissues (scale bar=50 μm). **D** H&E staining to examine brain metastasis nodules in mice groups (scale bar=50 μm), with black boxes highlighting the brain metastasis nodules. Each group consists of 6 mice; *** indicates statistical significance at *P* < 0.001 when comparing between groups.

To further investigate the role of DNMT1 in promoting breast cancer brain metastasis, an in vivo metastasis model was established by injecting HER2^+^/EGFR^+^/HPSE^+^/Notch1^+^ CTCs into the left ventricle of female BALB/c nude mice. Metastatic progression was monitored using the IVIS Lumina II imaging system. The results revealed a significant increase in brain metastatic burden in the oe-DNMT1 group compared to the oe-NC group. Conversely, a marked reduction in brain metastases was observed in the sh-DNMT1 group relative to the sh-NC group (Fig. 5A). Immunoblotting and immunohistochemical analysis of brain tissues demonstrated significantly elevated expression of DNMT1 and the proliferation marker PCNA in the oe-DNMT1 group, whereas their expression was substantially reduced in the sh-DNMT1 group compared to respective controls (Fig. 5B, C). H&E staining further confirmed an increased number of metastatic nodules in the oe-DNMT1 group and a notable reduction in the sh-DNMT1 group (Fig. 5D).

These findings provide compelling in vivo evidence that DNMT1 promotes brain metastasis in breast cancer.

### RASSF1A acts as a key methylation-regulated gene in brain metastatic breast cancer

While the role of DNMT1 in breast cancer brain metastasis has been established, its underlying molecular mechanism remains to be fully elucidated. Given DNMT1’s primary function as a DNA methyltransferase, we hypothesized that it may mediate its pro-metastatic effects through the methylation of key regulatory genes. To explore this, a genome-wide methylation sequencing analysis was performed on breast cancer cells, allowing for the identification of genes whose expression may be epigenetically regulated by DNMT1. This approach not only revealed candidate genes directly influenced by DNMT1-mediated methylation but also provided broader insights into DNA methylation events associated with breast cancer brain metastases.

**Fig. 6:**
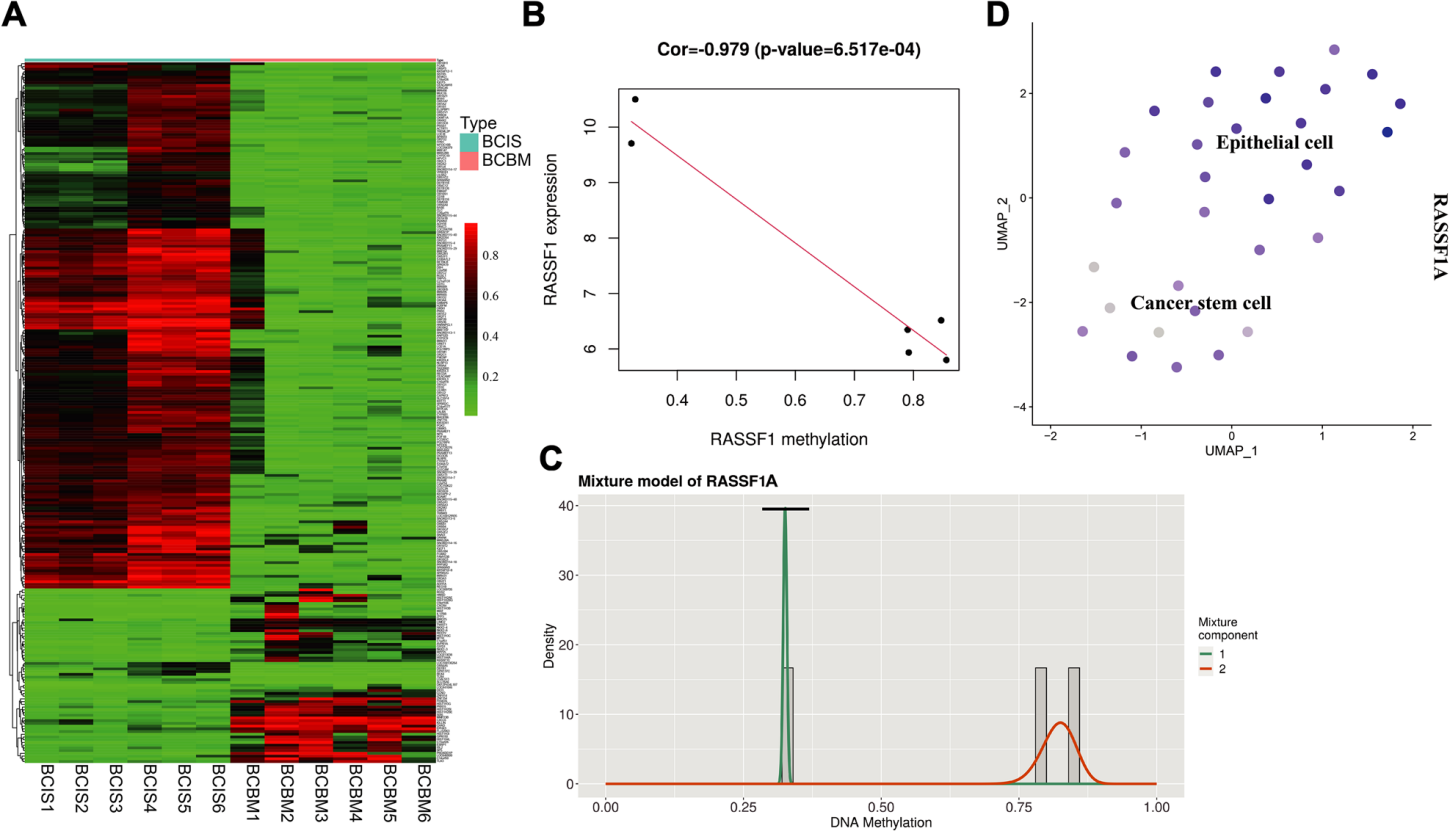
Selection of DNA methylation-driven genes through transcriptome combined with DNA methylation data. **A** Heatmap of Methylation differentially expressed genes selected from DNA methylation data (BCIS group, *n* = 6; BCBM group, *n* = 6). **B** Correlation between RASSF1 Methylation levels and mRNA levels. **C** Methylation status of RASSF1 in Brain metastases samples compared to breast cancer in situ samples. **D** Expression pattern of RASSF1 in various cell subgroups, with deeper blue indicating higher average expression levels. **P < 0.01 and ***P < 0.001 when comparing between groups.

Differential methylation analysis between in situ breast cancer and brain metastasis samples identified 43 differentially methylated genes (Fig. 6A). Integration of these data with transcriptomic profiles using the MethylMix package and a Beta mixture model pinpointed RASSF1A as a potential methylation-driven gene. RASSF1A showed significantly higher methylation levels and reduced mRNA expression in brain metastasis samples compared to in situ tumors, indicating a strong inverse correlation (Fig. 6B, C). Additionally, RASSF1A expression was notably lower in CSCs (Fig. 6D).

Overall, these findings suggest that RASSF1A is a key methylation-regulated gene potentially silenced by DNMT1, contributing to the metastatic progression of breast cancer to the brain.

### DNMT1 epigenetically regulates RASSF1A via DNA methylation

**Fig. 7:**
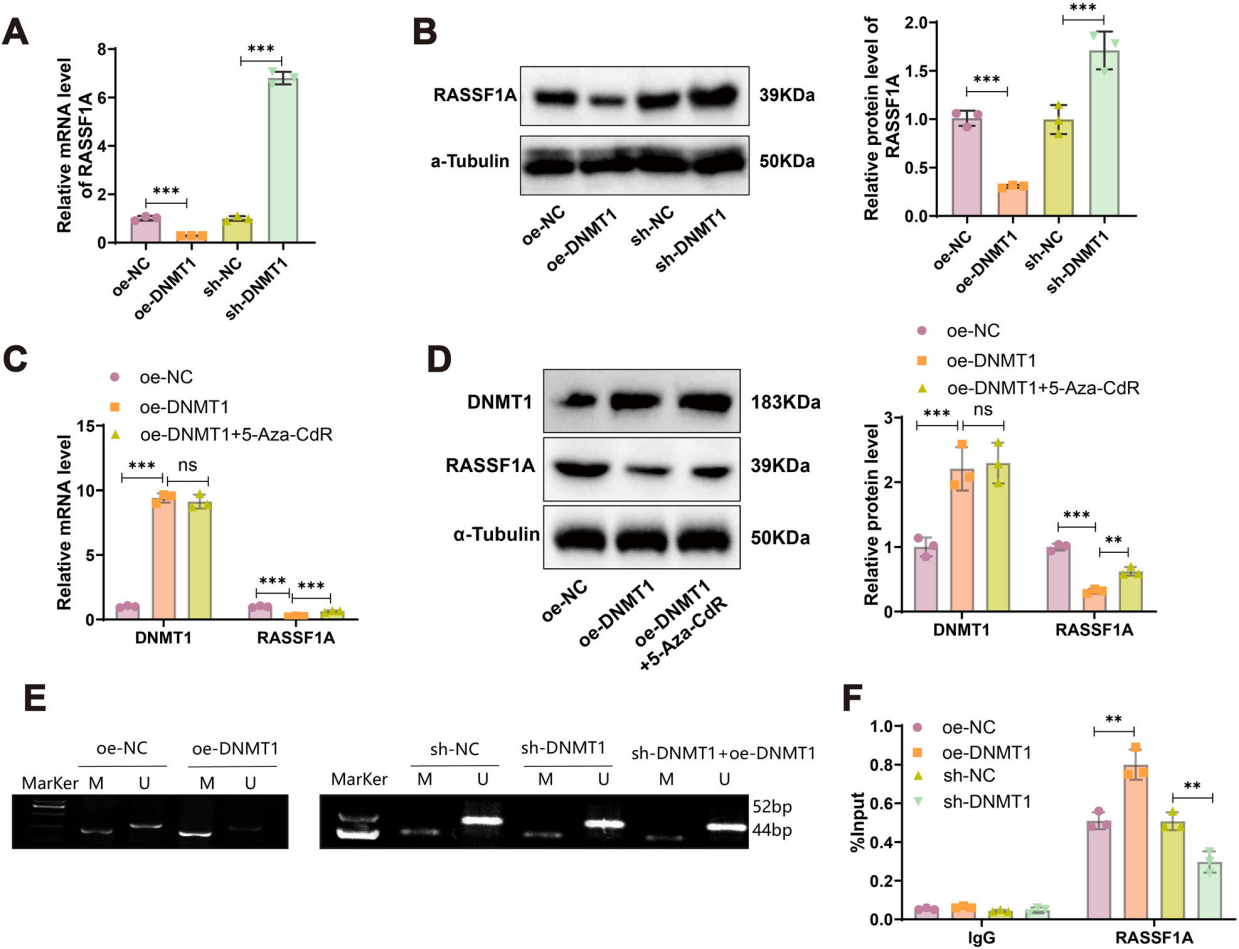
Impact of DNMT1 on RASSF1A Methylation. **A**, **B** RT-PCR and Western blot to assess the effect of DNMT1 overexpression or knockdown on RASSF1A mRNA and protein levels in 4T1 cells. **C**, **D** RT-PCR and Western blot to examine the impact of 5-Aza-CdR on DNMT1 and RASSF1A mRNA and protein levels in 4T1 cells. **E** MSP to detect the Methylation status of RASSF1A in each group (M Methylation-specific primers for MSP, U MSP using non-Methylation-specific primers; bands at 52 bp and 44 bp indicate the presence of unmethylated and methylated sequences). **F** ChIP experiment to assess DNMT1 enrichment on the RASSF1A promoter. All cell experiments were repeated thrice; ns denotes no significant difference between groups, ** indicates statistical significance at *P* < 0.01, and *** at *P* < 0.001.

Based on methylation sequencing data, *RASSF1A* was identified as a critical methylation-driven gene. To investigate whether DNMT1 regulates *RASSF1A* methylation, we manipulated DNMT1 expression in 4T1 cells. Knockdown of DNMT1 significantly increased both mRNA and protein levels of *RASSF1A*, whereas DNMT1 overexpression led to a notable reduction in its expression (Fig. 7A, B). Furthermore, treatment with the DNA methylation inhibitor 5-Aza-CdR reversed the DNMT1-induced repression of *RASSF1A* at both transcript and protein levels (Fig. 7C, D). MSP analysis revealed that DNMT1 overexpression increased the methylation level of the *RASSF1A* promoter, while DNMT1 knockdown reduced promoter methylation. To exclude the possibility of shRNA off-target effects, a rescue experiment was conducted by reintroducing the DNMT1 overexpression construct into DNMT1-silenced cells, which partially restored the methylation level (Fig. 7E). Additionally, ChIP-qPCR analysis demonstrated increased DNMT1 enrichment at the *RASSF1A* promoter upon DNMT1 overexpression, and reduced binding following DNMT1 knockdown (Fig. 7F). Collectively, these results indicate that DNMT1 directly modulates *RASSF1A* promoter methylation, thereby regulating its expression.

### Prognostic association of DNMT1 and RASSF1 expression levels in breast cancer patients

To further evaluate the clinical significance of DNMT1 and RASSF1A in breast cancer patients, we performed survival analyses using transcriptomic data from the TCGA-BRCA cohort. Because the public database only provides overall RASSF1 gene expression without distinguishing between isoforms (such as RASSF1A and RASSF1C), we used RASSF1 gene-level expression as a surrogate marker. Kaplan–Meier curve analysis showed that neither DNMT1 nor RASSF1 high expression was significantly associated with OS or disease-specific survival in breast cancer patients (log-rank *P* > 0.05) (Fig. S4A–D). These results suggest that neither serves as an independent prognostic indicator in the overall breast cancer population.

It is noteworthy, however, that existing public databases do not provide isoform-specific expression data for RASSF1A, the tumor-suppressive isoform shown in this study to exert important biological functions and whose expression is frequently regulated by promoter hypermethylation. Thus, the current analysis may underestimate its true prognostic value. Our subsequent functional and methylation-based mechanistic experiments confirmed that DNMT1 promotes breast cancer brain metastasis through the regulation of RASSF1A methylation, thereby complementing the limitations of the database analysis.

### DNMT1 knockdown suppresses malignant behaviors via RASSF1A Demethylation

To further validate whether DNMT1 promotes the malignant behavior of breast cancer cells through *RASSF1A* methylation, we silenced *RASSF1A* in 4T1 cells. RT-qPCR and Western blot analysis confirmed the effective knockdown of *RASSF1A*, with the second shRNA construct demonstrating superior efficiency and selected for further experiments (Fig. S5A, B).

**Fig. 8:**
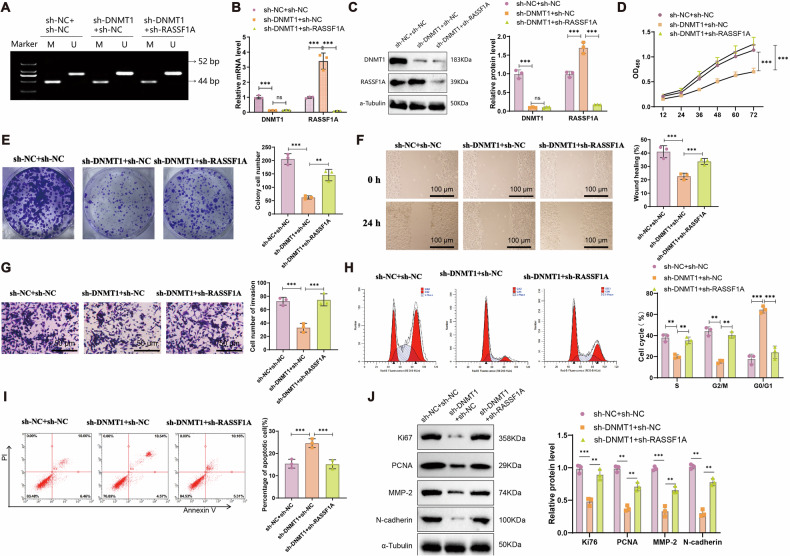
Effect of DNMT1 on breast cancer cell biological functions through modulating RASSF1A. **A** MSP to evaluate the Methylation status of RASSF1A in each group (M Methylation-specific primers for MSP, U MSP using non-methylation-specific primers; bands at 52 bp and 44 bp indicate the presence of unmethylated and methylated sequences). **B**, **C** RT-qPCR and Western blot to analyze DNMT1 and RASSF1A mRNA and protein expression levels in 4T1 cells of each group. **D** CCK-8 assay for assessing the cell viability of 4T1 cells in each group. **E** Colony formation experiment to evaluate the growth and proliferation capacity of 4T1 cells in each group. **F** Wound healing experiment to measure the migration ability of 4T1 cells (scale bar: 100 μm). **G** Transwell assay to assess the invasion ability of 4T1 cells in each group (scale bar: 50 μm). **H** Flow cytometry analysis of cell cycle changes in 4T1 cells. **I** Flow cytometry analysis of apoptosis in 4T1 cells of each group. **J** Western blot analysis of proliferation- and metastasis-related protein expression. All cell experiments were repeated thrice; ** at *P* < 0.01 and *** at *P* < 0.001.

Co-silencing experiments were then performed using both DNMT1 and *RASSF1A* shRNAs. MSP analysis showed that DNMT1 knockdown significantly reduced *RASSF1A* promoter methylation, while *RASSF1A* silencing did not influence its own methylation status (Fig. 8A). RT-qPCR and Western blot results indicated that the sh-DNMT1+sh-NC group exhibited reduced DNMT1 expression and elevated *RASSF1A* levels compared to the sh-NC+sh-NC control. However, in the sh-DNMT1+sh-RASSF1A group, *RASSF1A* expression was significantly decreased, while DNMT1 levels remained unchanged (Fig. 8B, C).

Functional assays revealed that DNMT1 knockdown suppressed cell viability, colony formation, migration, and invasion, while *RASSF1A* knockdown reversed these suppressive effects. Specifically, compared to the sh-NC+sh-NC group, the sh-DNMT1+sh-NC group showed significant inhibition of malignant phenotypes, whereas the sh-DNMT1+sh-RASSF1A group exhibited a clear restoration of these phenotypes (Fig. 8D–G). Flow cytometry analysis of the cell cycle revealed that compared to the sh-NC+sh-NC group, the sh-DNMT1+sh-NC group had a significantly reduced proportion of S + G2/M phase cells and an increased proportion of G0/G1 phase cells. Conversely, compared to the sh-DNMT1+sh-NC group, the sh-DNMT1+sh-RASSF1A group showed a significant increase in S + G2/M phase cells and a decrease in G0/G1 phase cells (Fig. 8H). Additionally, apoptosis analysis through flow cytometry revealed a significant increase in cell apoptosis in the sh-DNMT1+sh-NC group compared to the sh-NC+sh-NC group. Conversely, the sh-DNMT1+sh-RASSF1A group exhibited a significant decrease in cell apoptosis compared to the sh-DNMT1+sh-NC group (Fig. 8I). Western blot analysis showed that compared to the sh-NC+sh-NC group, the sh-DNMT1+sh-NC group displayed a significant decrease in the expression of Ki67, PCNA, MMP-2, and N-cadherin markers. In contrast, the sh-DNMT1+sh-RASSF1A group exhibited a significant increase in the expression of markers associated with cell proliferation, migration, and invasion compared to the sh-DNMT1+sh-NC group (Fig. 8J).

In summary, these findings suggest that DNMT1 promotes breast cancer cell proliferation, migration, and invasion by methylating and silencing *RASSF1A*.

### DNMT1 downregulation inhibits brain metastasis through *RASSF1A* Demethylation

**Fig. 9:**
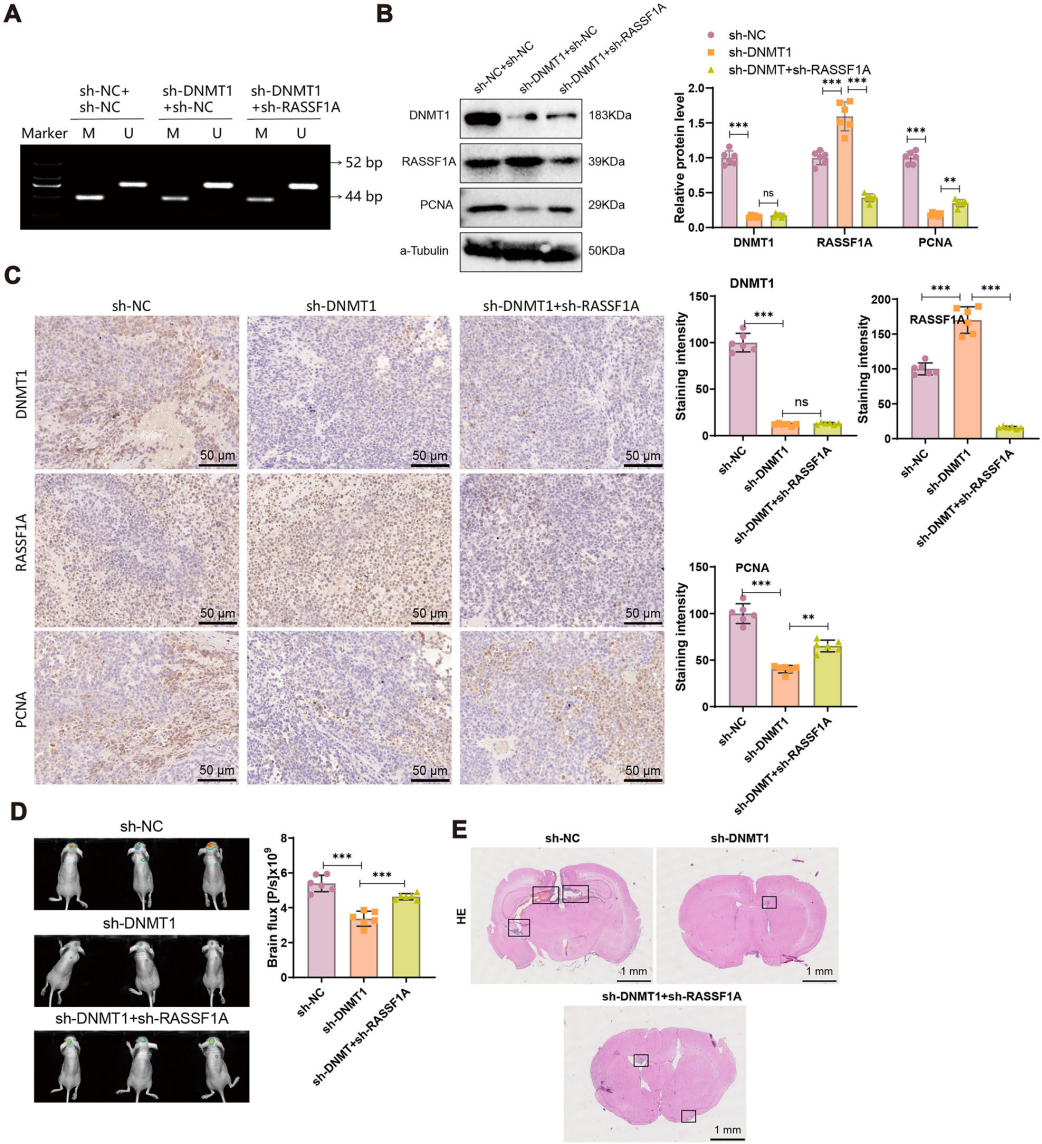
Impact of DNMT1 on brain metastases in breast cancer in mice. **A** MSP analysis to determine the Methylation status of RASSF1A in each group (M Methylation-specific primers for MSP, U Non-Methylation-specific primers for MSP; detection of bands at 52 bp and 44 bp signifies the presence of unmethylated and methylated sequences). **B** Western blot assessment of DNMT1, RASSF1A, and PCNA expression in mouse brain tissues for all groups. **C** Immunohistochemical evaluation of positive expression of DNMT1, RASSF1A, and PCNA in mouse brain tissues (scale bar=50 μm). **D** Tumor growth measurement in mice groups using the IVIS Lumina II imaging system. **E** H&E staining to examine brain metastasis nodules in mouse groups (scale bar = 50 μm), with black boxes indicating the brain metastasis nodules. Each group comprised 6 mice; ns indicates no significant difference between groups, and *** signifies statistical significance at *P* < 0.001 when comparing groups.

To further investigate whether DNMT1 promotes breast cancer brain metastasis through *RASSF1A* methylation, we utilized a CTC-derived breast cancer brain metastasis mouse model. MSP analysis demonstrated that DNMT1 knockdown significantly reduced methylation of the *RASSF1A* promoter, whereas silencing *RASSF1A* alone had no significant effect on its promoter methylation (Fig. 9A). Western blot and immunohistochemistry analysis of brain tissues revealed that, compared to the sh-NC+sh-NC group, the sh-DNMT1+sh-NC group exhibited markedly decreased expression of DNMT1 and the proliferation marker PCNA, along with significantly increased *RASSF1A* expression. In contrast, the sh-DNMT1+sh-RASSF1A group showed no significant change in DNMT1 expression but demonstrated a significant reduction in *RASSF1A* expression and a corresponding increase in PCNA levels compared to the sh-DNMT1+sh-NC group (Fig. 9B, C).

Metastatic progression was further assessed using the IVIS Lumina II in vivo imaging system. The sh-DNMT1+sh-NC group exhibited a significant reduction in brain metastases relative to the sh-NC+sh-NC control group. Conversely, the sh-DNMT1+sh-RASSF1A group showed a notable increase in metastatic burden compared to the sh-DNMT1+sh-NC group (Fig. 9D). H&E staining of brain sections corroborated these findings, showing fewer metastatic nodules in the sh-DNMT1+sh-NC group compared to the control, and a pronounced increase in metastatic foci in the sh-DNMT1+sh-RASSF1A group (Fig. 9E).

Taken together, the results suggest that DNMT1 downregulation suppresses brain metastasis of breast cancer by reducing *RASSF1A* promoter methylation and restoring its expression, thereby impairing the metastatic potential of breast cancer cells.

## Discussion

In our study, we demonstrated that DNMT1 overexpression played a critical role in the development and progression of breast cancer brain metastases, primarily through the epigenetic silencing of *RASSF1A* via DNA methylation. This novel mechanistic insight fills an important gap in the current literature. Previous studies have associated DNMT1 overexpression with tumorigenesis and metastasis in various cancers [18, 69], and *RASSF1A* promoter hypermethylation has been implicated in its transcriptional silencing across multiple tumor types [21, 70]. However, these studies largely examined DNMT1 and *RASSF1A* as independent factors, without exploring a direct regulatory relationship between them. In contrast, our findings provide evidence of a direct functional interaction between DNMT1 and *RASSF1A* in the context of breast cancer brain metastasis, thereby expanding upon prior studies [71, 72]. Specifically, we showed that DNMT1 promoted metastatic behavior by enhancing methylation of the *RASSF1A* promoter, leading to its silencing. This regulatory axis is shown to be functionally relevant both in vitro and in vivo, as DNMT1 downregulation restores *RASSF1A* expression and impairs metastatic progression.

Compared to existing literature, our work offers two key contributions: (1) it underscores the importance of DNMT1 as a driver of breast cancer brain metastases, and (2) it identifies *RASSF1A* as a direct downstream effector of DNMT1-mediated DNA methylation [73, 74]. These findings advance our understanding of the molecular mechanisms underlying breast cancer brain metastasis and propose DNMT1 and *RASSF1A* as potential therapeutic targets. Moreover, our results highlight the broader significance of epigenetic regulation in tumor metastasis. The DNMT1-*RASSF1A* axis exemplifies how aberrant DNA methylation can disrupt tumor suppressor gene expression, facilitating metastatic dissemination. This mechanistic insight into epigenetic modulation deepens our understanding of the regulatory networks that govern breast cancer brain metastasis and may inform the design of targeted epigenetic therapies. In conclusion, our study provides compelling evidence that DNMT1 promotes brain metastasis in breast cancer by regulating the methylation status of *RASSF1A*. This represents a previously underexplored mechanism with important implications for the development of novel therapeutic strategies targeting epigenetic regulators in metastatic breast cancer.

In this study, we successfully isolated CTCs from an in situ breast cancer mouse model using microfluidic technology and subsequently performed scRNA-seq analysis. This approach offers a novel and powerful perspective for elucidating intra-tumoral heterogeneity and the molecular mechanisms underlying brain metastasis, highlighting the significant potential of microfluidic platforms in future cancer research. Our findings demonstrate that DNMT1 promotes breast cancer brain metastasis by regulating *RASSF1A* DNA methylation, providing a novel strategic direction for therapeutic intervention. Targeting DNMT1 or *RASSF1A*—through small molecule inhibitors, epigenetic modulators, or gene therapy—may offer more precise and effective treatment options for patients with breast cancer.

**Fig. 10:**
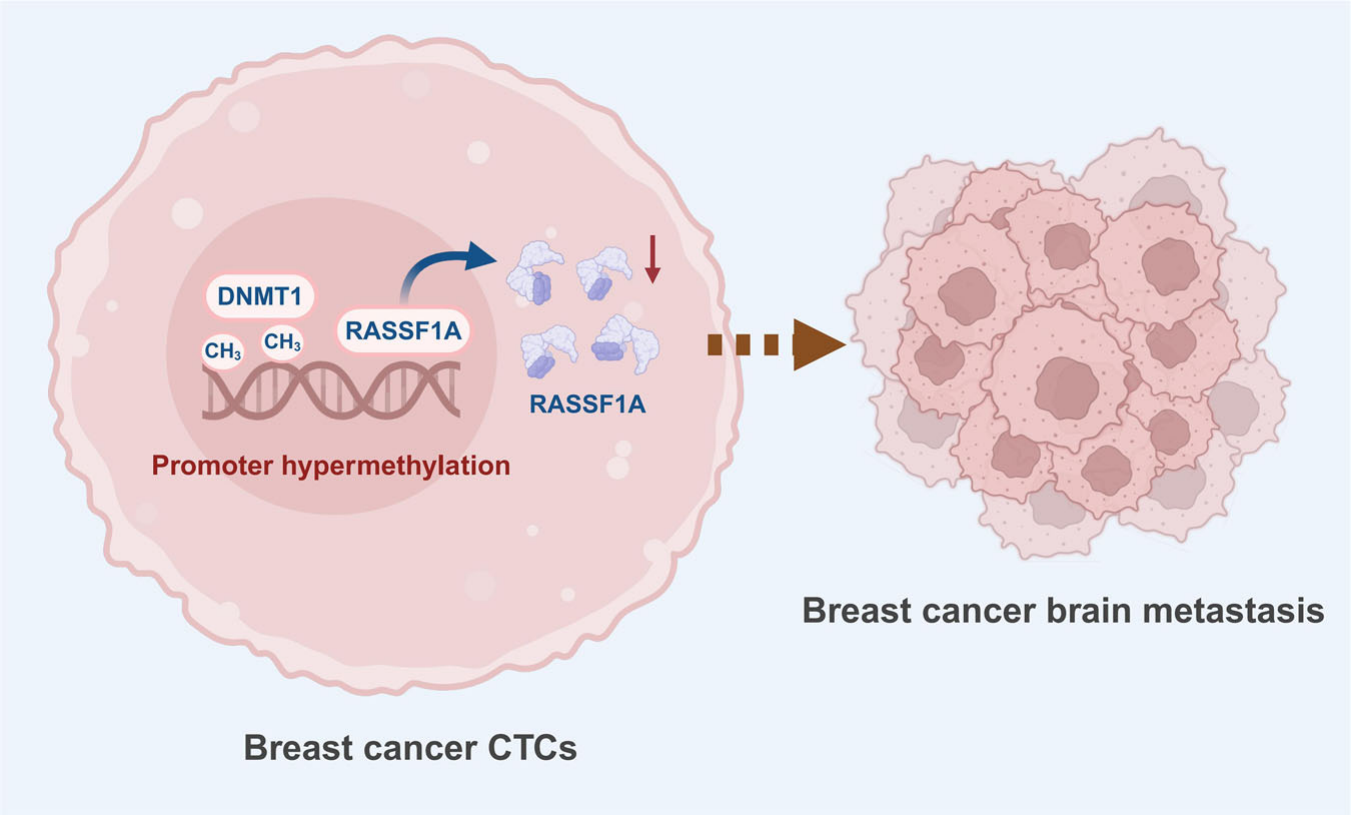
Schematic representation of the molecular mechanism of DNMT1-driven RASSF1A DNAMethylation in CTCs contributing to early breast cancer metastasis (Created with BioRender.com).

In summary, our study demonstrates that DNMT1-driven RASSF1A DNA methylation in CTCs plays a pivotal role in early brain metastasis of breast cancer (Fig. 10). This finding provides new insights into the pathogenesis of breast cancer brain metastasis and highlights the DNMT1–RASSF1A axis as a potential therapeutic target for more precise treatment strategies. Notably, silencing of RASSF1A is an important contributor but not the sole driver of metastasis. Previous studies have reported that DNMT1 cooperates with PAS1 and PH20 to promote breast cancer growth and metastasis [74], and DNMT1-mediated hypermethylation of the FOXO3a promoter downregulates its expression, enhancing stemness and accelerating tumor progression through inhibition of the FOXM1/SOX2 pathway [75]. These findings suggest that breast cancer metastasis may involve multiple DNMT1 substrates acting in concert. Incorporating microfluidic technology and single-cell sequencing, our study also provides valuable insights into intratumoral heterogeneity, supporting the development of individualized therapeutic approaches.

Nevertheless, several limitations remain. First, this study did not include breast cancer brain metastasis patient samples with detailed clinical outcomes such as OS or metastasis-free survival, limiting direct prognostic evaluation of DNMT1 and RASSF1A. Analysis of the TCGA-BRCA cohort showed no significant association between DNMT1 or RASSF1 expression and OS or disease-specific survival, suggesting they may not serve as independent prognostic markers in the general population. However, these results should be interpreted with caution: (i) public databases lack isoform-specific data for RASSF1A, the tumor-suppressive isoform confirmed in this study; and (ii) TCGA does not provide brain metastasis-specific clinical information, which may obscure survival differences due to population heterogeneity. Together with our experimental data, these findings indicate that the DNMT1–RASSF1A axis may play a more critical role in specific subpopulations, such as stem-like CTCs with high brain metastatic potential. Future studies incorporating breast cancer brain metastasis cohorts with comprehensive follow-up data are warranted to validate the association of DNMT1/RASSF1A expression and methylation status with patient outcomes, thereby strengthening the translational relevance of our findings.

Second, while murine models provide valuable insight into human cancer biology, species-specific differences in physiology, anatomy, and genetics may limit extrapolation. Although NSG mice, due to their immunodeficient background, allow for the study of tumor progression without immune interference [76], they are inadequate for assessing the influence of the tumor microenvironment on metastatic processes. Therefore, validation in human tissue samples remains essential.

Additionally, while single-cell sequencing enables detailed analysis of intratumoral heterogeneity, it is inherently prone to technical noise and complexity. Although we employed multiple in vitro and in vivo validation methods, further confirmatory approaches are required to ensure the robustness and generalizability of our results. Moreover, although 43 differentially methylated genes were identified, our study focused exclusively on *RASSF1A*. Future investigations should explore potential cooperative effects with other genes or signaling pathways that may be co-regulated by DNMT1. A broader understanding of the DNMT1-regulated epigenetic landscape could reveal synergistic mechanisms contributing to metastasis. If the molecular findings presented here are validated in preclinical models, subsequent clinical trials could evaluate the therapeutic efficacy of DNMT1-targeted interventions in breast cancer. In this study, NSG and BALB/c mice were used to establish breast cancer brain metastasis models derived from CTCs, balancing efficient model construction with partial representation of immune contributions. Stable 4T1-BM cells obtained from NSG mice were subsequently implanted into immunocompetent BALB/c mice, enabling metastasis validation within a more intact immune microenvironment. The immune system is crucial in brain metastasis, where the immune-privileged status of the brain further shapes the metastatic niche. However, our models cannot fully reproduce the complex immune responses of fully immunocompetent hosts, which is a limitation. Future studies should employ spontaneous brain metastasis models in immunocompetent mice and consider syngeneic or humanized models to better elucidate the role of the DNMT1/RASSF1A axis and immune cells in this process.

Furthermore, expanding this line of research to other cancer types may help determine whether DNMT1 exerts similar regulatory roles in promoting metastasis and whether *RASSF1A* methylation status serves as a generalizable biomarker. Lastly, it is also important to acknowledge the context-dependent nature of DNMT1 in cancer biology. While DNMT1 often functions as an oncogene by maintaining aberrant methylation patterns, it may also act as a tumor suppressor in certain therapeutic contexts. For instance, in oral squamous cell carcinoma, DNMT1 knockdown induces global hypomethylation, inactivating PI3K-AKT and CDK2-Rb signaling, and cooperating with GSK3β inactivation to suppress tumorigenicity [77]. Conversely, Conversely, in BRCA-mutant cancers, DNMT1 promotes resistance to PARP inhibitors (PARPi) by modulating DNA repair processes such as RPA complex recruitment and replication fork stability; thus, DNMT1 inhibition sensitizes tumors to PARPi [78].

In summary, our findings identify DNMT1 as a key epigenetic regulator that facilitates breast cancer brain metastasis by repressing *RASSF1A* and modulating downstream signaling pathways. Functional assays confirmed DNMT1’s role in promoting malignant phenotypes, including proliferation, migration, invasion, and brain colonization. Integrated transcriptomic and methylation analyses further underscored the pivotal contribution of the DNMT1–*RASSF1A* axis in gene regulation and tumor progression. Future studies should investigate upstream regulators of DNMT1 in the metastatic cascade and evaluate the therapeutic potential of targeting the DNMT1–*RASSF1A* axis using in vivo models. Validation in patient-derived xenograft models and clinical cohorts will be critical for confirming the translational relevance of this pathway and supporting the development of epigenetic therapies for breast cancer brain metastasis.

## Supplementary information

Supplementary material

western blots

## Data Availability

The datasets generated and/or analyzed during the current study are not publicly available due to privacy and confidentiality agreements with the participants, but are available from the corresponding author on reasonable request.
