# Supplementary material for: DNMT1 modulation of RASSF1A methylation enhances breast cancer brain metastasis
Source: Cell Death Dis. 2025 Dec 11;17(1):80. doi: 10.1038/s41419-025-08167-x (PMC12830775; doi:10.1038/s41419-025-08167-x)
Supplement: Supplementary file 1 — Supplementary material [file 41419_2025_8167_MOESM1_ESM.docx]

**
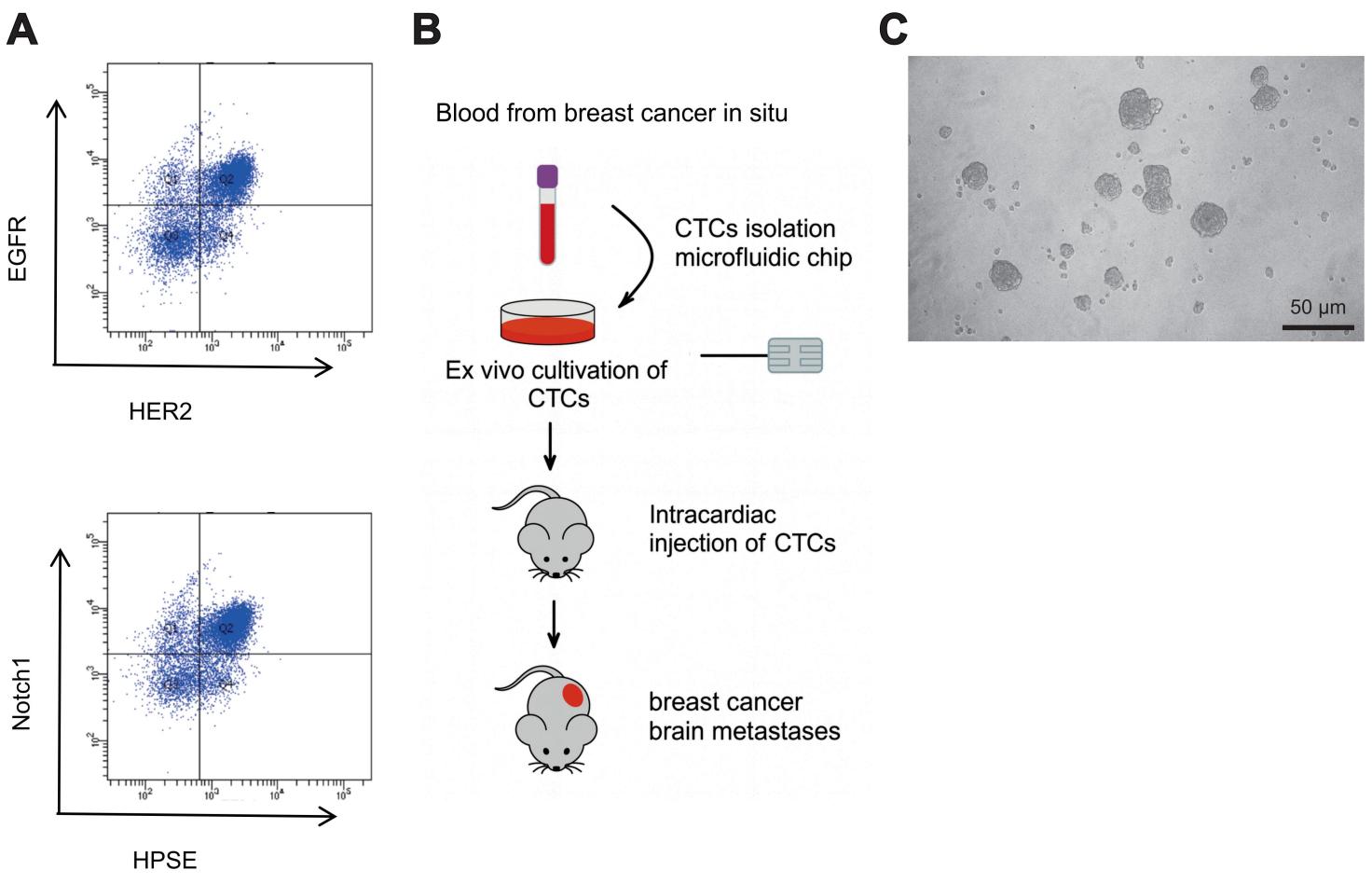
**

**Fig. S1 Establishment of CTCs Brain metastasis mouse model.**

Note: (A) Flow cytometry sorting of HER2^+^/EGFR^+^/HPSE^+^/Notch1^+^ positive CTCs; (B) Workflow diagram for creating the CTCs Brain metastasis mouse model (Created with BioRender.com); Tumorsphere formation assay of HER2⁺/EGFR⁺/HPSE⁺/Notch1⁺ CTCs. Round structures were defined as tumorspheres.


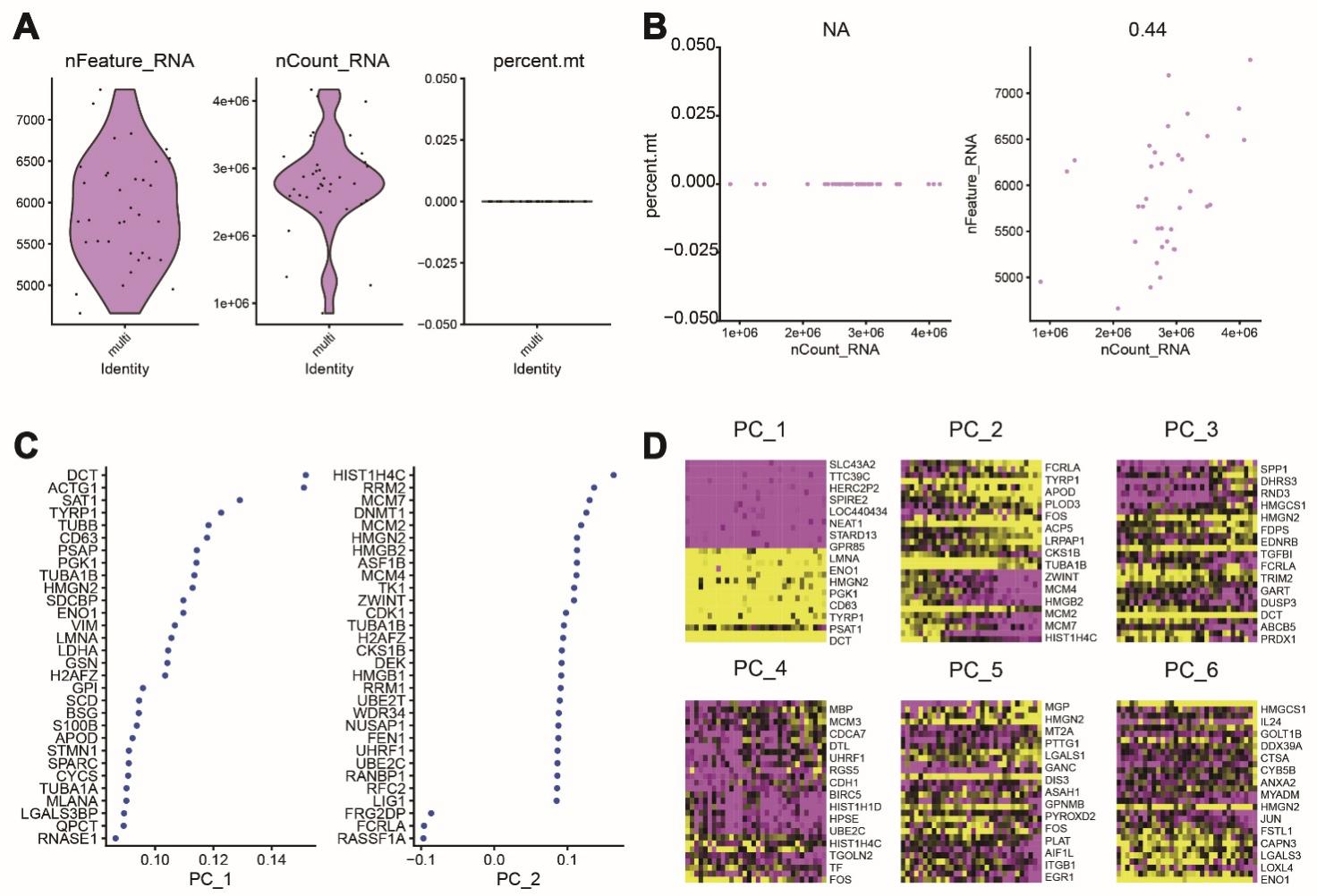


**Fig. S2 Quality control and PCA dimensionality reduction of scRNA-seq data.**

Note: (A) Violin plots displaying the gene count (nFeature_RNA), mRNA molecule count (nCount_RNA), and percentage of mitochondrial genes (percent. mt) for each cell in the scRNA-seq data; (B) Scatter plots showing the correlation between filtered data nCount_RNA and percent. Mt, and nCount_RNA and nFeature_RNA; (C) Feature genes in the top 2 PCs before PCA analysis; (D) Heatmap of top 15 significantly correlated genes in PC_1 – PC_6 in PCA, where yellow denotes upregulation and purple indicates downregulation.


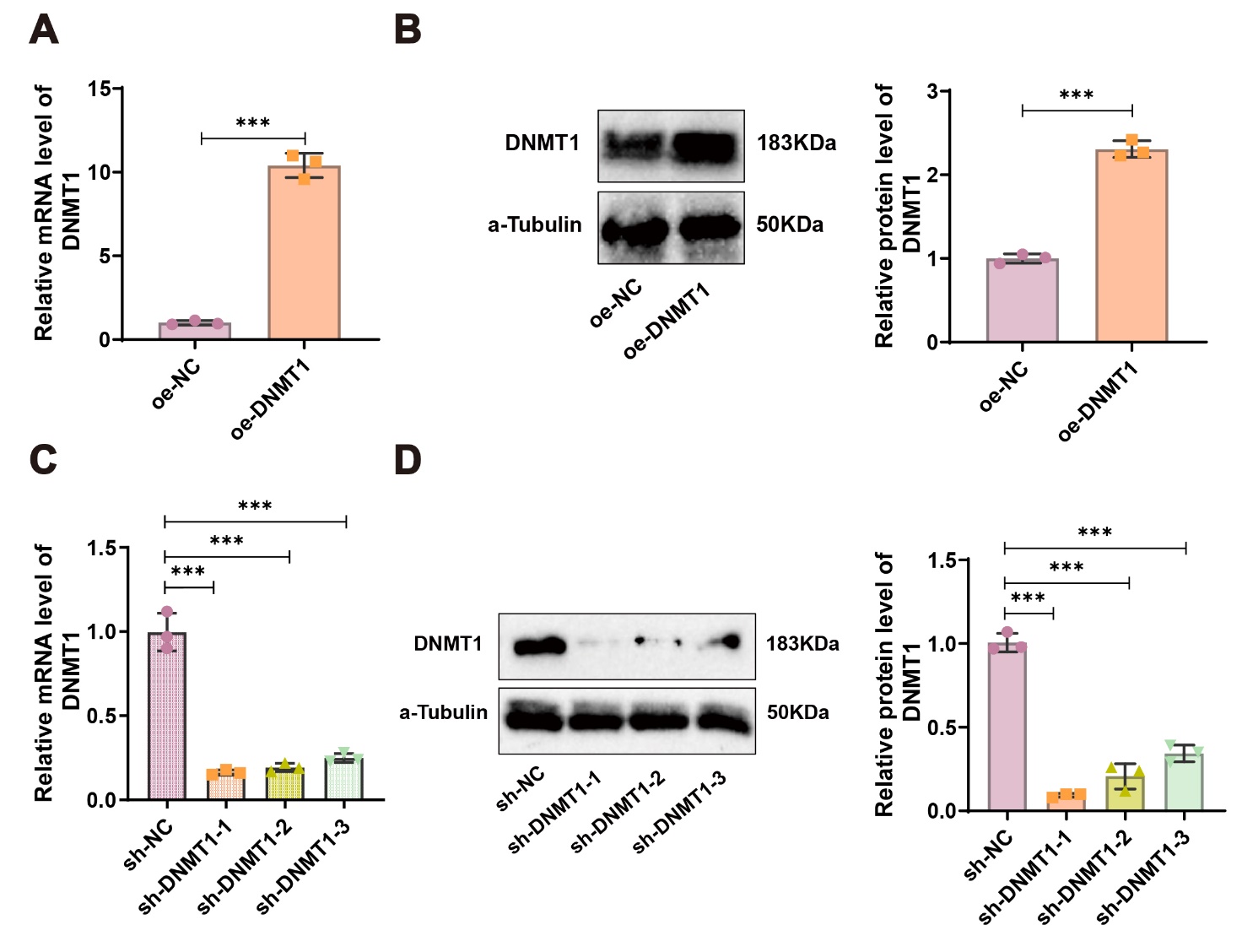


**Fig. S3 Verification of DNMT1 knockout and overexpression efficiency.**

Note: (A-B) RT-PCR and Western blot analysis to assess the overexpression efficiency of DNMT1 in 4T1 cells; (C-D) RT-PCR and Western blot analysis to evaluate the knockout efficiency of DNMT1 in 4T1 cells. All cell experiments were conducted in triplicate; *** denotes statistical significance at *P* < 0.001 when comparing between groups.


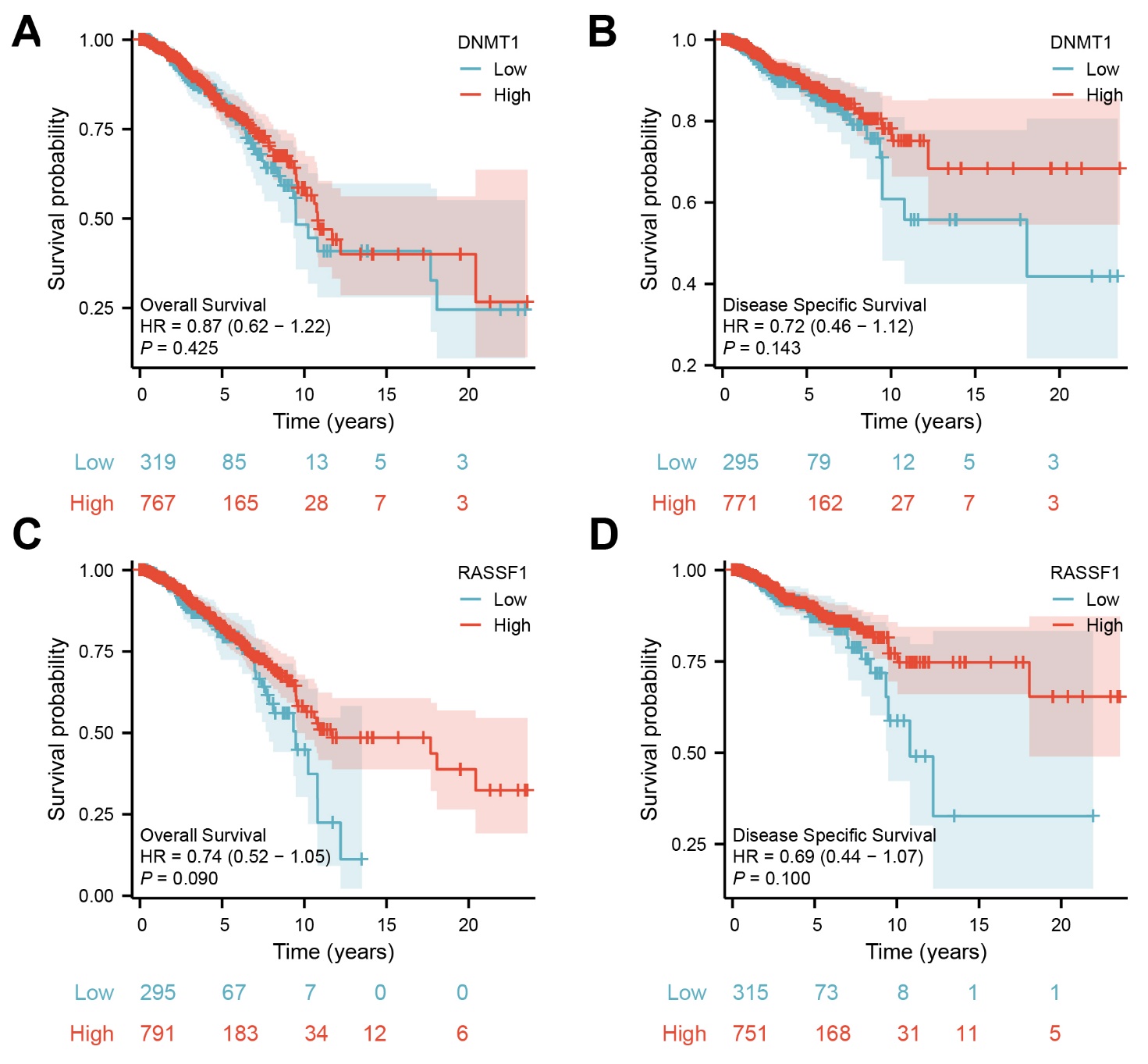


**Fig. S4 Effect of DNMT1 and RASSF1 expression levels on survival in breast cancer patients**Note: (A-B) Kaplan–Meier curves showing no significant association between DNMT1 expression and OS or DSS in the TCGA-BRCA cohort; (C-D) Kaplan–Meier curves showing no significant association between RASSF1 expression and OS or DSS in the TCGA-BRCA cohort.


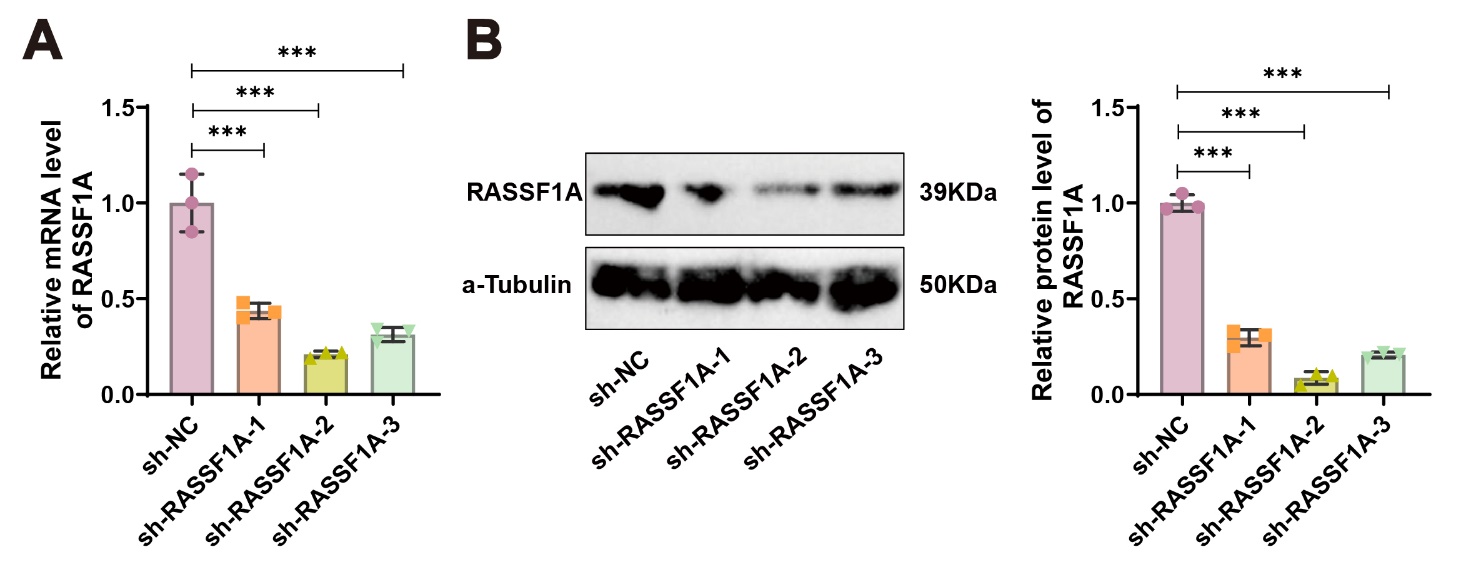


**Fig. S5 Verification of RASSF1A knockout and overexpression efficiency.**

Note: (A-B) RT-PCR and Western blot analysis to determine the knockout efficiency of RASSF1A in 4T1 cells. All cell experiments were repeated thrice; *** indicates statistical significance at *P* < 0.001 when comparing between groups.

**Table S1. shRNA Sequence Information**

| Name | shRNA sequence |
| --- | --- |
| shRNA-NC | Sense 5’-UAAGGCUAUGAAGAGAUAC-3’ |
|  | Antisense 5’-GUAUCUCUUCAUAGCCUUA-3’ |
| shRNA-DNMT1-1(human) | Sense 5’-GGAGTGCCCTGGCACAATTAT-3’ |
|  | Antisense 5’-ATAATTGTGCCAGGGCACTCC-3’ |
| shRNA-DNMT1-2(human) | Sense 5’-GAGTGCCCTGGCACAATTATA-3’ |
|  | Antisense 5’-TATAATTGTGCCAGGGCACTC-3’ |
| shRNA-DNMT1-3(human) | Sense 5’-AGTGCCCTGGCACAATTATAA-3’ |
|  | Antisense 5’-TTATAATTGTGCCAGGGCACT-3’ |
| shRNA-RASSF1A-1(human) | Sense 5’-CTTTAGGGTGAAGGGAAATTA-3’ |
|  | Antisense 5’-TAATTTCCCTTCACCCTAAAG-3’ |
| shRNA-RASSF1A-2(human) | Sense 5’-GACCCTAGGCATCAGTAAATT-3’ |
|  | Antisense 5’-AATTTACTGATGCCTAGGGTC-3’ |
| shRNA-RASSF1A-3(human) | Sense 5’-ACCCTAGGCATCAGTAAATTT-3’ |
|  | Antisense 5’-AAATTTACTGATGCCTAGGGT-3’ |

**Table S2. qRT-PCR Primer sequence**

| Gene | Primer Sequence (5'-3') |
| --- | --- |
| DNMT1 (human) | Forword: 5'-AGGAGGGCTACCTGGCTAAA-3'  Reverse: 5'-ACGGGCTTCACTTCTTGCTT-3' |
| RASSF1A (human) | Forword: 5'-CCTCTCTGCAGATTGCAAGTTC-3'  Reverse: 5'-AGGTCAGGTGTCTCCCACTC-3' |
| GAPDH (human) | Forword: 5'-AAAGCCTGCCGGTGACTAAC-3' |
|  | Reverse: 5'-TTCCCGTTCTCAGCCTTGAC-3' |

**Table S3. Western blot Antibody information**

| Target name | Manufacturer | Product number | Dilution ratio |
| --- | --- | --- | --- |
| DNMT1(rabbit) | Abcam | ab188453 | 1:1000 |
| RASSF1A(mouse) | Abcam | ab23950 | 1:1000 |
| PCNA(mouse) | Abcam | ab29 | 1:1000 |
| Ki67(rabbit) | Abcam | ab16667 | 1:1000 |
| MMP-2(rabbit) | Abcam | ab92536 | 1:1000 |
| N-cadherin(rabbit) | Abcam | ab76011 | 1:5000 |
| α-Tubulin(rabbit) | Abcam | ab7291 | 1:1000 |

**Table S4. MSP Primer sequence**

| Gene name | Primer sequence |
| --- | --- |
| RASSF1A (human)Non-methylated primer | Forward: 5’-GGGTTAGAGATTCGTTCGGA-3’ |
|  | Reverse: 5’-CTATCACATTCGAAAACGACG-3’ |
| RASSF1A (human)Non-methylated primer | Forward: 5’-GGGTTAGAGATTTGTTTGGA-3’ |
|  | Reverse:5’-AAATACTATCACATTCAAAAACAACAAAC-3’ |
