# Supplementary material for: DNMT1 modulation of RASSF1A methylation enhances breast cancer brain metastasis
Source: Cell Death Dis. 2025 Dec 11;17(1):80. doi: 10.1038/s41419-025-08167-x (PMC12830775; doi:10.1038/s41419-025-08167-x)

Full and uncropped western blots

## Full and uncropped western blots of figure 4G-1.jpg


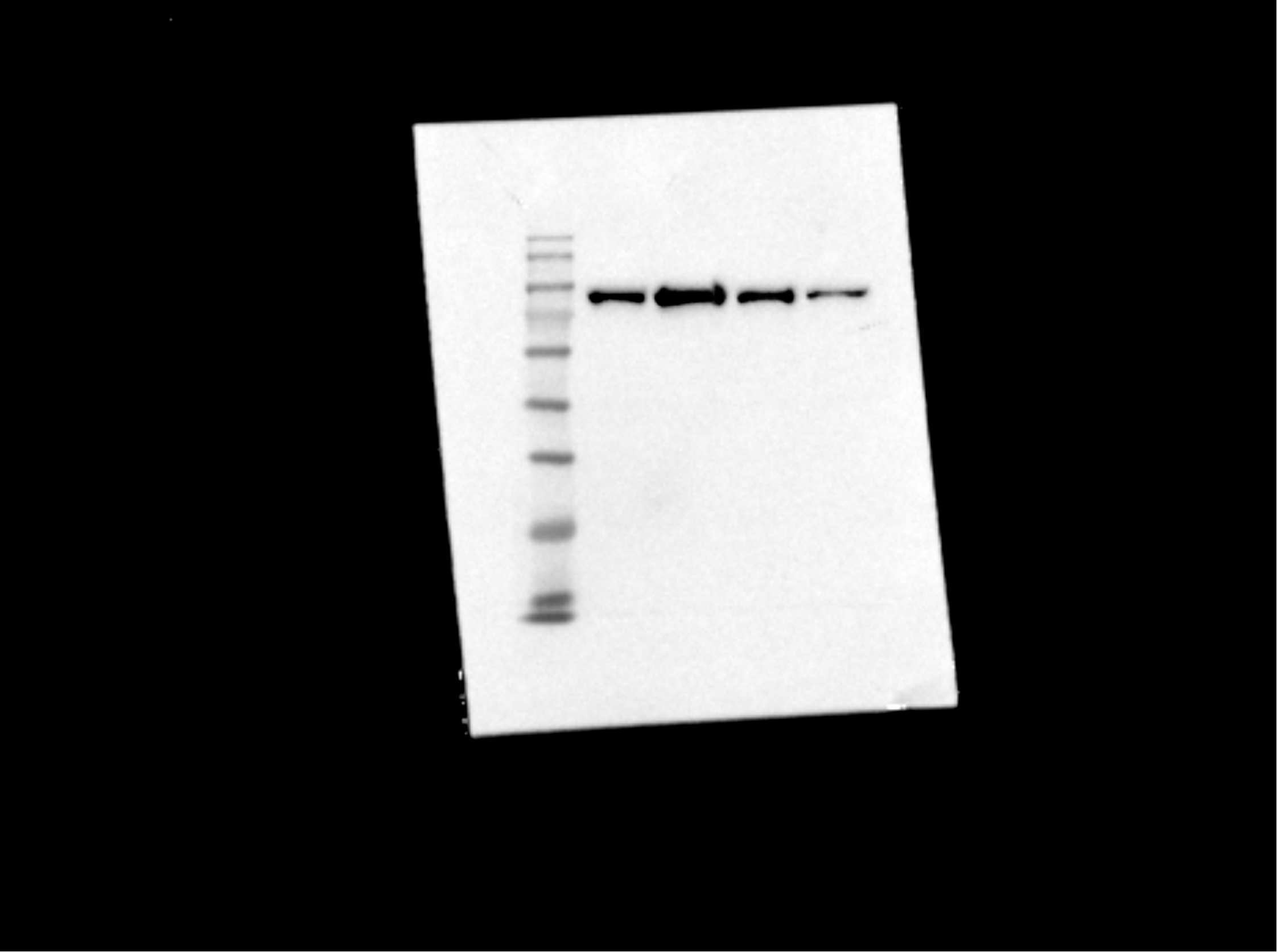


## Full and uncropped western blots of figure 4G-2.jpg


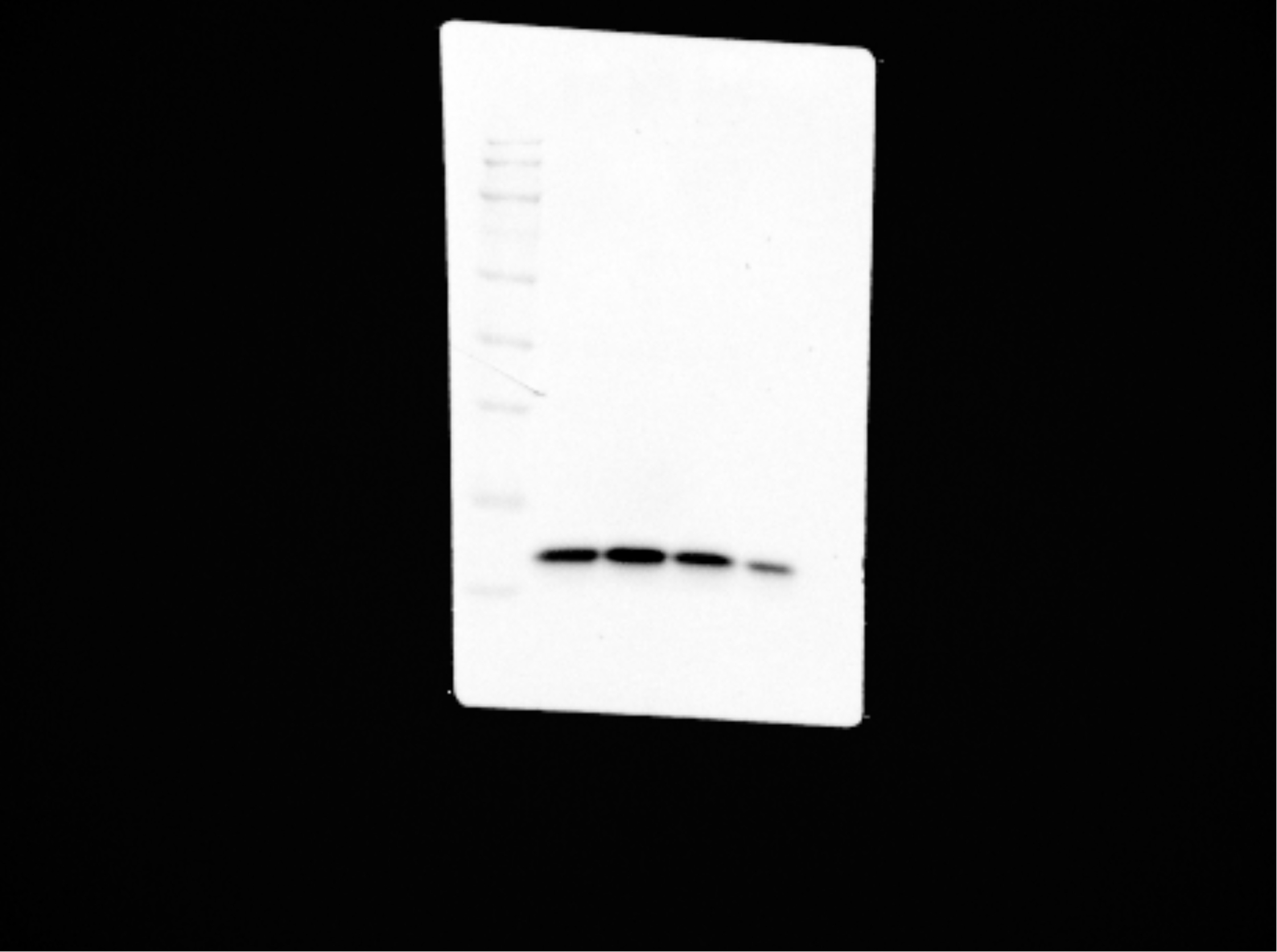


## Full and uncropped western blots of figure 4G-3.jpg


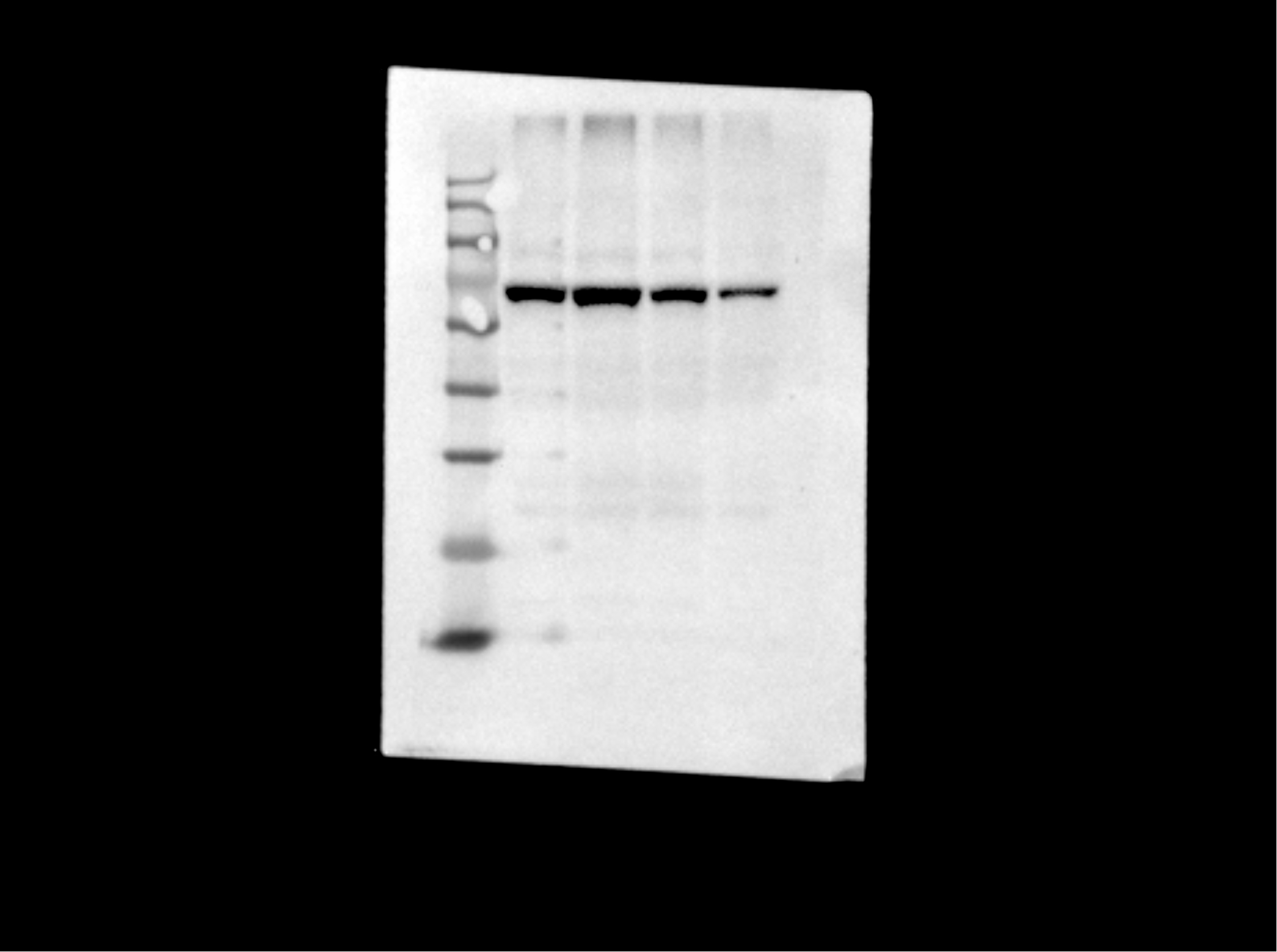


## Full and uncropped western blots of figure 4G-4.jpg


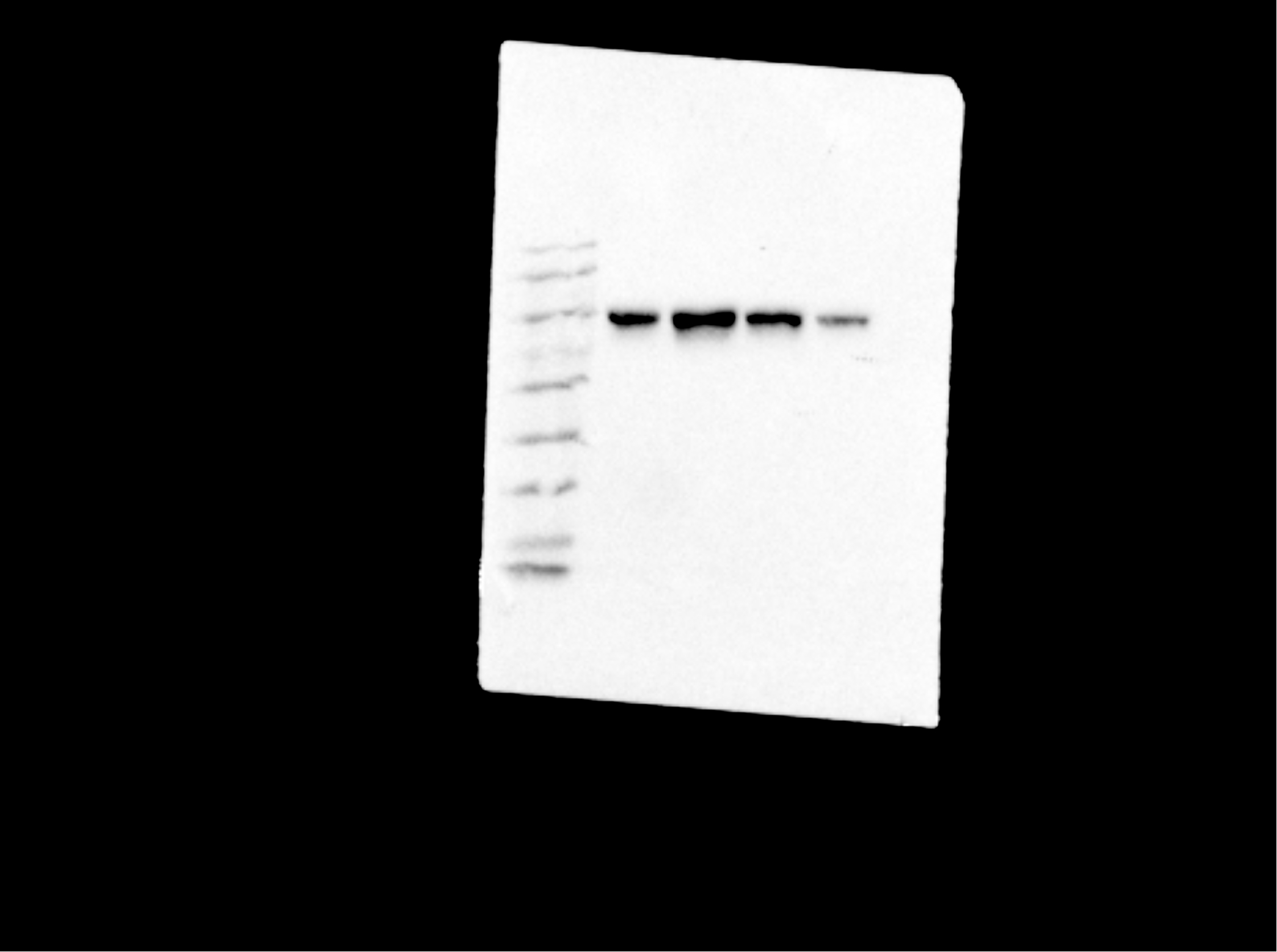


## Full and uncropped western blots of figure 4G-5.jpg


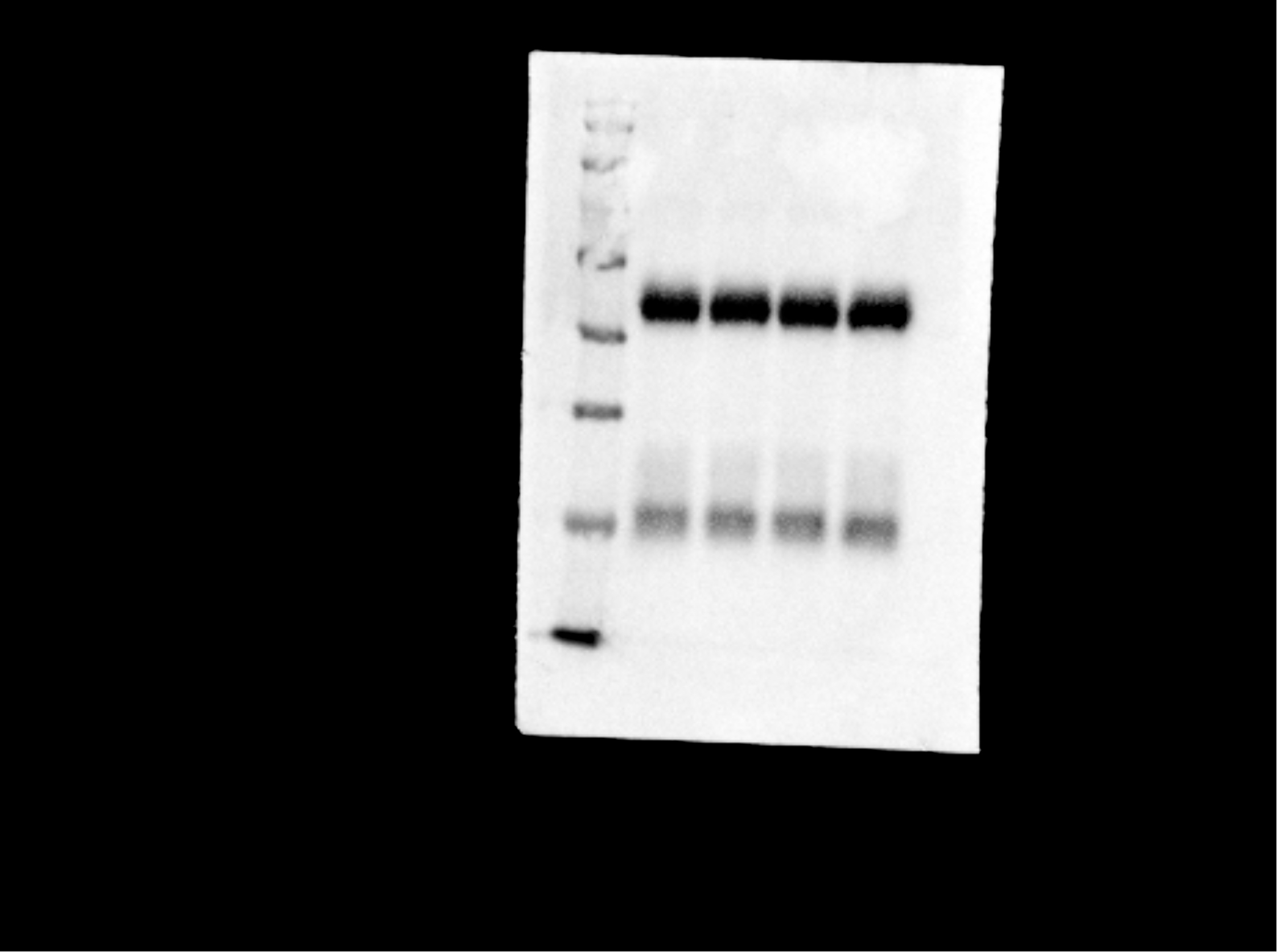


## Full and uncropped western blots of figure 5B-1.jpg


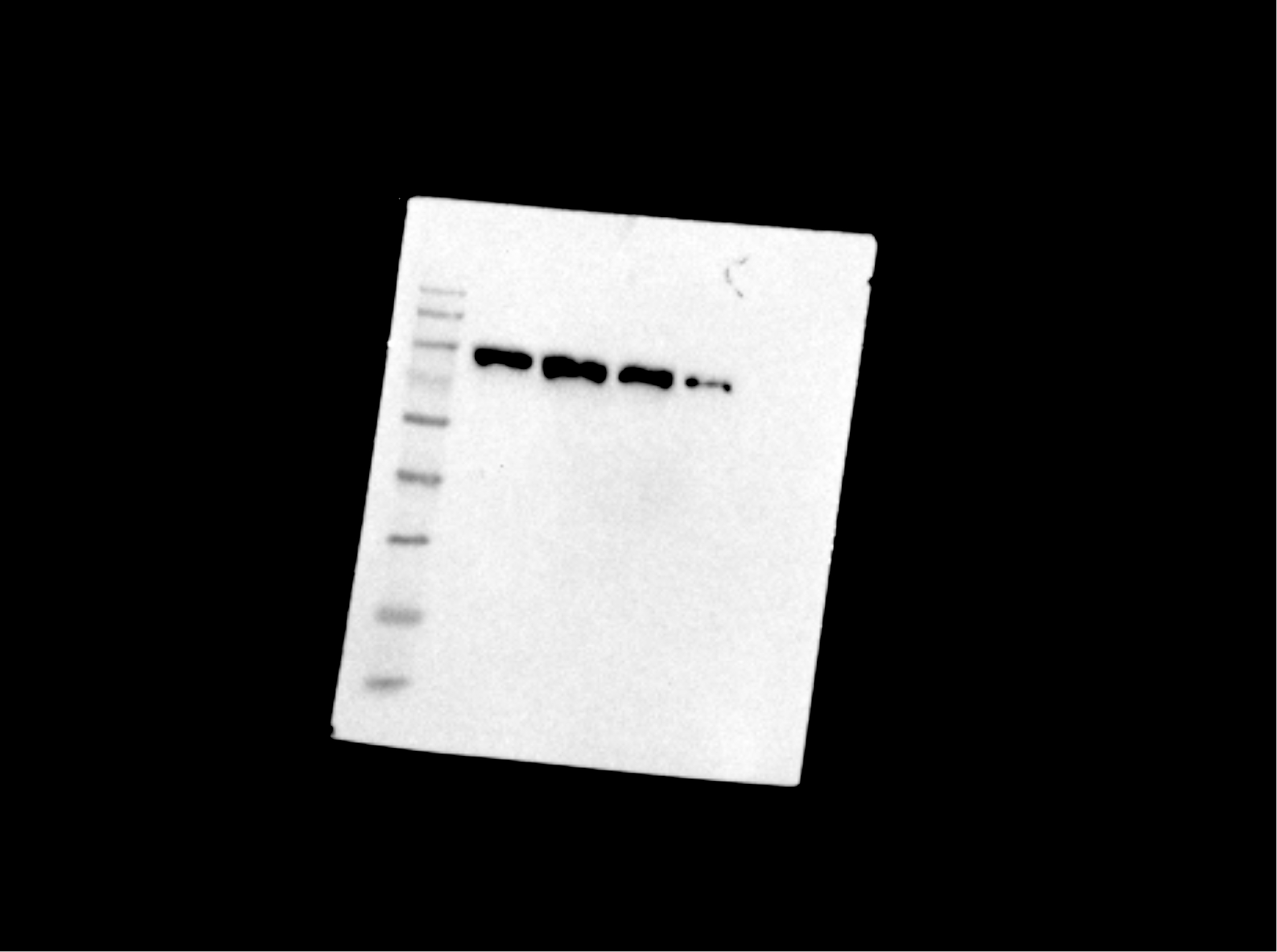


## Full and uncropped western blots of figure 5B-2.jpg


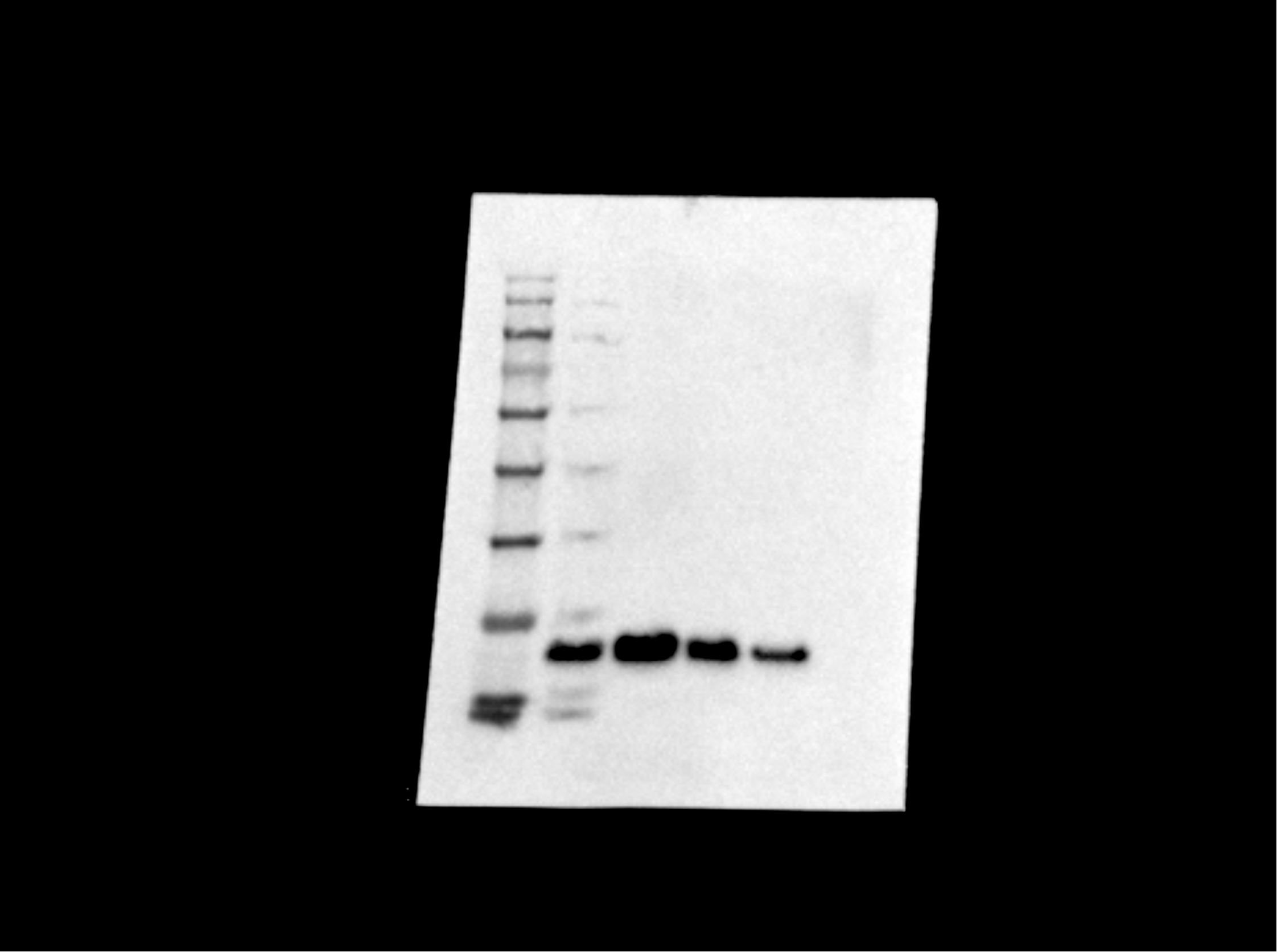


## Full and uncropped western blots of figure 5B-3.jpg


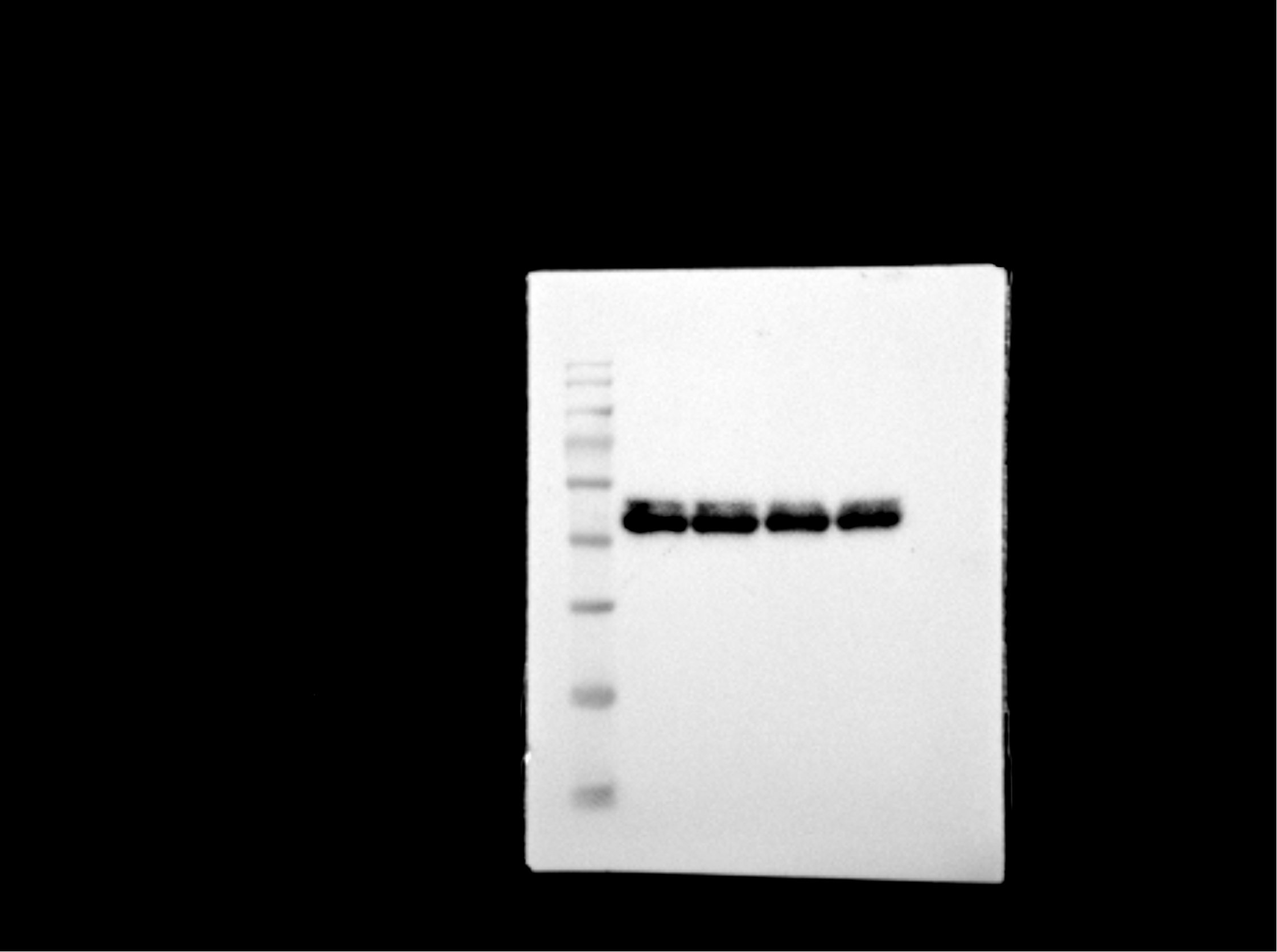


## Full and uncropped western blots of figure 7B-1.jpg


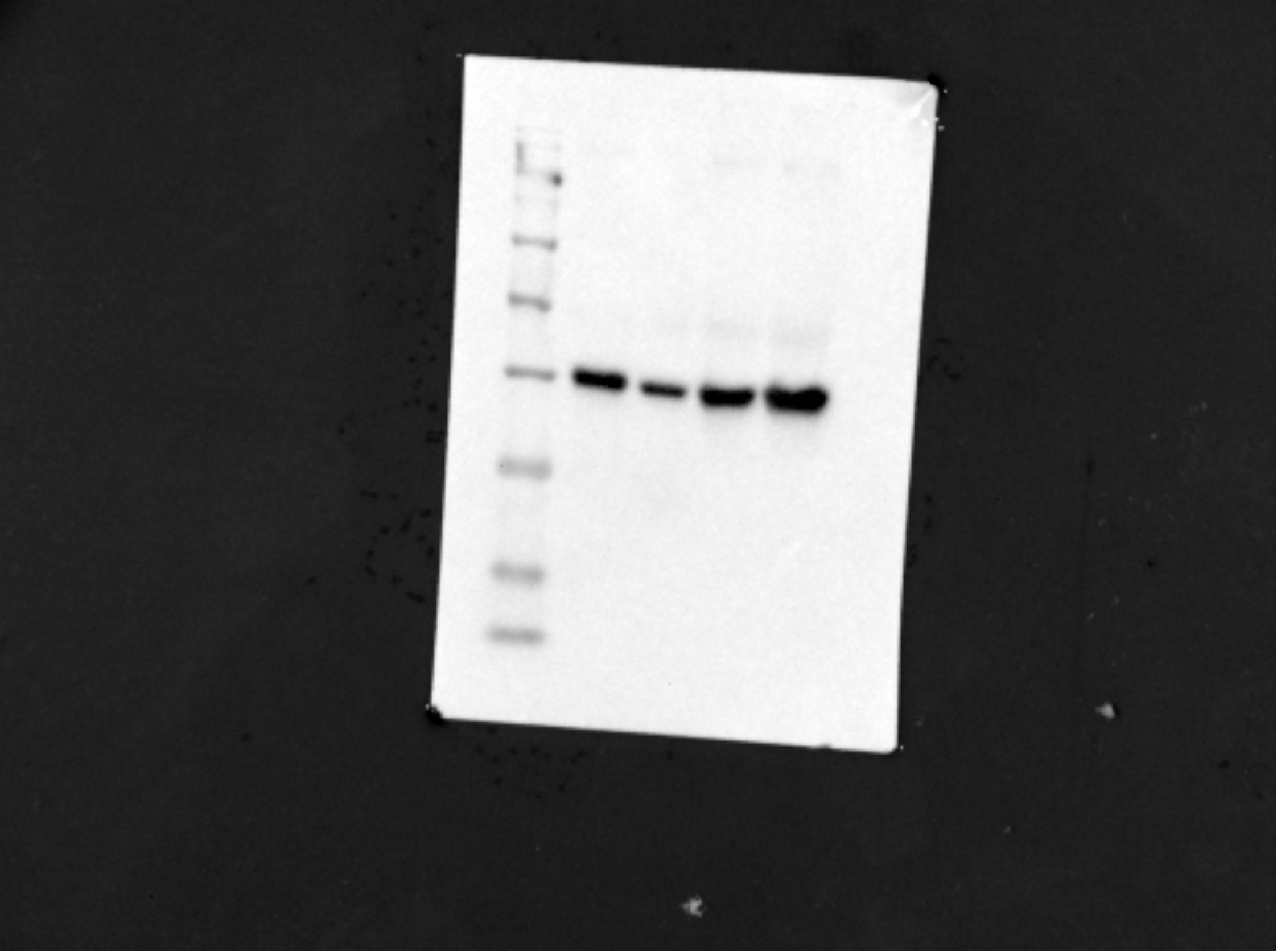


## Full and uncropped western blots of figure 7B-2.jpg


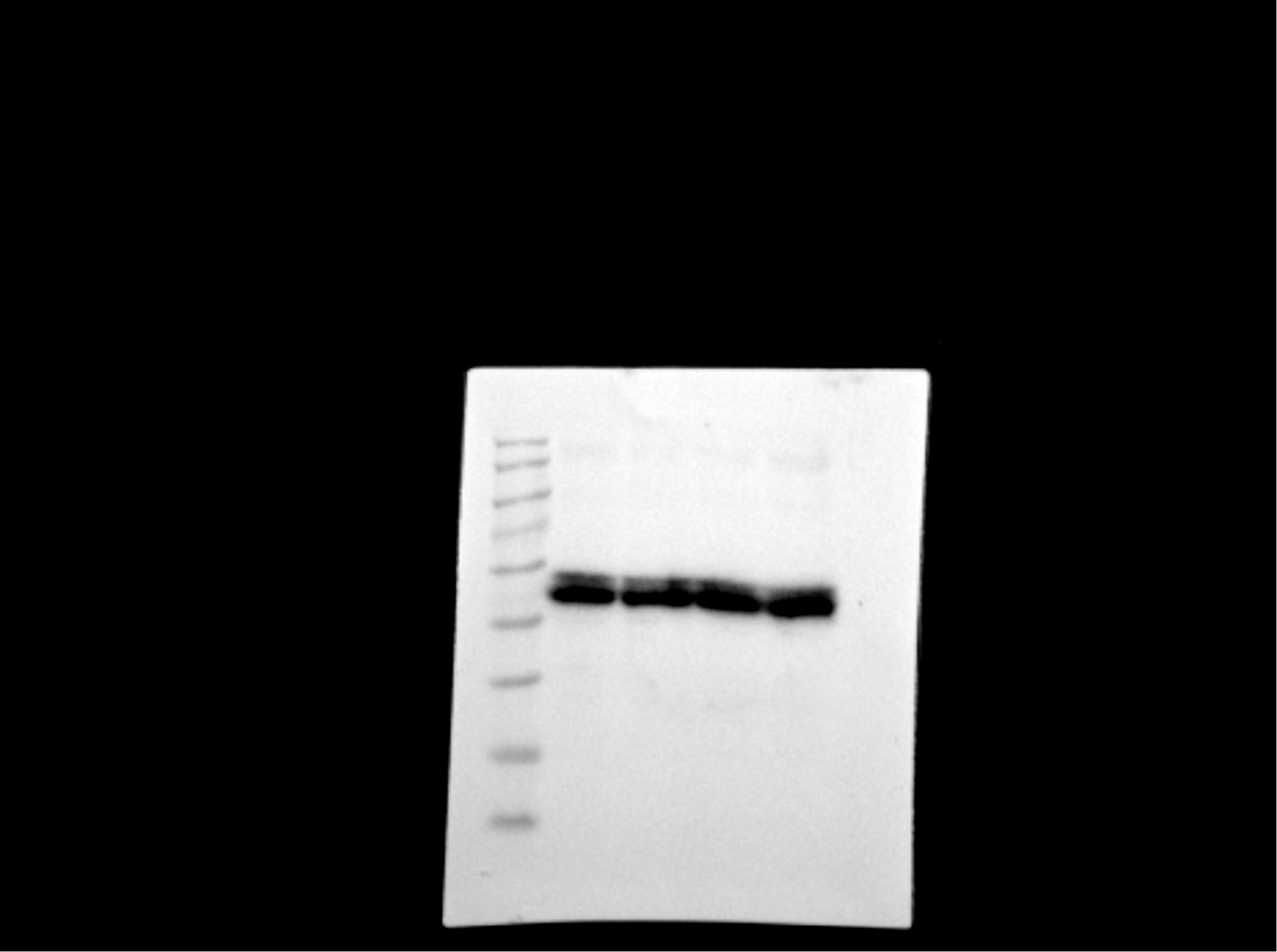


## Full and uncropped western blots of figure 7D-1.jpg


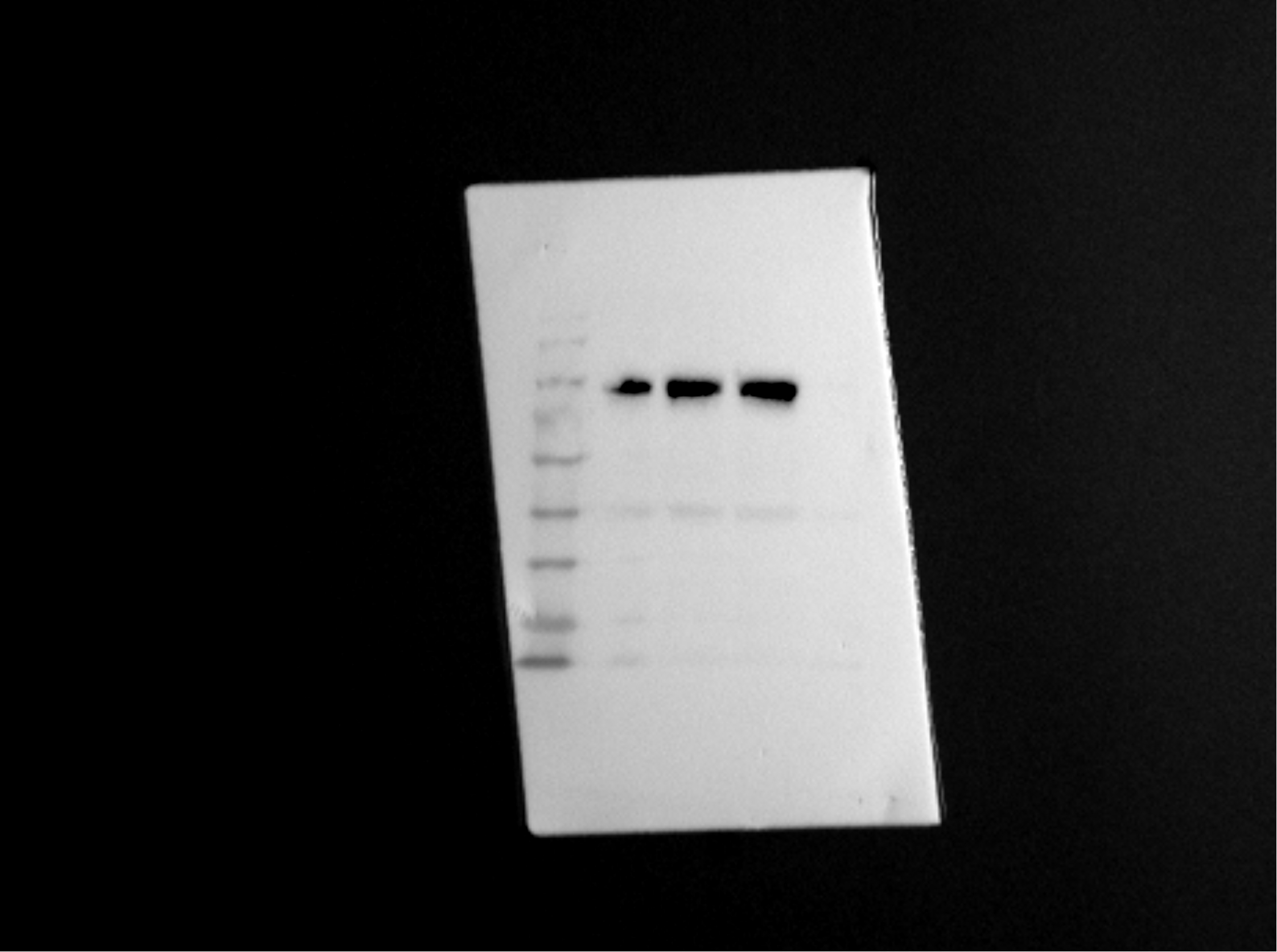


## Full and uncropped western blots of figure 7D-2.jpg


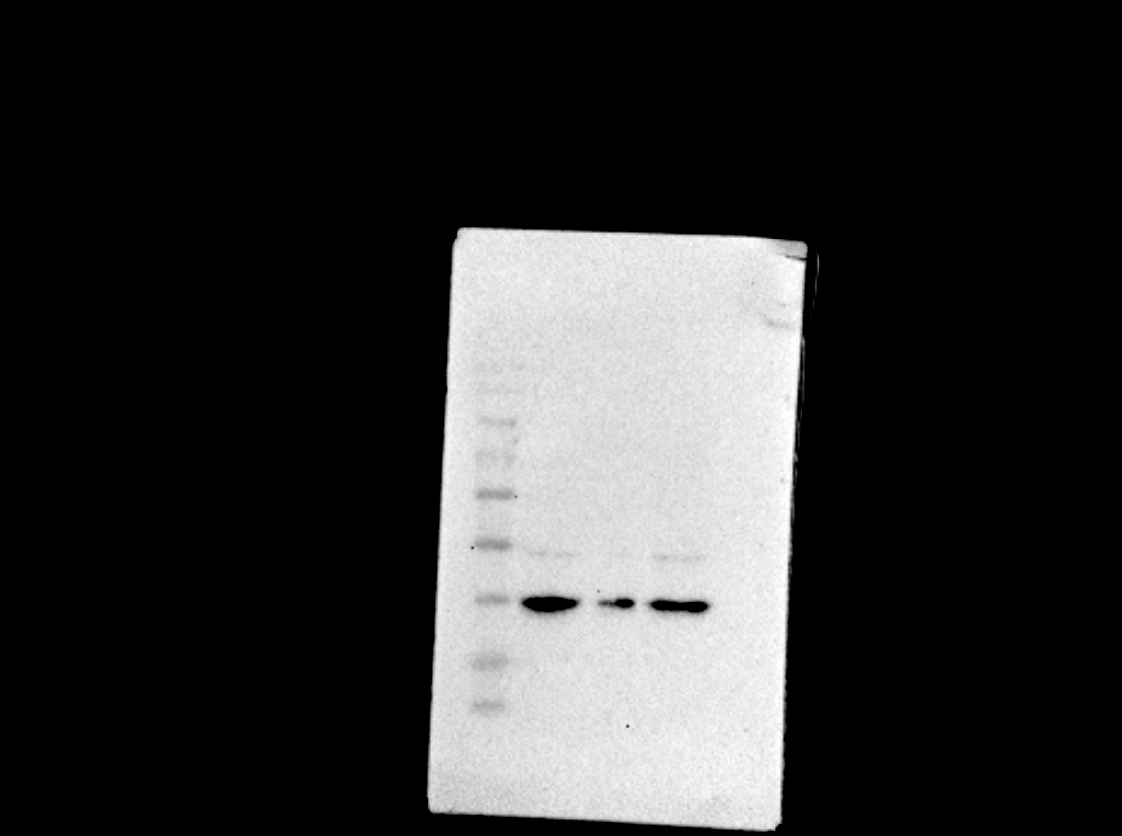


## Full and uncropped western blots of figure 7D-3.jpg


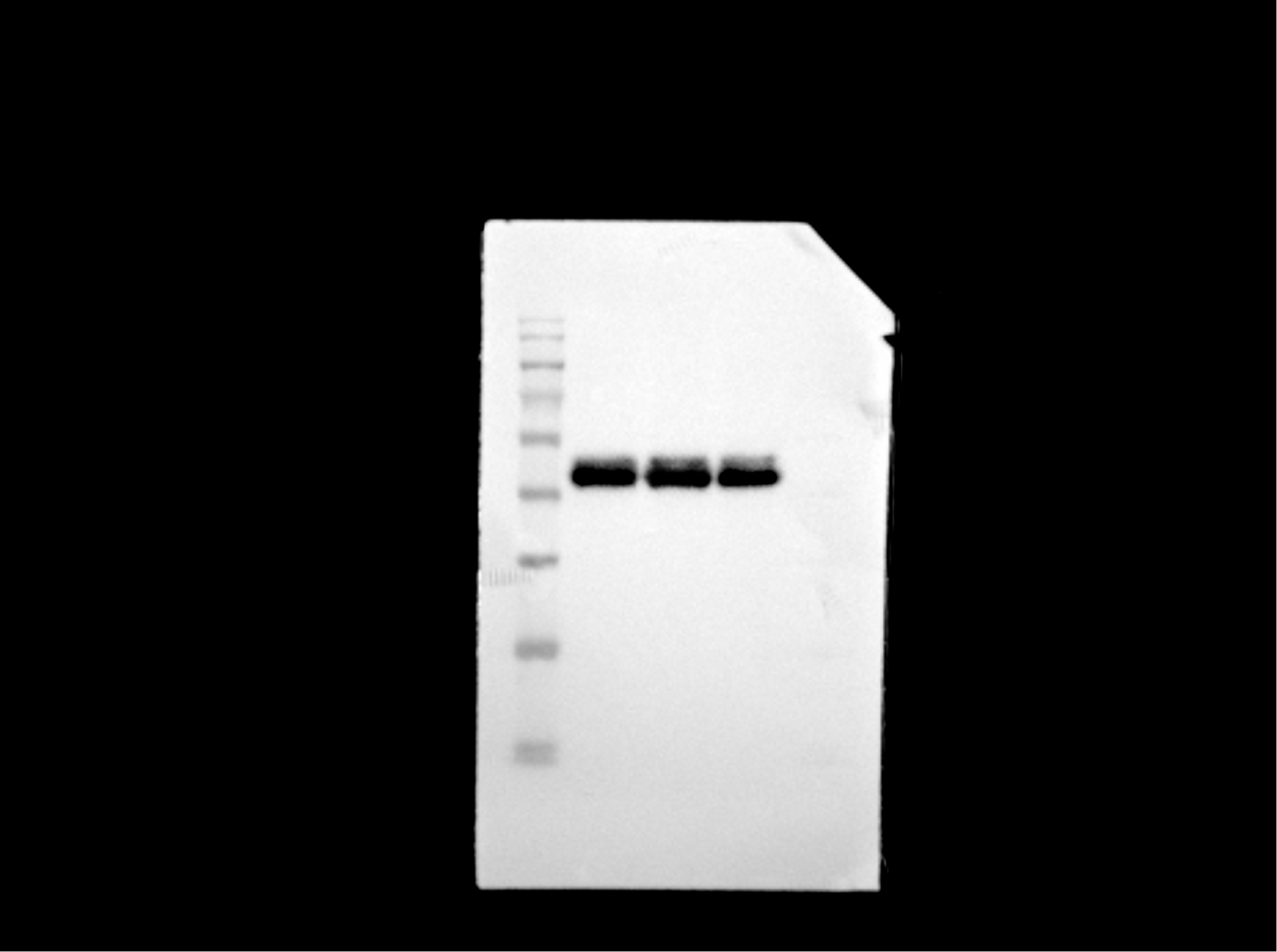


## Full and uncropped western blots of figure 7E-1.jpg


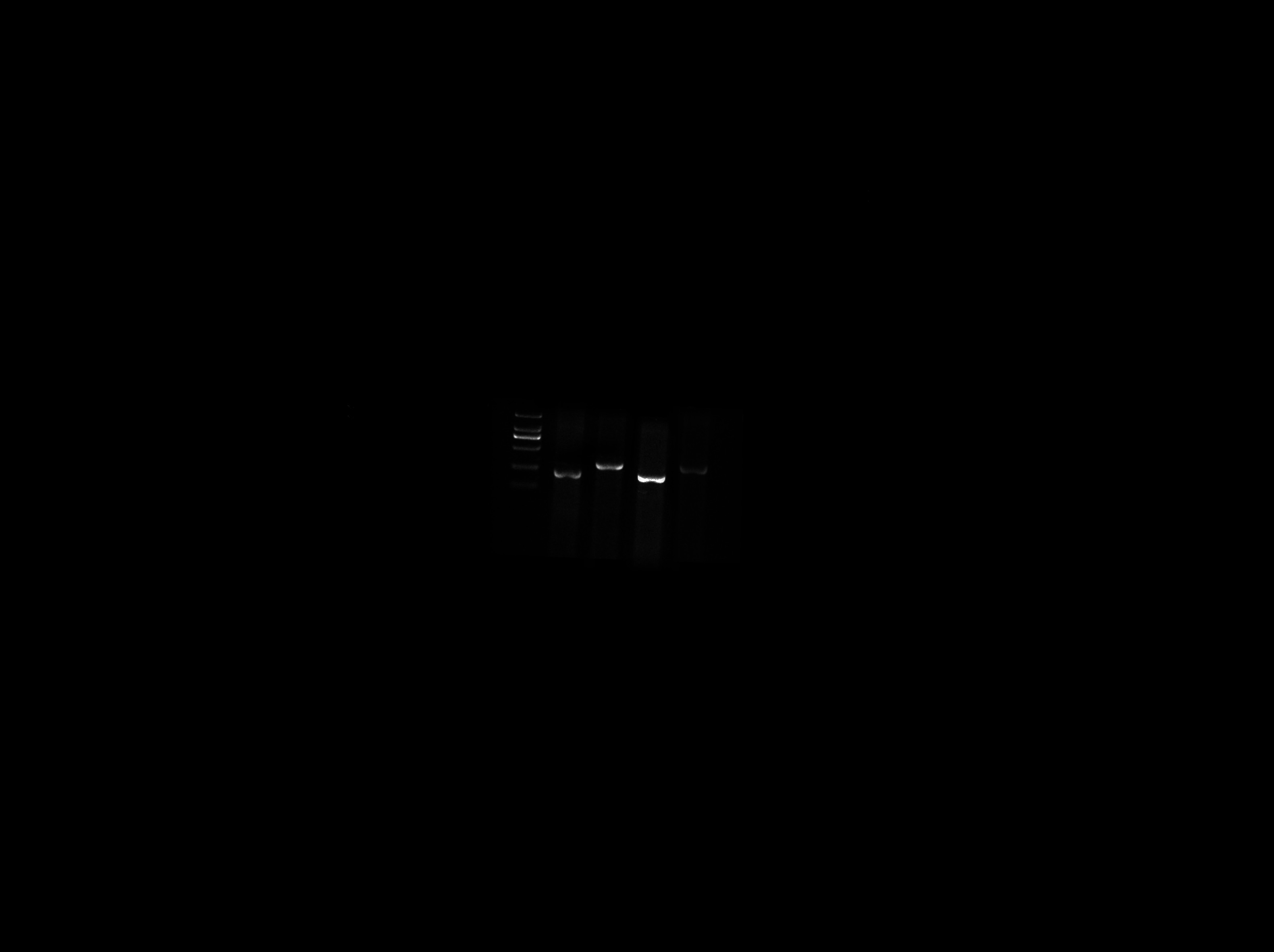


## Full and uncropped western blots of figure 7E-2.jpg


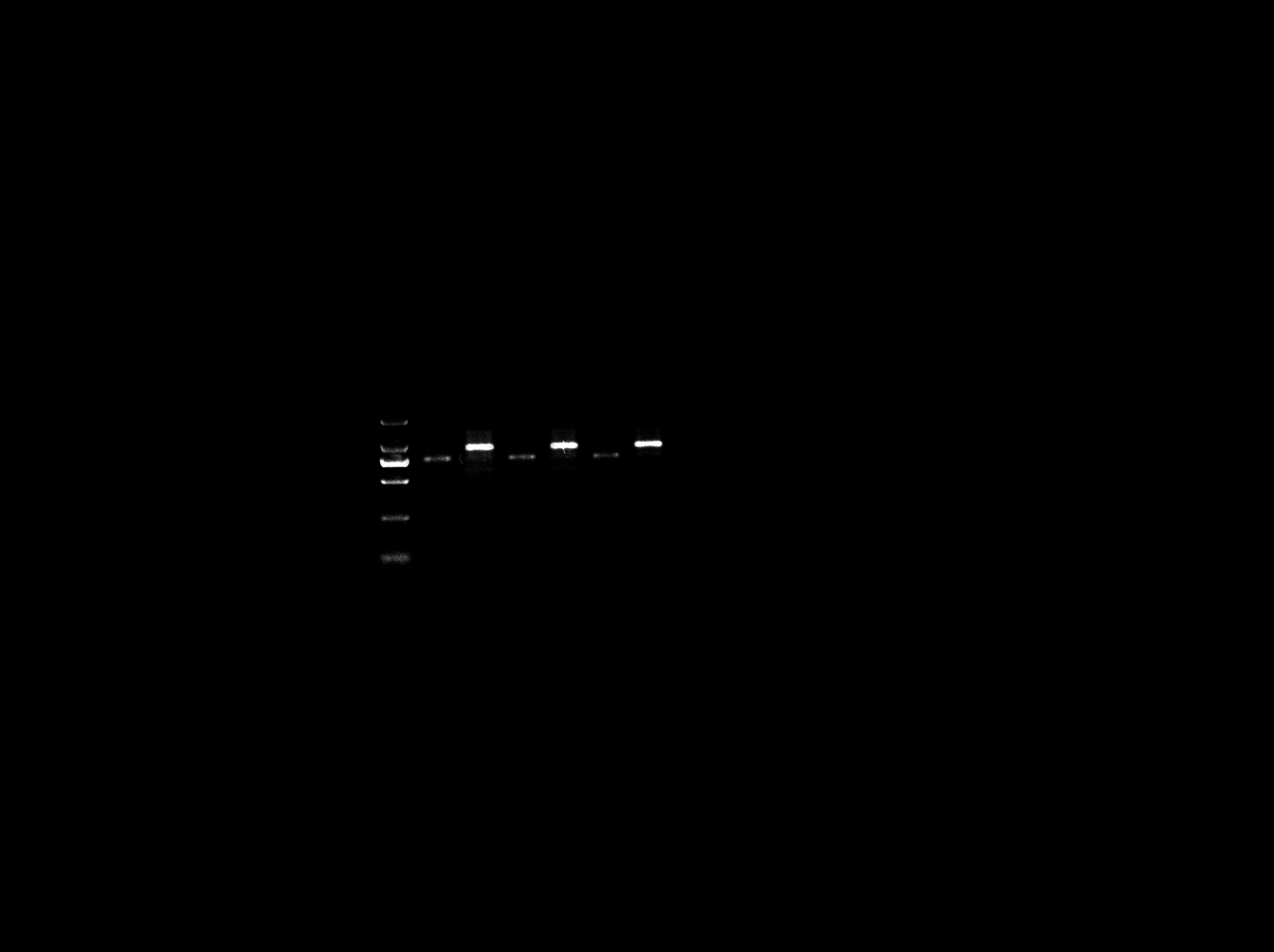


## Full and uncropped western blots of figure 8A.jpg


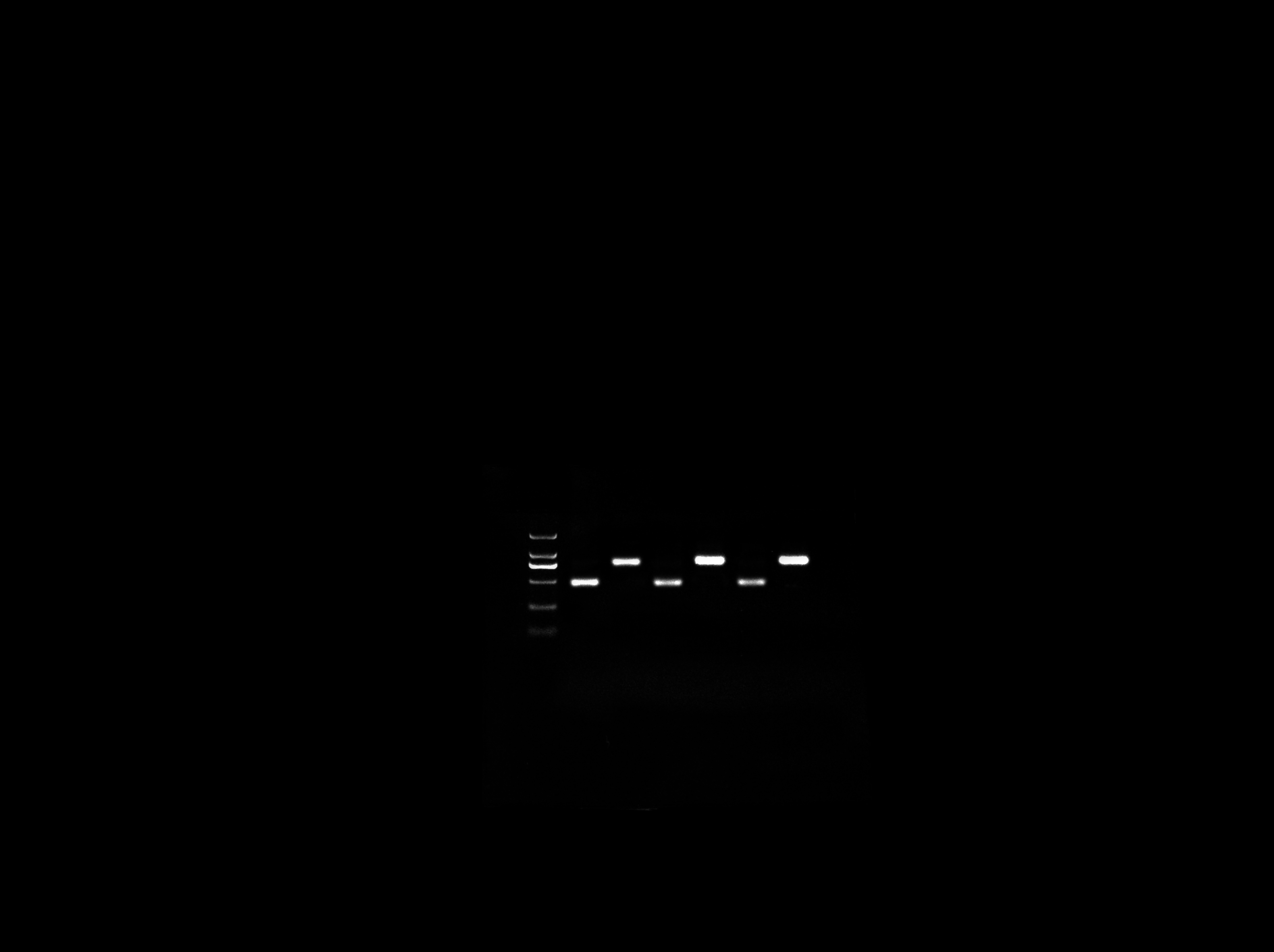


## Full and uncropped western blots of figure 8C-1.jpg


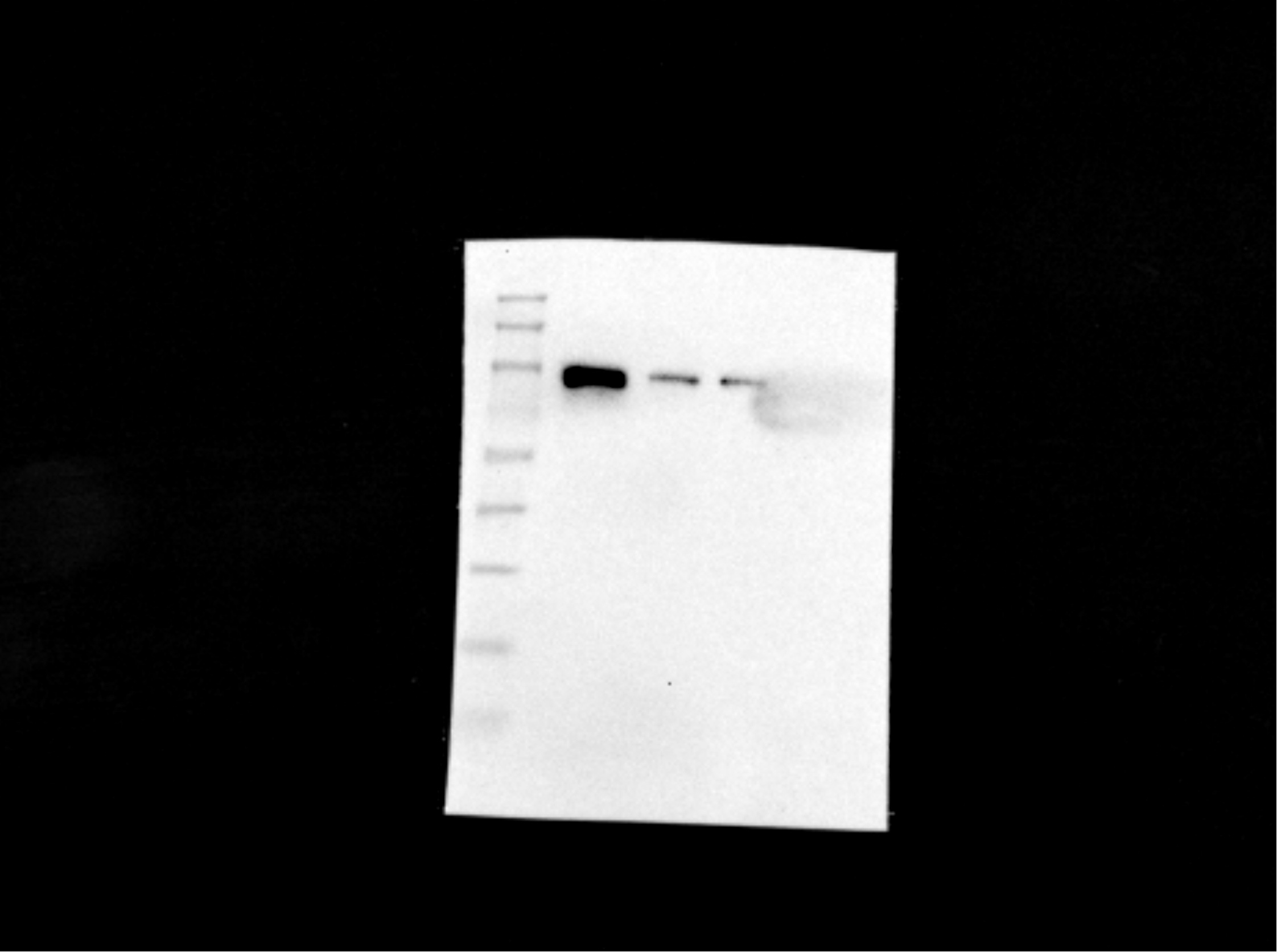


## Full and uncropped western blots of figure 8C-2.jpg


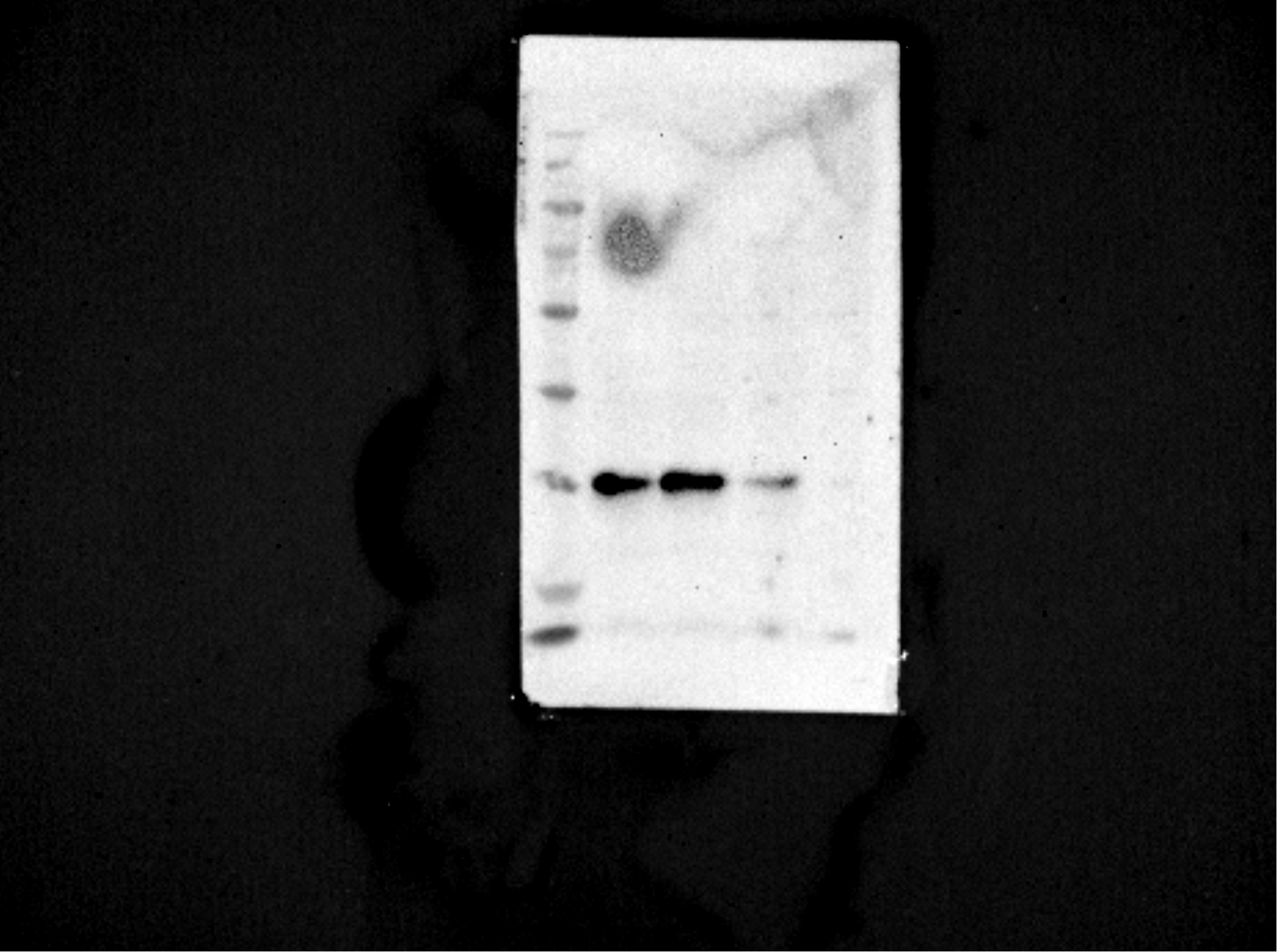


## Full and uncropped western blots of figure 8C-3.jpg


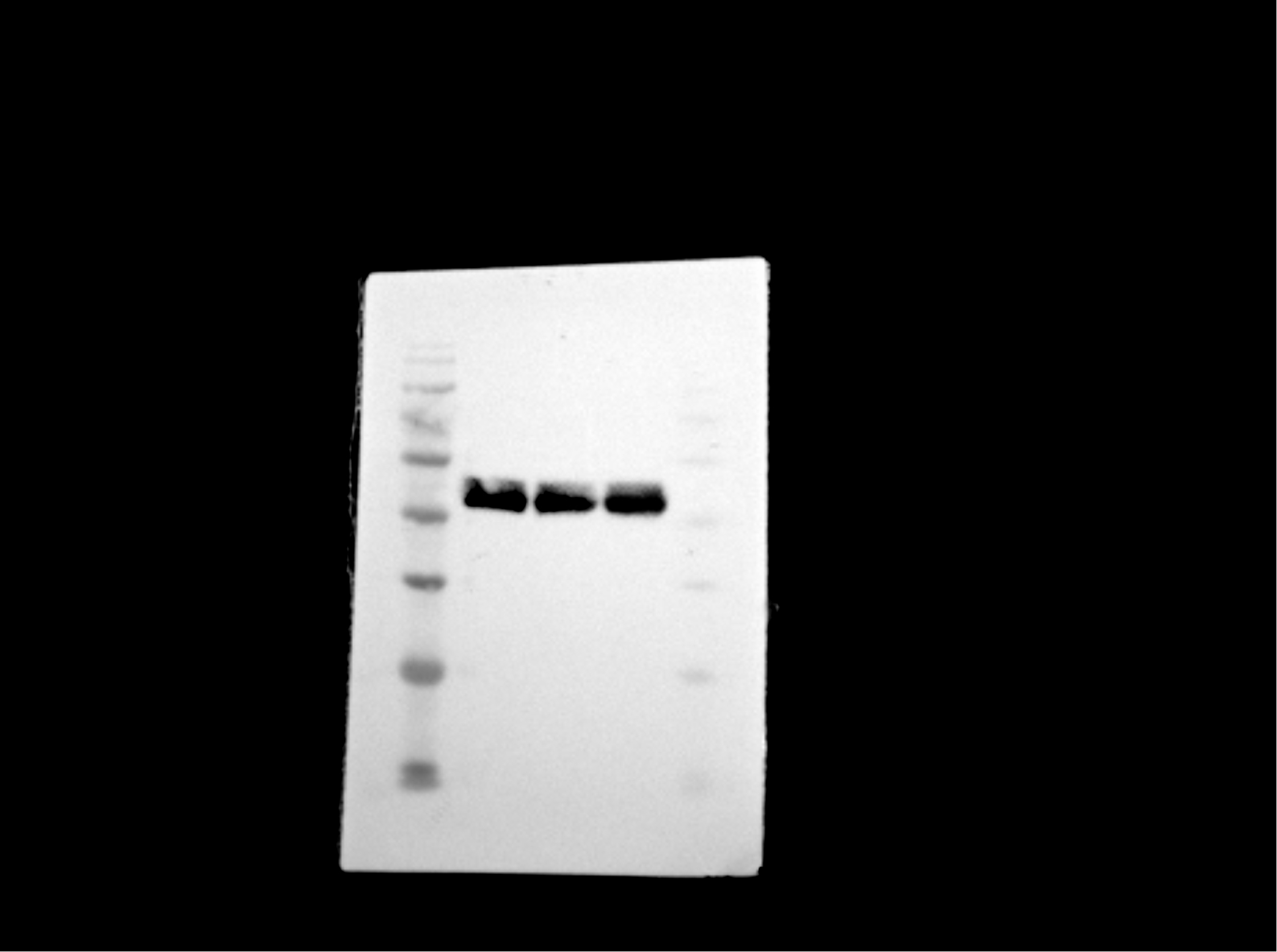


## Full and uncropped western blots of figure 8J-1.jpg


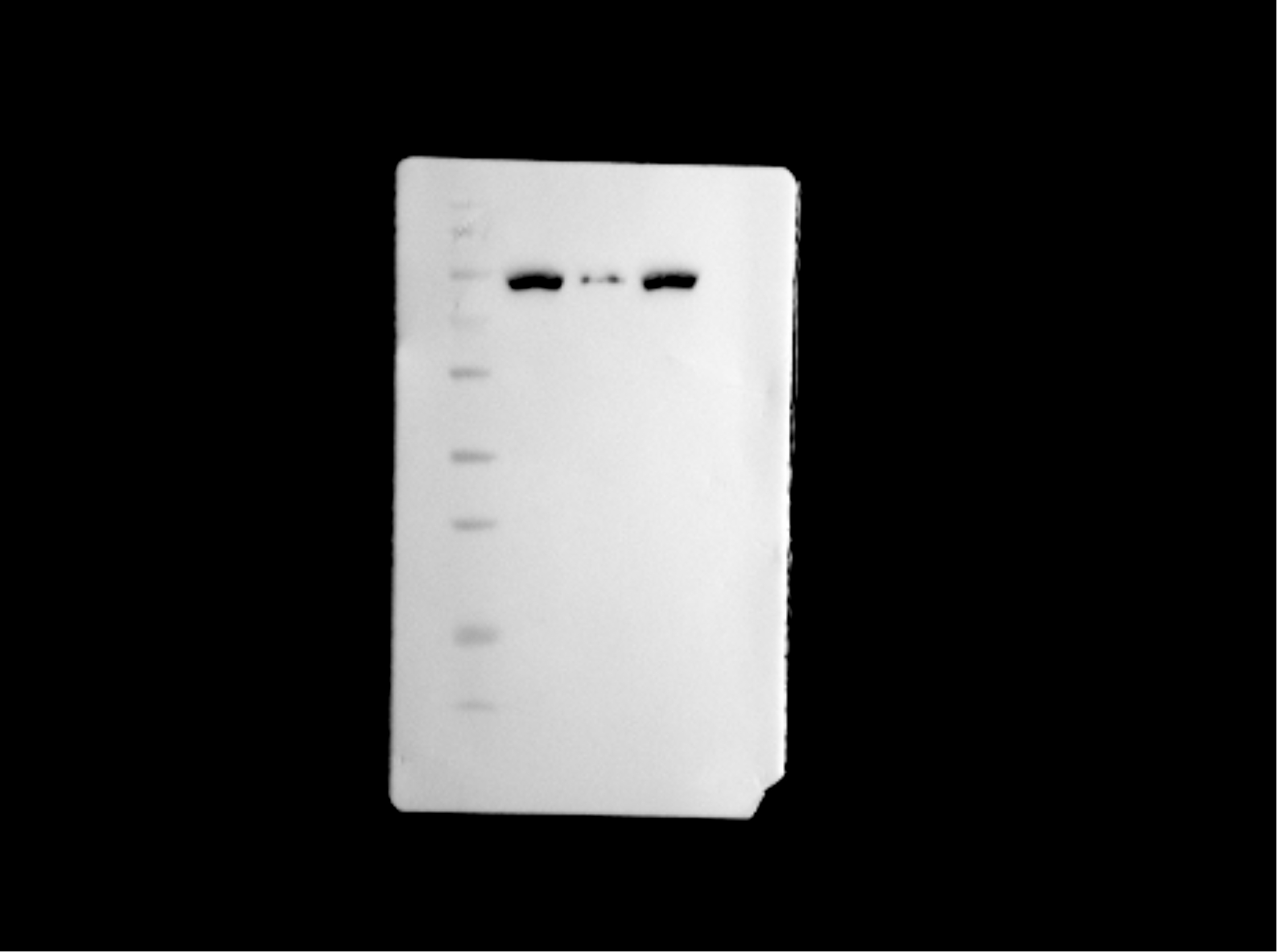


## Full and uncropped western blots of figure 8J-2.jpg


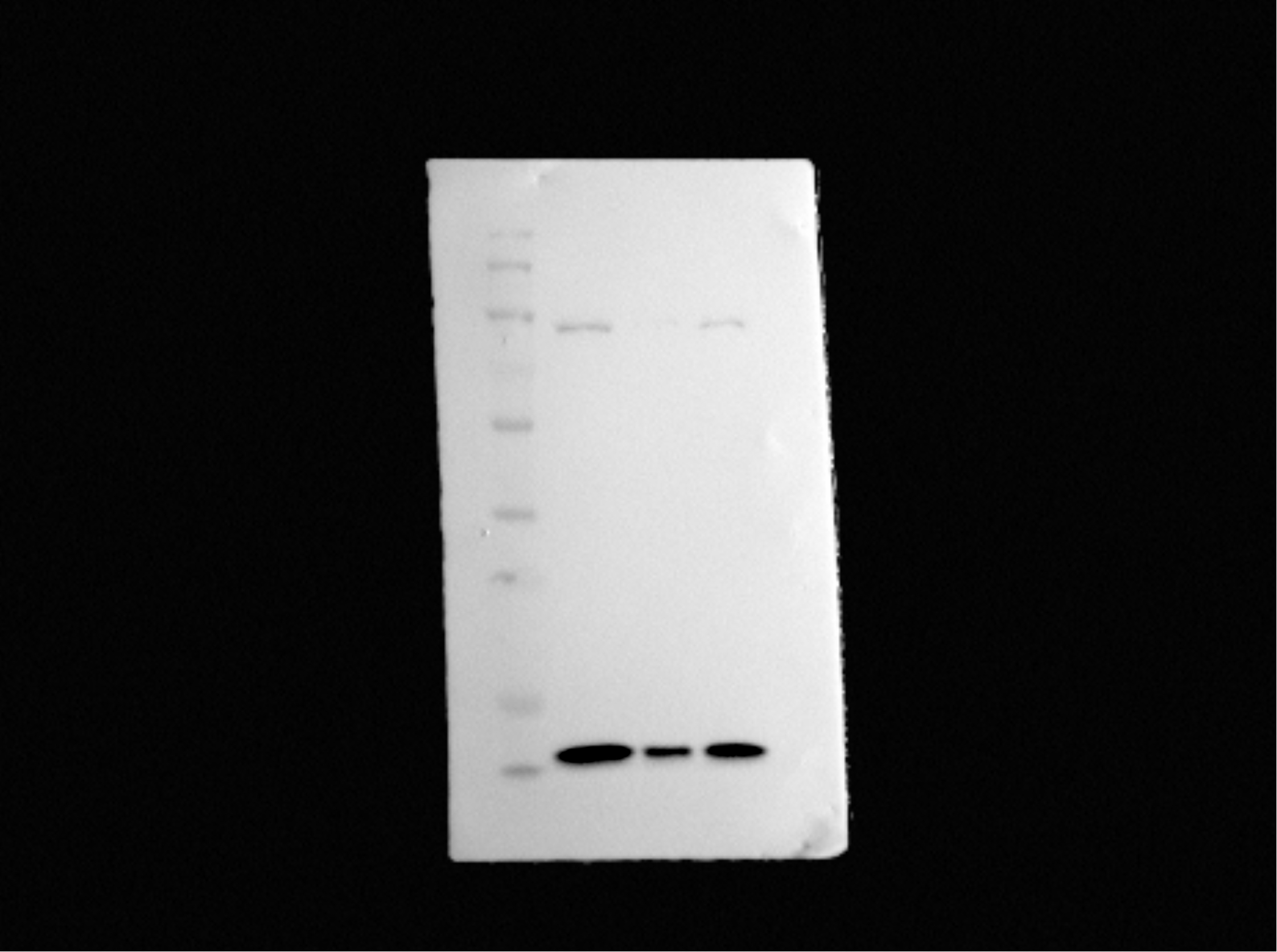


## Full and uncropped western blots of figure 8J-3.jpg


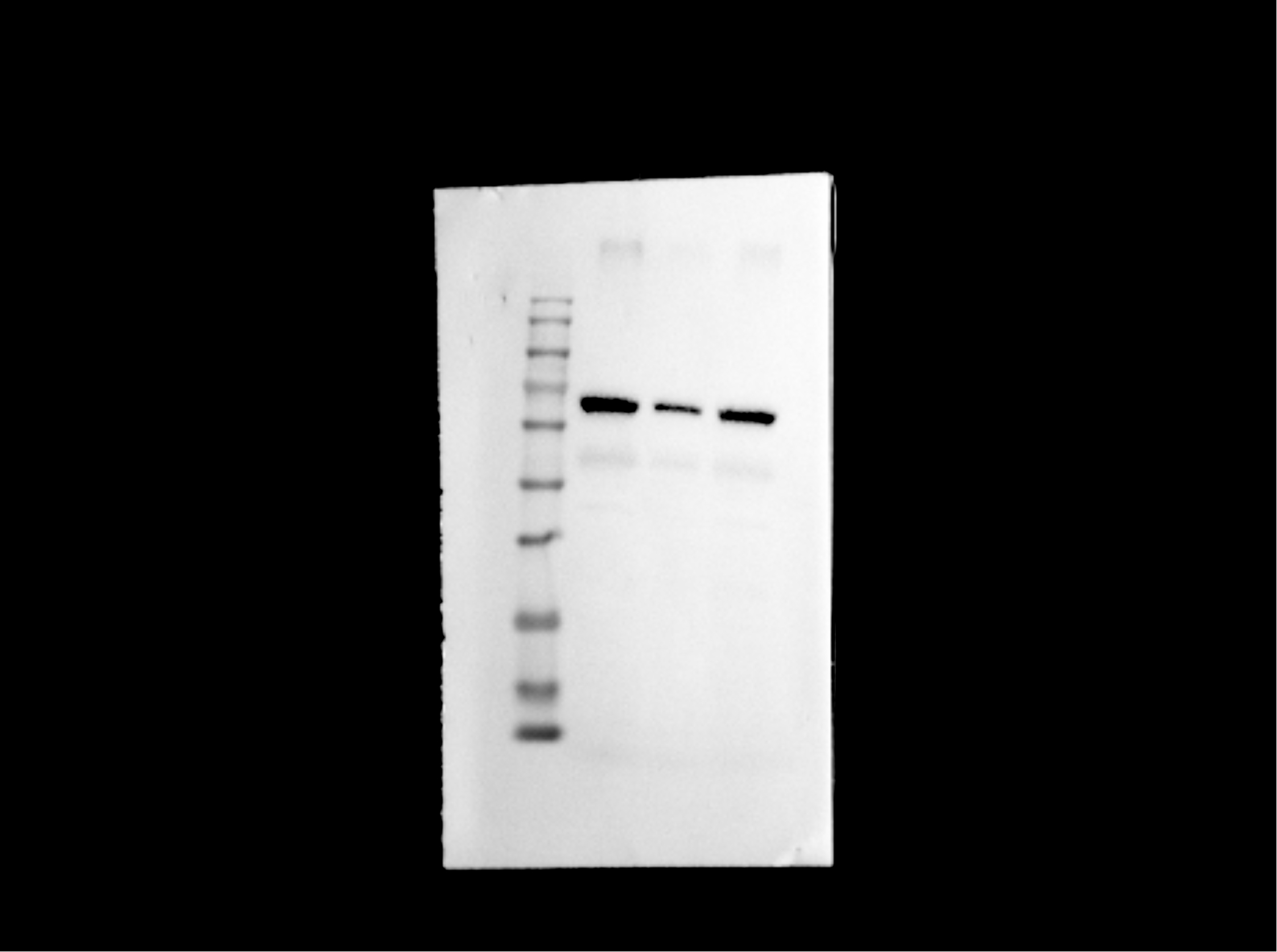


## Full and uncropped western blots of figure 8J-4.jpg


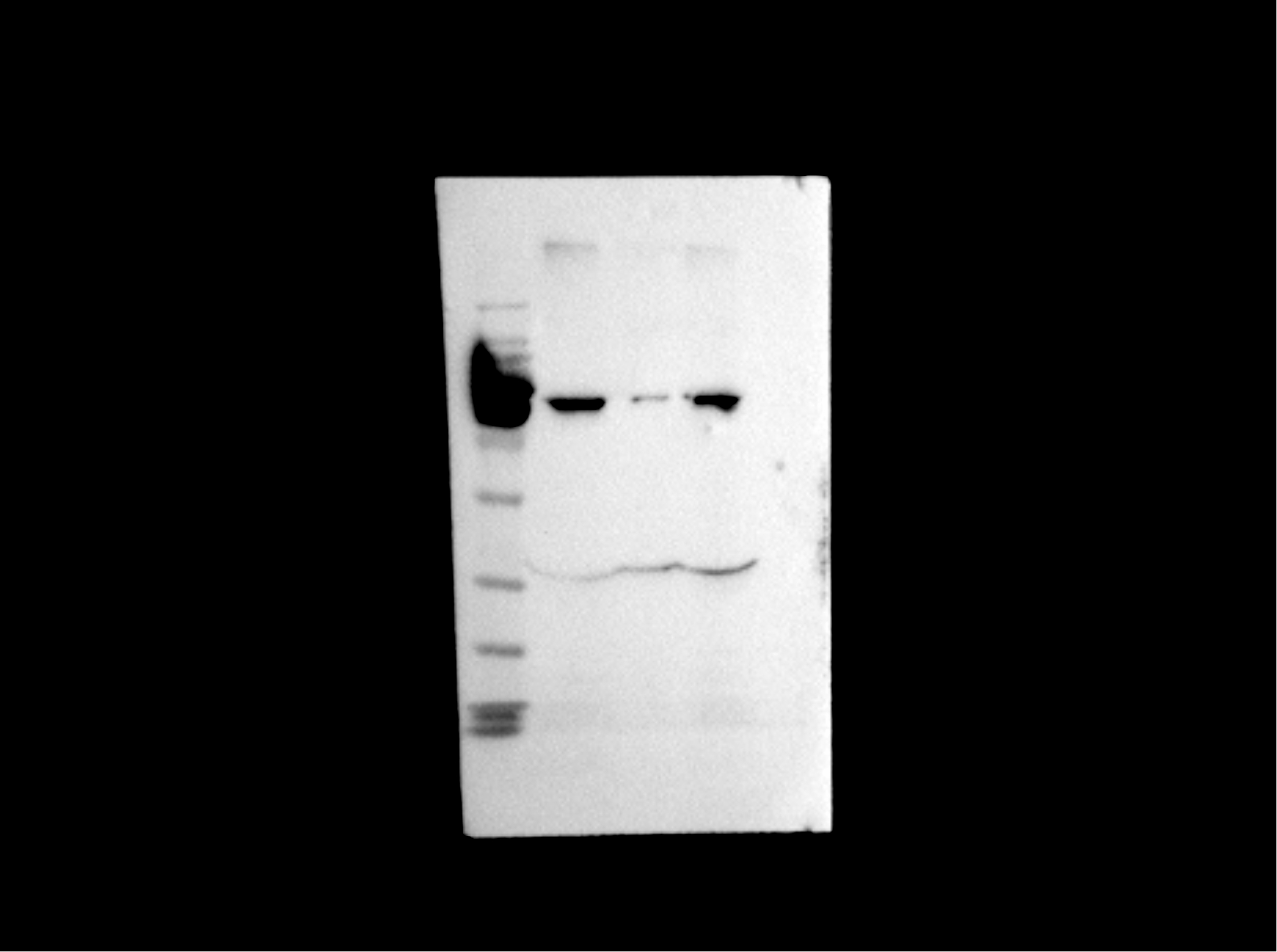


## Full and uncropped western blots of figure 8J-5.jpg


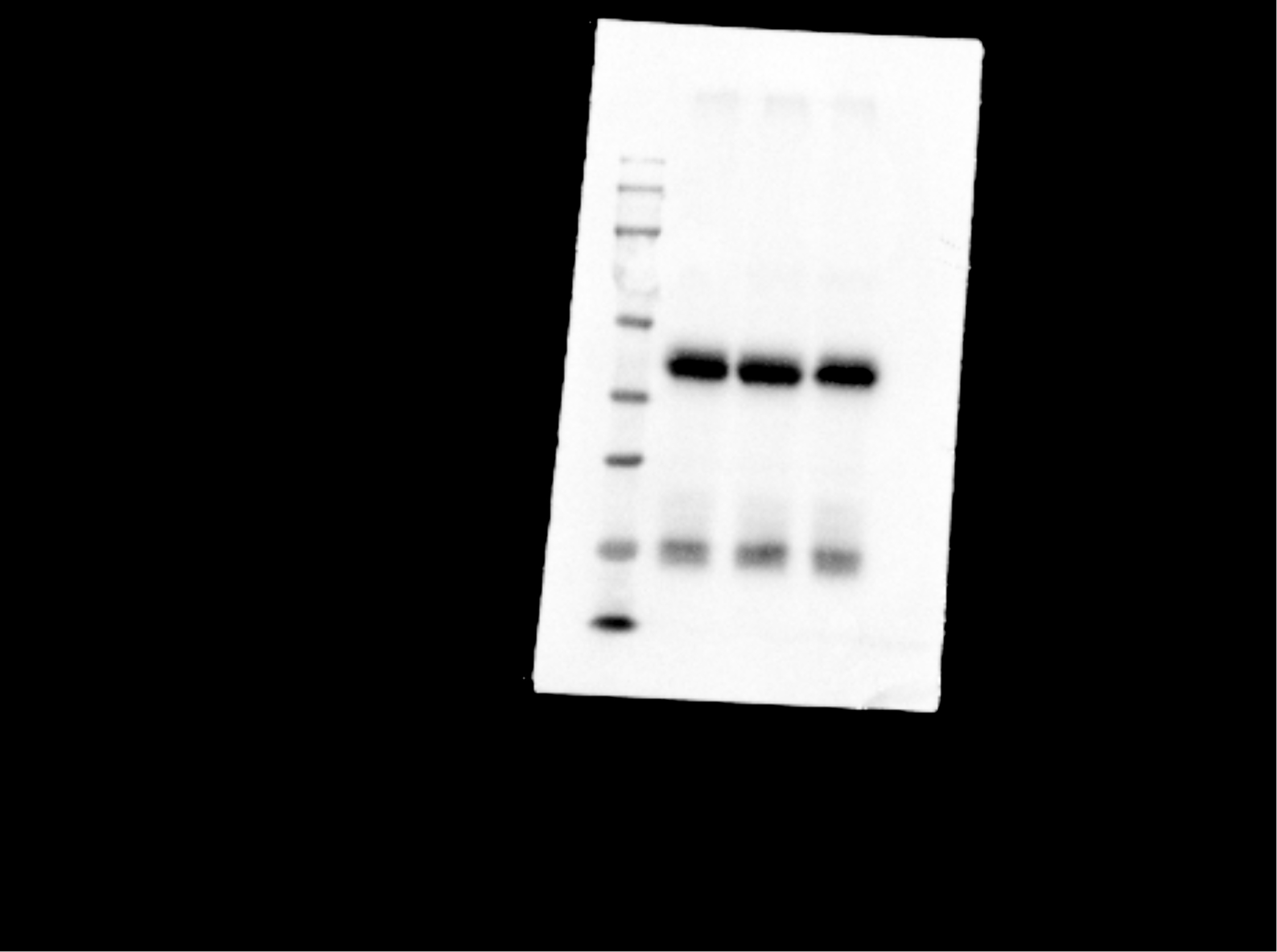


## Full and uncropped western blots of figure 9A.jpg


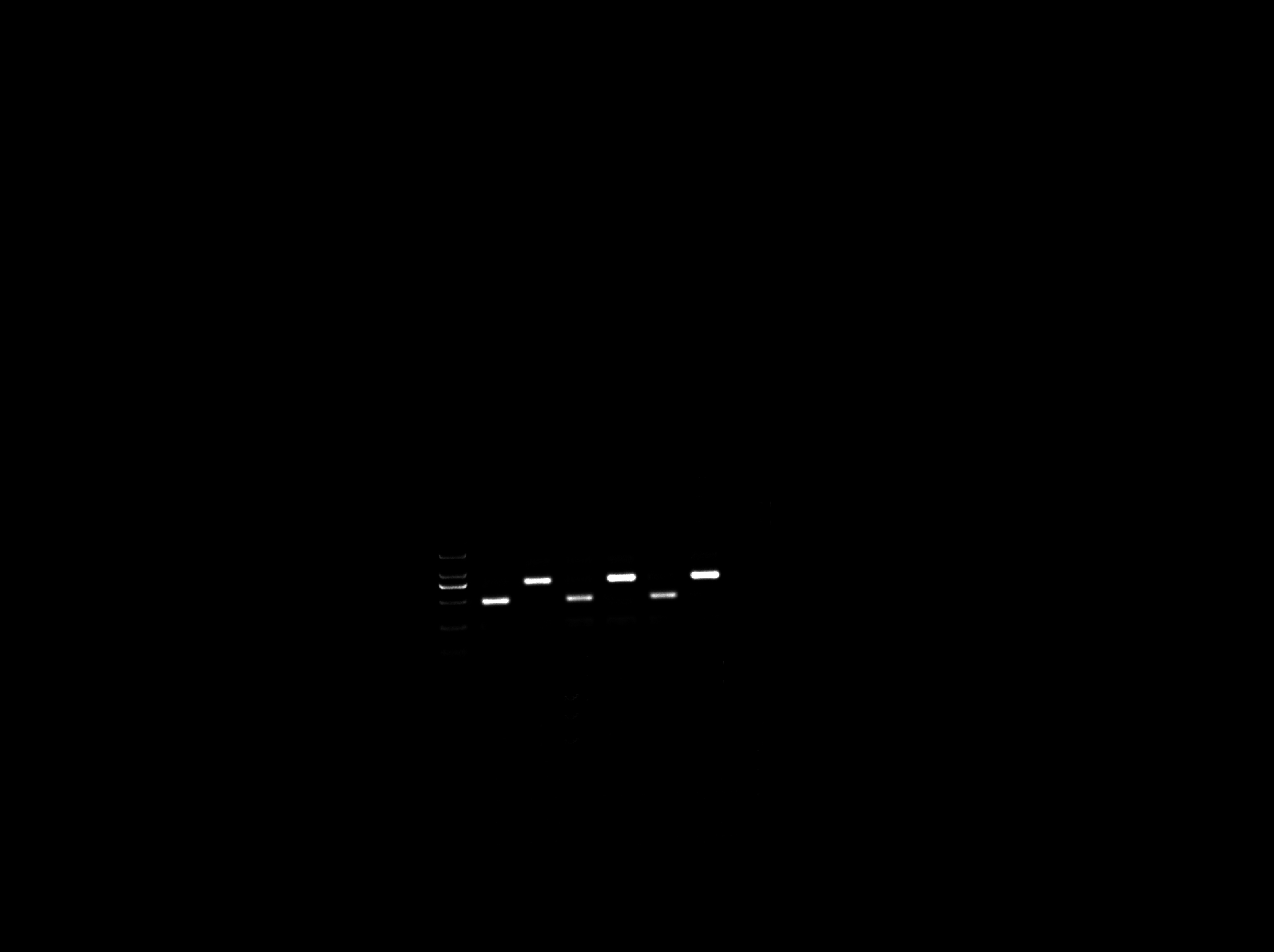


## Full and uncropped western blots of figure 9B-1.jpg


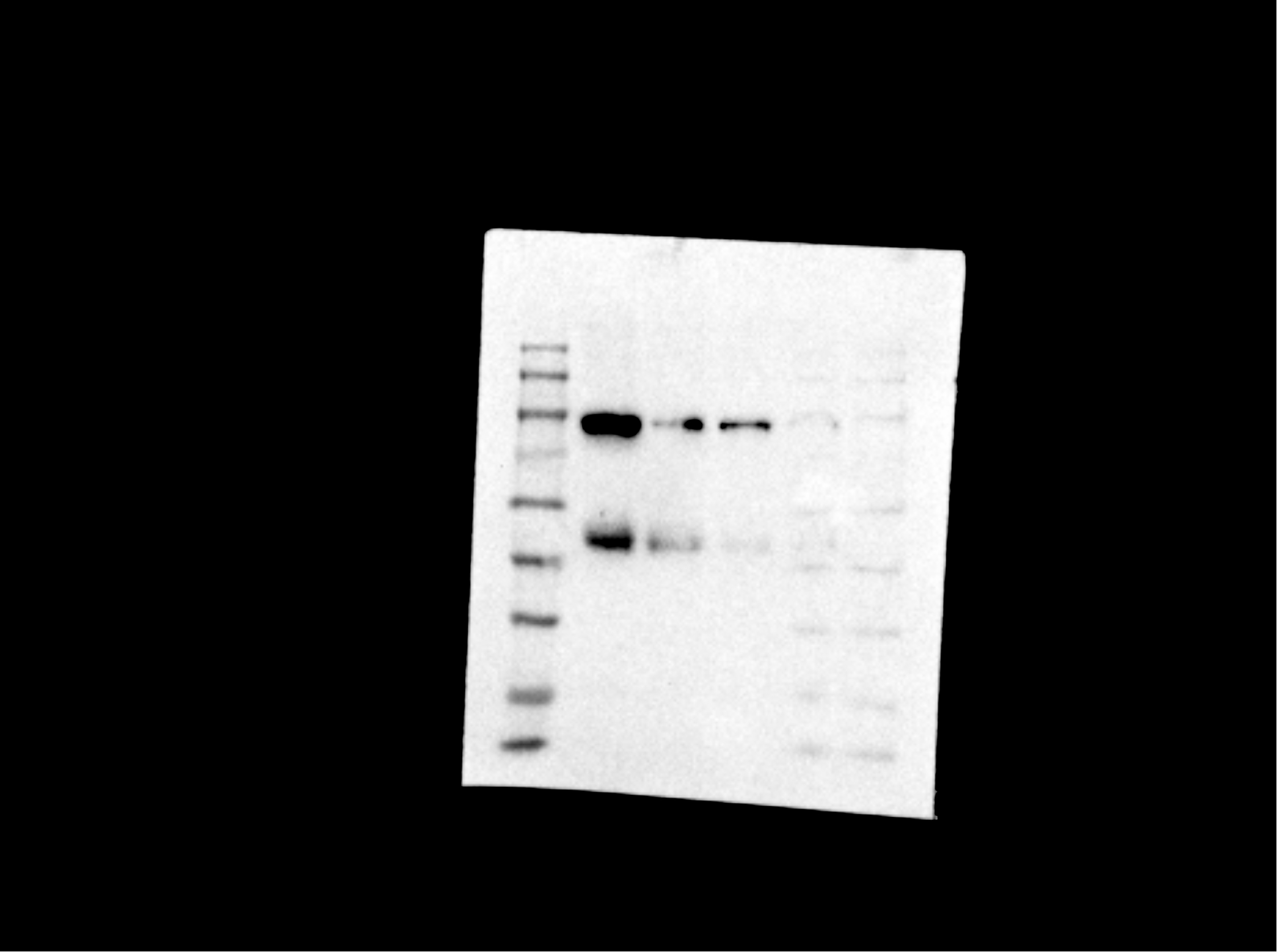


## Full and uncropped western blots of figure 9B-2.jpg


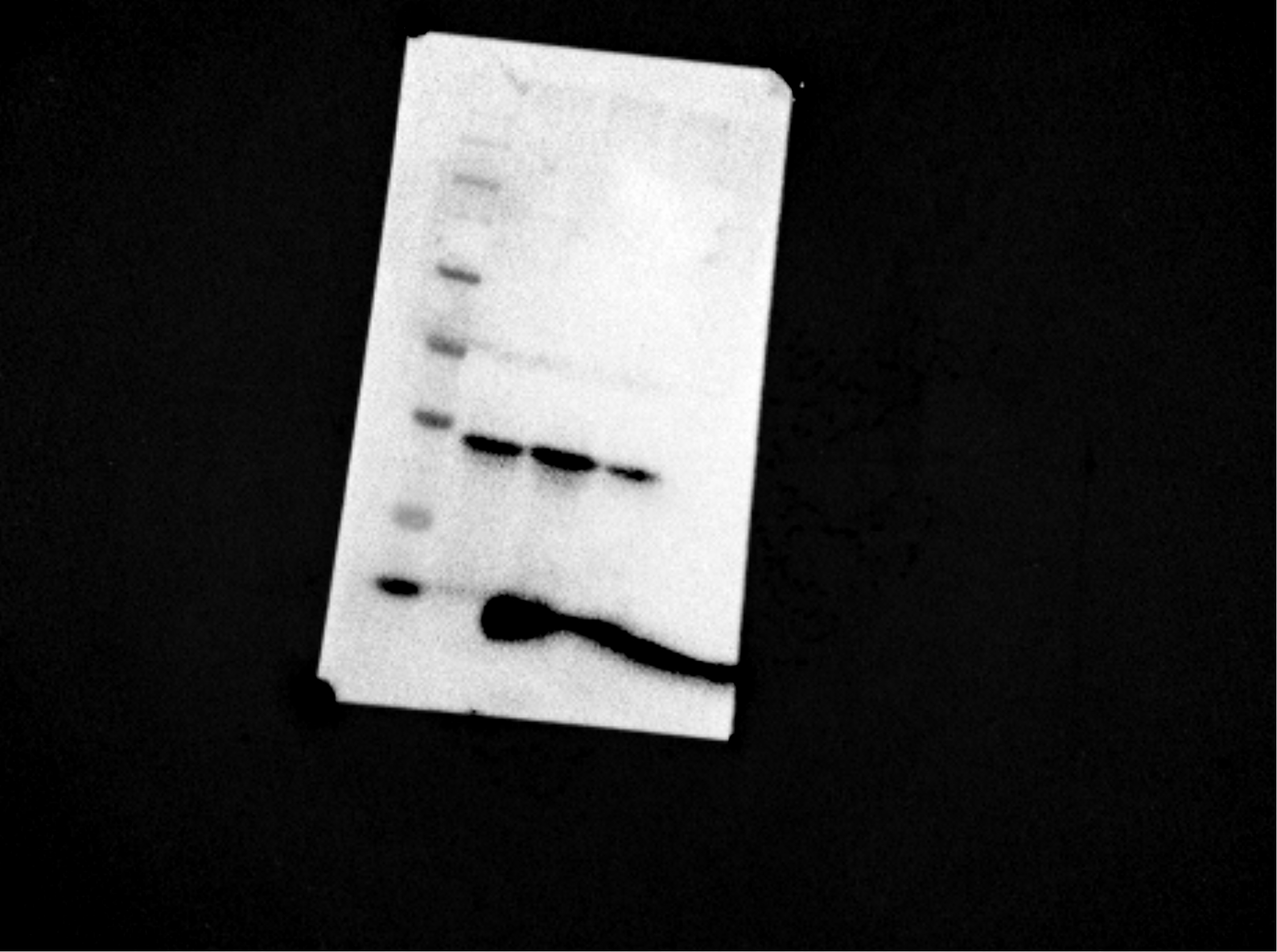


## Full and uncropped western blots of figure 9B-3.jpg


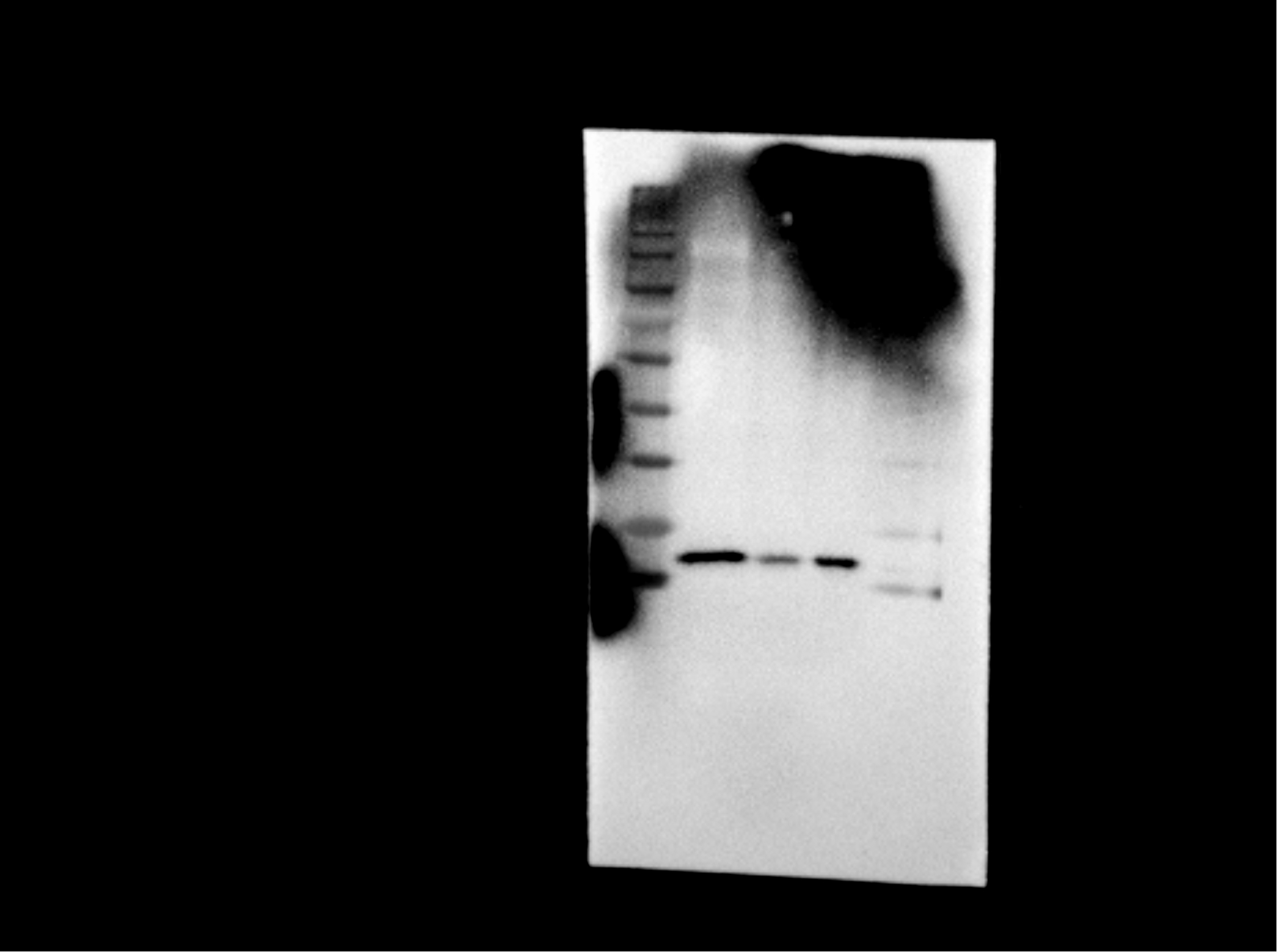


## Full and uncropped western blots of figure 9B-4.jpg


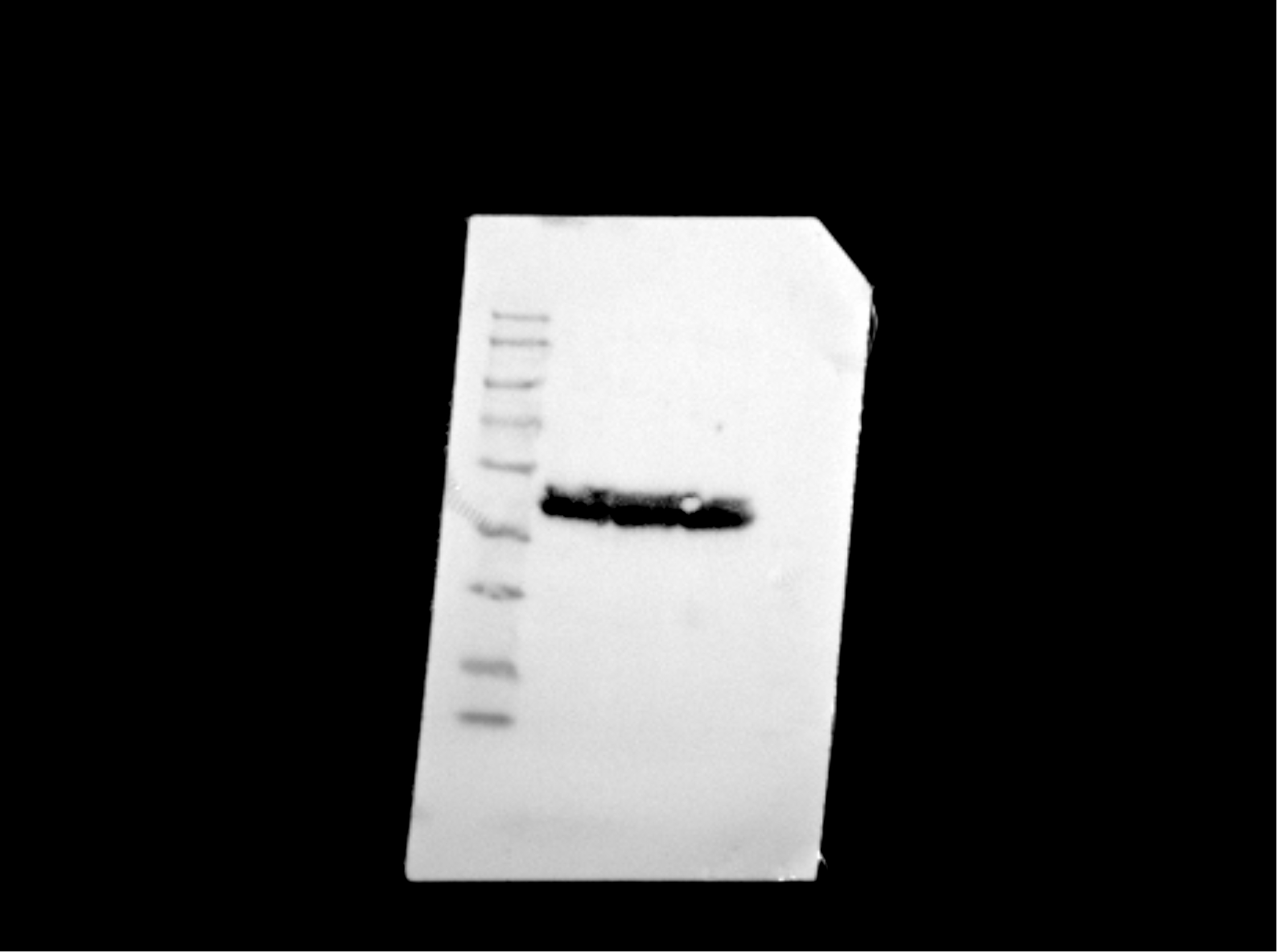


## Full and uncropped western blots of figure s3B-1.jpg


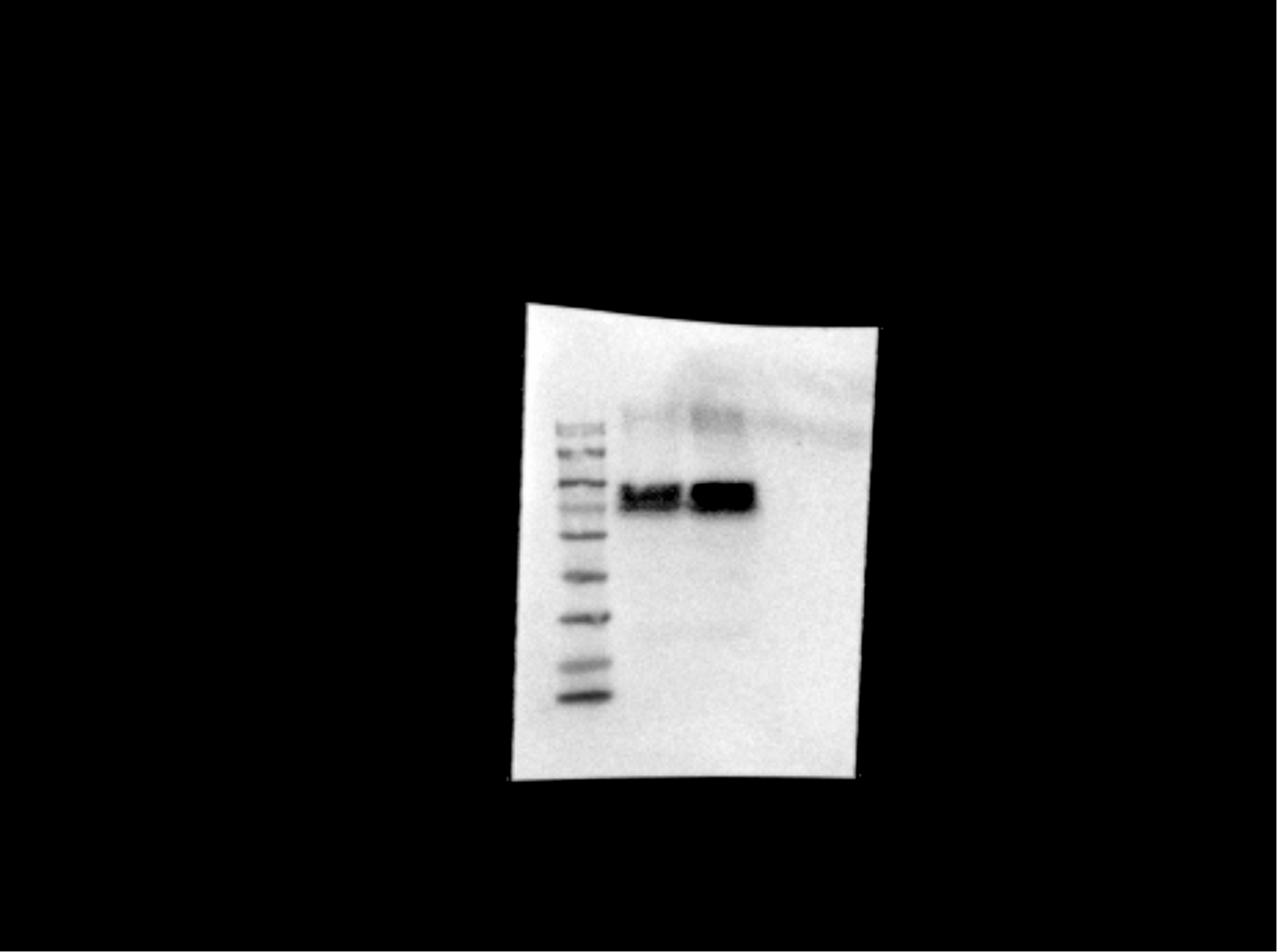


## Full and uncropped western blots of figure s3B-2.jpg


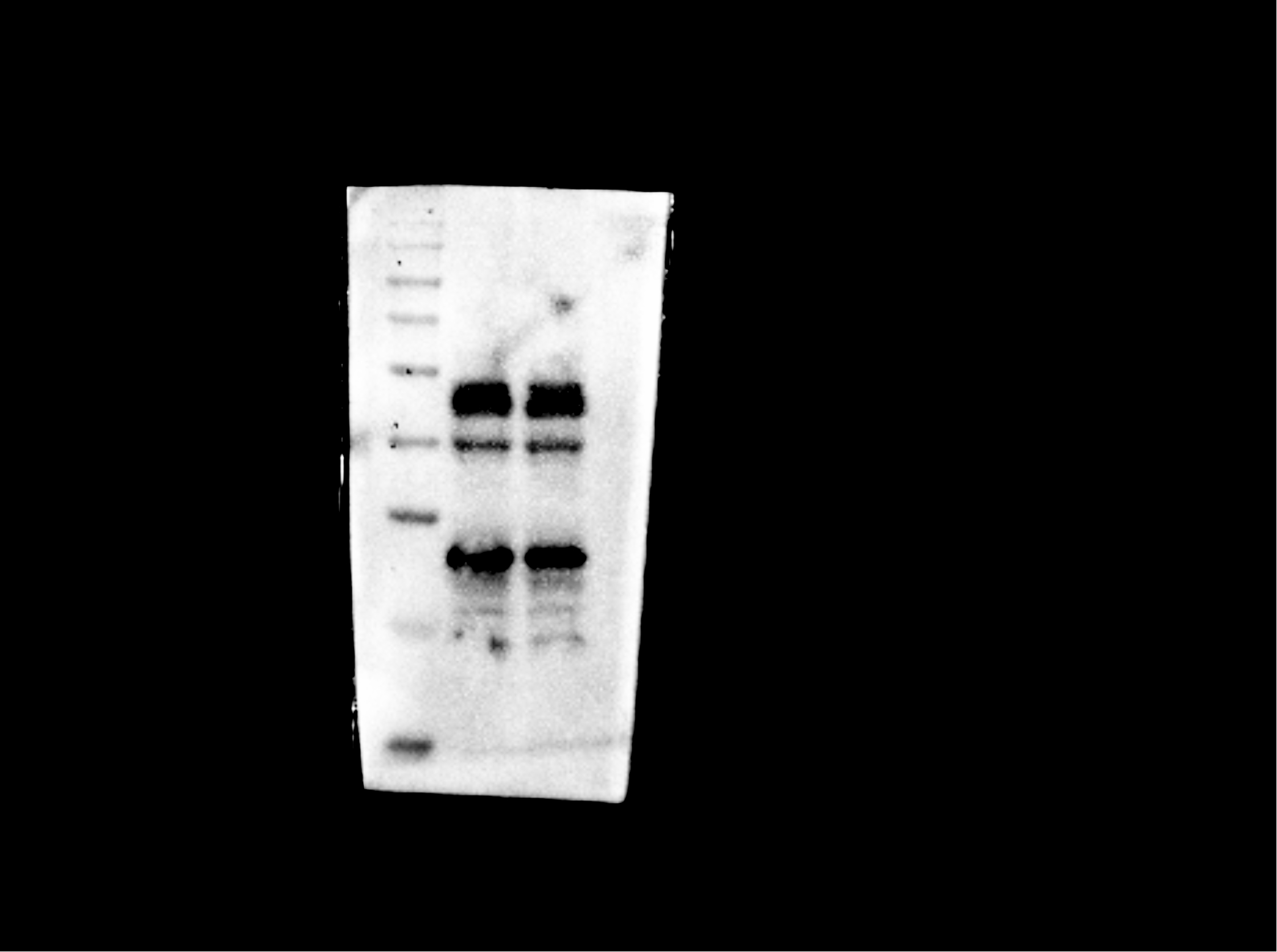


## Full and uncropped western blots of figure s3D-1.jpg


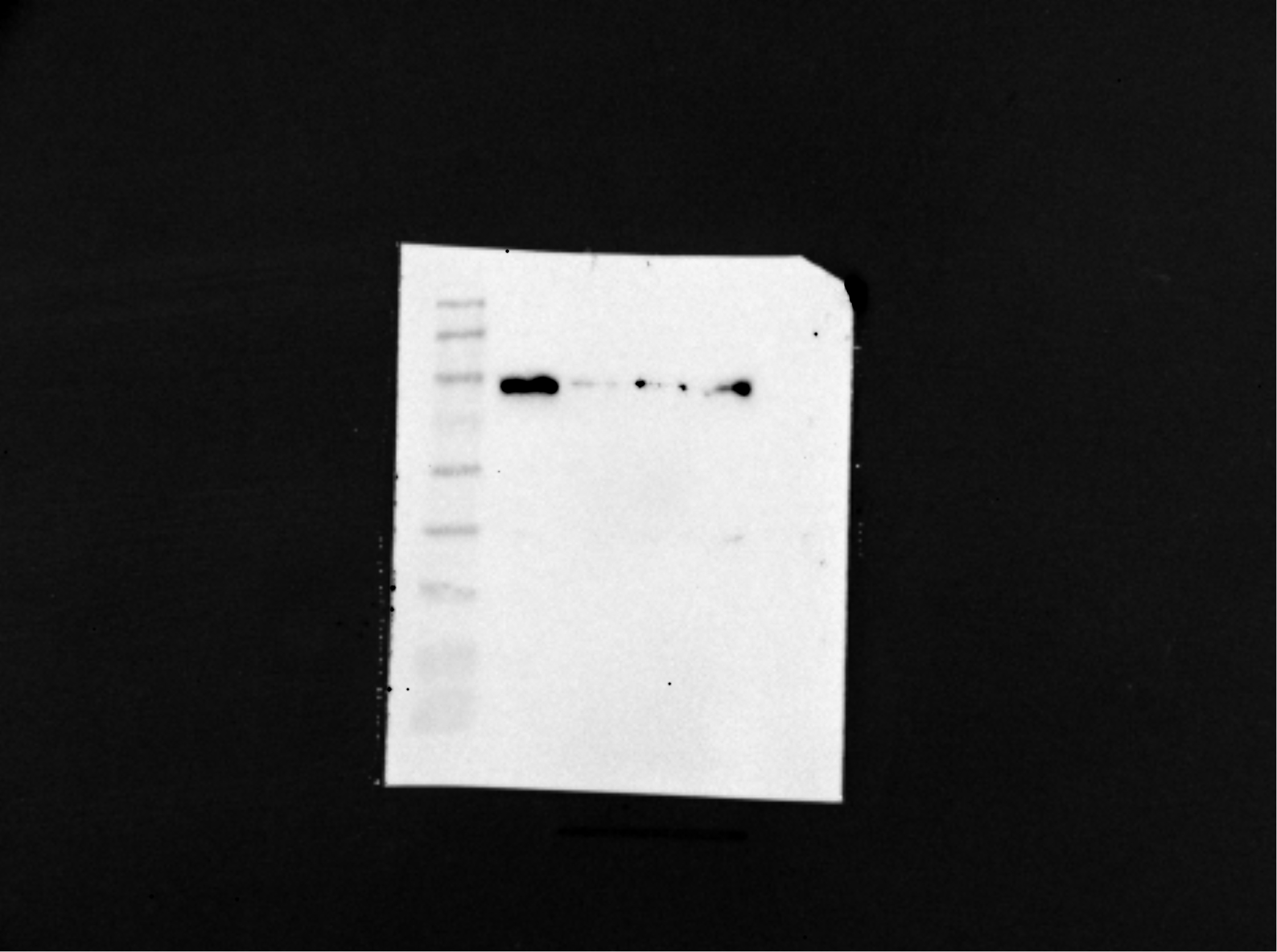


## Full and uncropped western blots of figure s3D-2.jpg


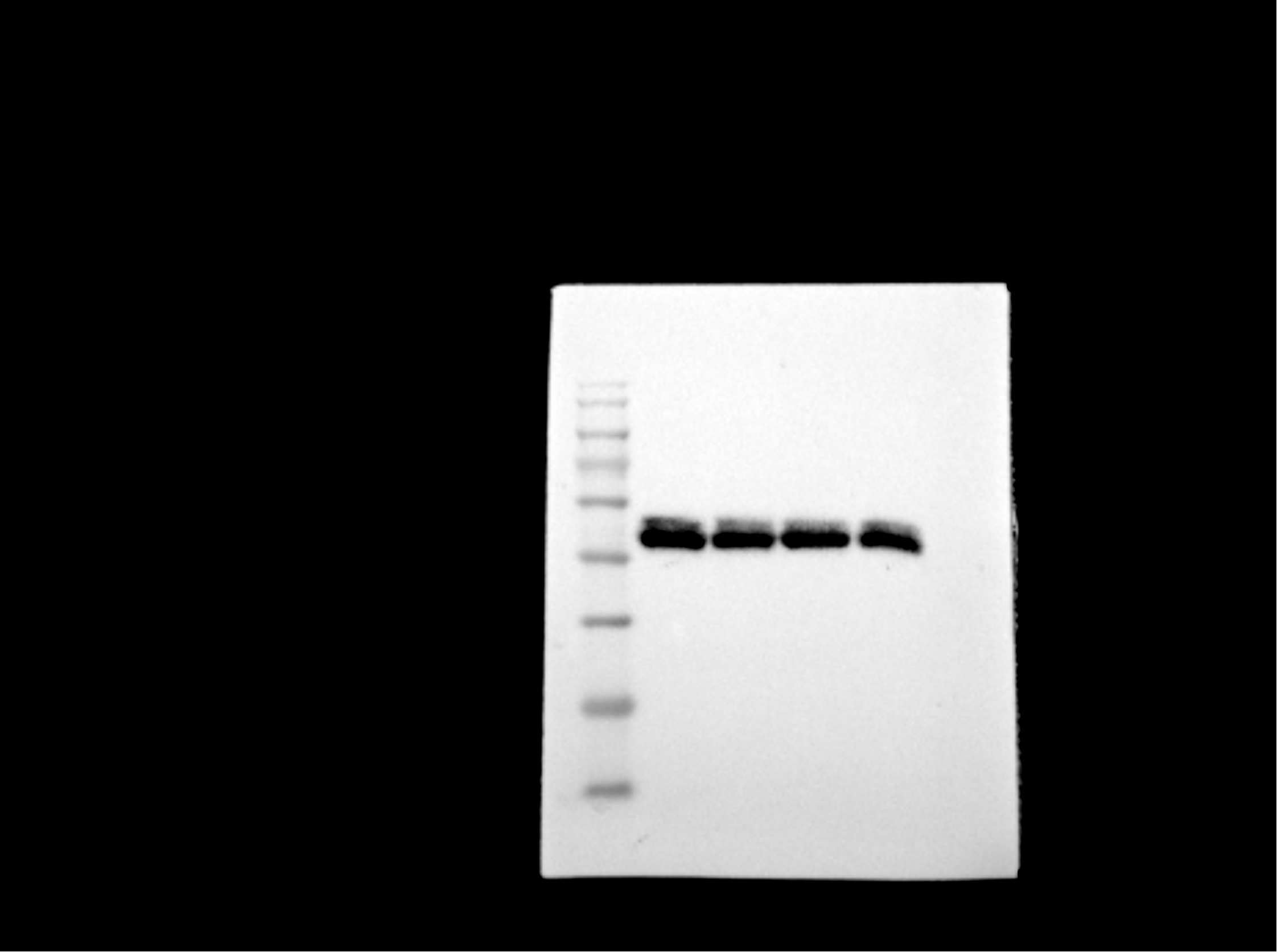


## Full and uncropped western blots of figure s4B-1.jpg


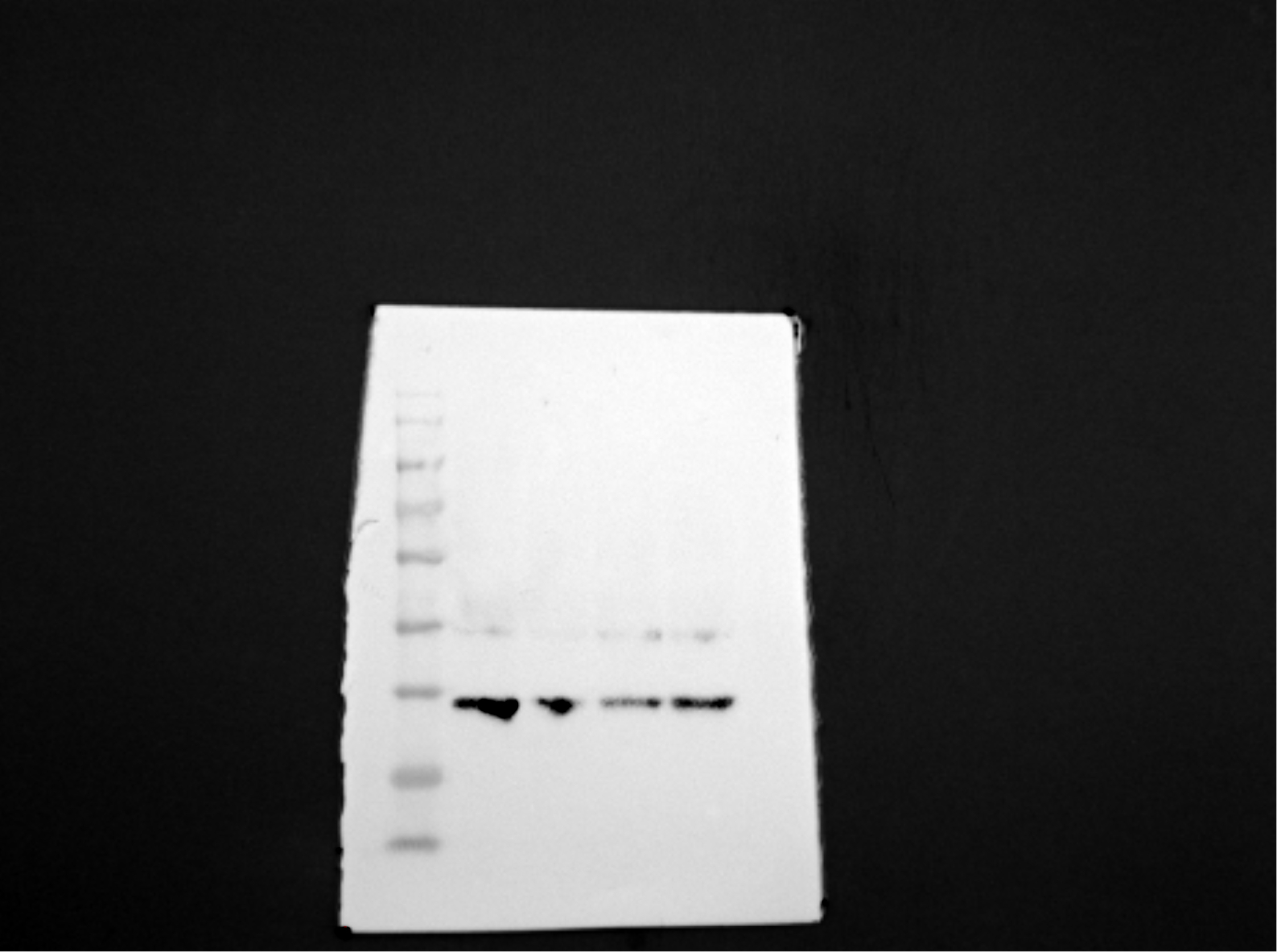


## Full and uncropped western blots of figure s4B-2.jpg


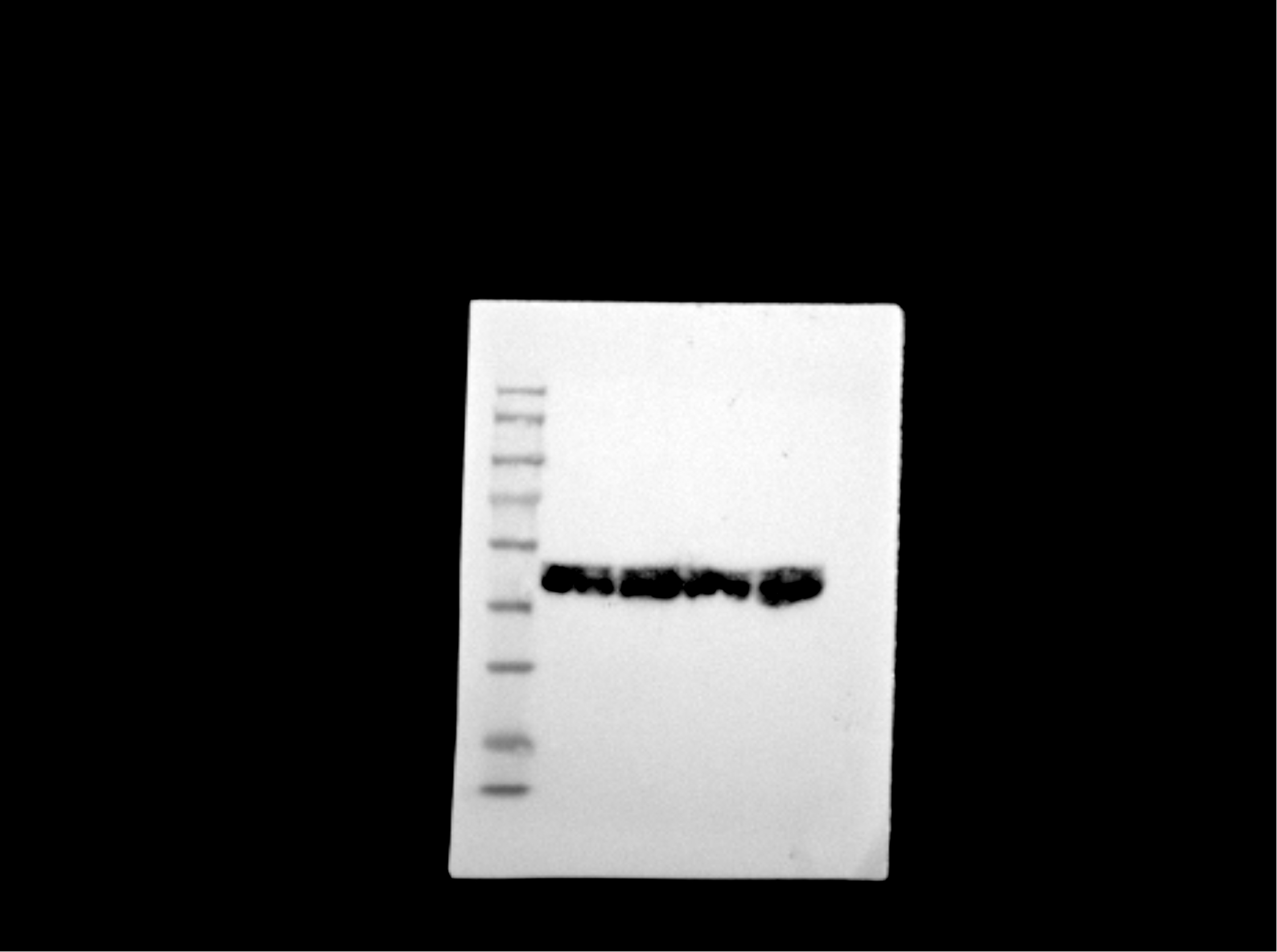

Supplement: Supplementary file 2 — western blots [file 41419_2025_8167_MOESM2_ESM.docx]
